# ZDHHC17‐Mediated CDK4 Palmitoylation Drives Cell Cycle Progression and Orchestrates Cancer Immune Surveillance

**DOI:** 10.1002/advs.75693

**Published:** 2026-05-14

**Authors:** Zekang Wang, Peipei Song, Xueji Wu, Lei Wang, Wei Xie, Pinning Feng, Chao Cheng, Jianping Guo

**Affiliations:** ^1^ Center of Thoracic Surgery the First Affiliated Hospital Sun Yat‐sen University Guangzhou Guangdong China; ^2^ Institute of Precision Medicine the First Affiliated Hospital Sun Yat‐sen University Guangzhou Guangdong China; ^3^ Department of Laboratory Medicine The First Affiliated Hospital Sun Yat‐Sen University Guangzhou China

**Keywords:** CDK4, cell cycle, immunotherapy, palmitoylation, ZDHHC17

## Abstract

Uncontrolled cell cycle progression is a hallmark of cancer, tightly regulated by both intrinsic and extrinsic stimuli. However, the role of fatty acids, in particular palmitic acid, in cell cycle control remains incompletely understood. Here, we observe that inhibition of protein palmitoylation by administrating 2‐bromohexadecanoic acid (2‐BP) or depleting *ZDHHC17*, leads to profound cell cycle arrest. Mechanistically, ZDHHC17 palmitoylates CDK4, thereby facilitating its interaction with cyclin D1 (encoded by CCND1), a process depending on TRAF6‐mediated K11‐linked ubiquitination of CDK4. While, blockade of either palmitoylation or ubiquitination markedly reduces CDK4 kinase activity, resulting in cell cycle arrest and suppressed tumor growth. Furthermore, *Zdhhc17*‐depletion displays reduced cell cycle progression and immune response in a high fat diet (HFD)‐feeding mouse model. Clinically, high ZDHHC17 expression is positively correlated with non‐response to anti‐PD‐1 therapies in cancer patients, partially due to CDK4‐mediated repression of PD‐L1. Thereby, we propose a rational combination strategy of employing CDK4 inhibitors with immune checkpoint blockers (ICBs) to overcome ZDHHC17‐driven cancers. In sum, our study uncovers a novel cell cycle control mechanism by ZDHHC17‐mediated palmitoylation and TRAF6‐mediated ubiquitination of CDK4, presenting a potential therapeutic avenue by targeting the ZDHHC17‐TRAF6‐CDK4 axis for cell cycle dysregulated cancers.

## Graphical abstract

ZDHHC17 palmitoylates CDK4, which together with TRAF6‐mediated ubiquitination, drives cell cycle progression and immune surveillance, revealing a rational combination of CDK4 inhibitors with immune checkpoint blockers for ZDHHC17‐driven cancers.

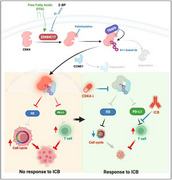

## 1 Introduction

Uncontrolled cell cycle progression is a hallmark of cancer [1]. Consequently, systematic treatments such as chemotherapy (e.g., taxol) and small‐molecule inhibitors by targeting the cell cycle have been extensively explored and approved for various cancer therapies as the first‐in‐class drugs [2]. Of note, CDK4/6 inhibitors, such as Palbociclib (Pal) and Abemaciclib (Abe), have shown efficacy in treating hormone receptor (HR) positive breast cancer [3, 4]. However, both intrinsic and acquired resistance to CDK4/6 inhibitors have emerged, driven by feedback regulation or crosstalk with other signaling pathways [5]. To overcome these challenges, combination therapies by incorporating CDK inhibitors with other targeted agents, such as PI3K/AKT inhibitors [6], BRD inhibitors [7], or hormone inhibitors [8], have been accumulating developed. More recently, CDK4/6 inhibitors have been found to significantly enhance immunotherapeutic responses, partially by promoting PD‐L1 expression in tumor cells [9] or inducing cytokine secretion that modulates the tumor microenvironment (TME) [10]. As a result, combining CDK4 inhibitors with immune checkpoint blockade (ICB) therapies has demonstrated improved efficacy revolutionarily [11]. Despite these advances, further investigation on the regulation of CDK4 and mechanisms of acquired resistance to its inhibitors remains an urgent clinical need.

The cell cycle is tightly regulated through multiple layers of control, including key activators such as CDK/cyclin complexes, and checkpoint regulators such as p53, p21, and p16, which are operated at both transcriptional and post‐translational (PTM) levels [12]. Among these regulators, the binding of CDKs to their corresponding cyclins, such as CDK4/6 with cyclin D1, CDK1 with cyclin A1, and CDK2 with cyclin B, plays a well‐defined role in driving cell cycle progression [13, 14]. Meanwhile, the E3 ligases such as SKP2, FBW7, and Anaphase‐Promoting Complex/Cyclosome (APC/C) dynamically altered following the cell cycle, could degrade according cyclins and disturb CDK kinase activity, thus tightly controlling cell cycle progression [15]. As such, inhibitors that selectively target specific CDKs or disrupt CDK‐cyclin interactions have gained significant attention in cancer treatment [16]. Thus, identification of the regulation of CDK or its interaction with cyclins would provide valuable information for targeting this important pathway.

Besides canonical growth factors, recently, metabolites or their bypass products, such as lactate, have shown the capability to promote cell cycle [17]. Meanwhile, palmitic acid (PA) and it‐mediated protein palmitoylation, have been well‐established in regulating critical pathophysiological processes [18], including ferroptosis [19], innate immune responses [20, 21], and pyroptosis [22]. However, the role of PA and palmitoylation in cell cycle control remains unexplored. Here, we identify palmitoyl‐transferase ZDHHC17‐mediated CDK4 palmitoylation as a key controller of cell cycle and subsequent immune responses, in a TRAF6‐mediated CDK4 K11‐linked ubiquitination‐dependent manner. These findings highlight the potential therapeutic strategy of combining CDK4 inhibitors with ICBs for treating ZDHHC17‐driven cancers.

## 2 Results

### 2.1 Repression of Palmitoyl‐Transferases by 2‐BP Induces Cell Cycle Arrest

**FIGURE 1:**
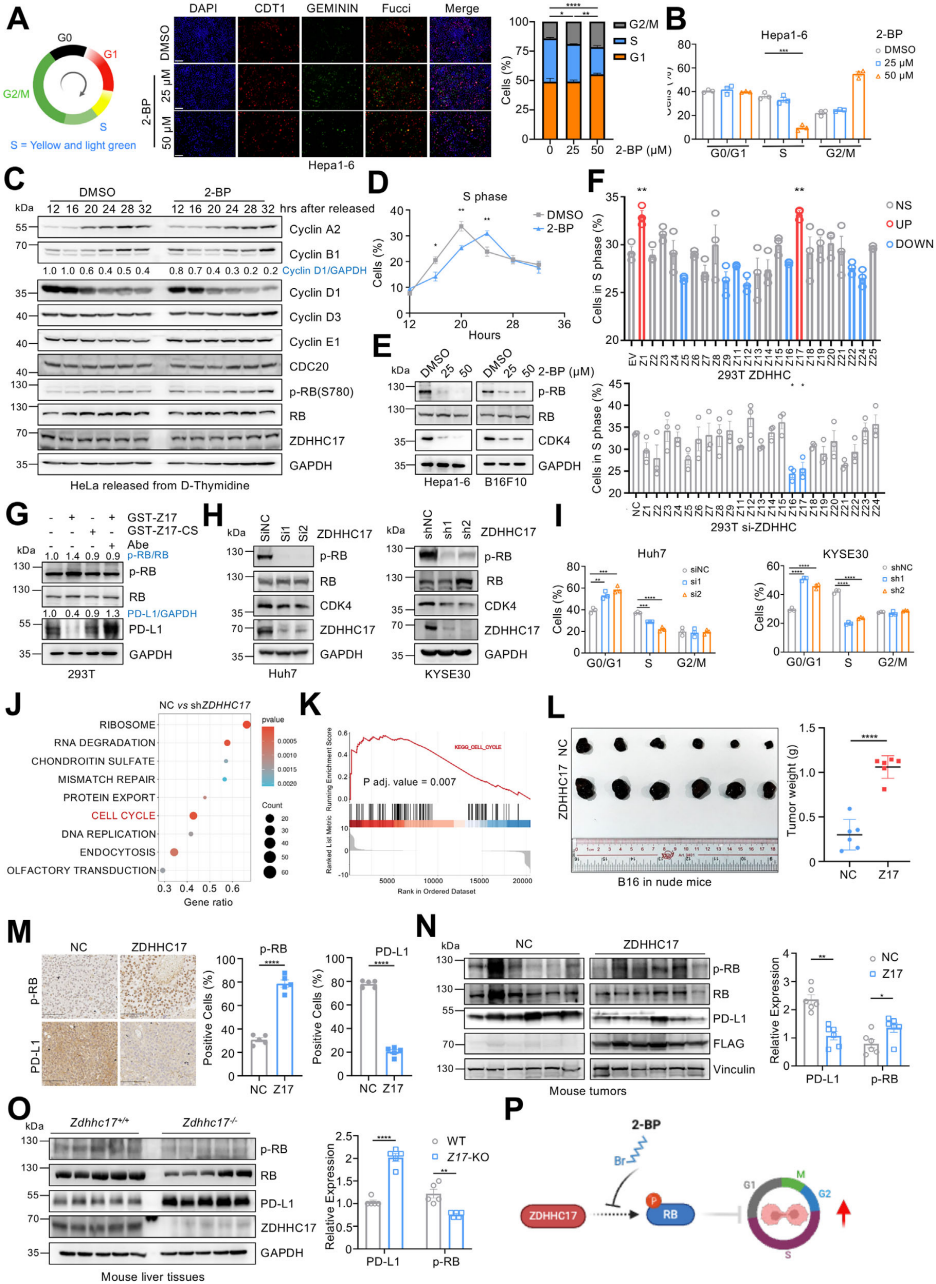
ZDHHC17 promotes cell cycle and decreases PD‐L1 expression in a palmitoyl‐transferase dependent manner. (A) Fluorescence imaging of Fucci infected Hepa1‐6 cells treated with 2‐Bromopalmitic acid (2‐BP) (0, 25, 50 µm) for 12 h (mean ± SEM, *n* = 3, one‐way ANOVA). Scale bar, 100 µm. (B) Flow cytometry analysis of cell cycle in Hepa1‐6 cell treated with 2‐BP (0, 25, 50 µm) for 12 h (mean ± SEM, *n* = 3, one‐way ANOVA, ^***^ *p* < 0.001). (C,D) Western blot (WB) analysis of whole‐cell lysates (WCL) derived from HeLa cells synchronized in G1/S boundary by double‐Thymidine and treated with 2‐BP (50 µm) for 12 h after released (C). The S phase cell‐cycle profiles were monitored by Flow cytometry (mean ± SEM, *n* = 3, Student's *t*‐test, ^*^ *p* < 0.05, ^**^ *p* < 0.01)) (D). (E) WB analysis of WCL derived from Hepa1‐6 and B16F10 cells treated with 2‐BP (0, 25, 50 µm) for 12 h. (F) Flow cytometry analysis of cell cycle in HEK293T cells transfected with ZDHHC family plasmids (up panel) or SiRNAs (down panel) for 24 h. (G) WB analysis of WCL derived from HEK293T cells transfected with indicated constructs, treated with or without Abemaciclib (1 µm) for 12 h. (H,I) WB analysis of WCL derived from Huh7 cells transfected with different siRNAs and KYSE30 cells infected with different shRNAs and selected with puromycin for 72 h. The resulting cells were subjected for S phase cell‐cycle profiles by Flow cytometry (mean ± SEM, *n* = 3, Student's *t*‐test, ^**^ *p* < 0.01, ^***^ *p* < 0.001, ^****^ *p* < 0.0001) (I). (J,K) KYSE30 cell lines generated in (H) were subjected to RNA sequencing, and for Kyoto Encyclopedia of Genes and Genomes (KEGG) and Gene Set Enrichment Analysis (GSEA) analysis (J). The correlation of ZDHHC17 expression with the cell cycle was analyzed (K). (L‐N) Tumor image and weights between B16F10 cells infected with vector or ZDHHC17 lentivirus (mean ± SEM, *n* = 6, Student's *t‐* test, ^****^ *p* < 0.0001) were represented (L). Representative images of immunohistochemistry (IHC) staining of paraffin section derived from tumor tissues, and the relative staining density were analyzed (mean ± SEM, *n* = 5, Student's *t*‐test, ^****^ *p* < 0.0001). Scale bar, 100 µm (M). WB analysis of WCL derived from tumor tissues, and the relative protein levels were analyzed (mean ± SEM, *n* = 6, Student's *t*‐test, ^*^ *p* < 0.05, ^**^ *p* < 0.01) (N). (O) WB analysis of WCL derived from liver tissues of *Zdhhc17*‐KO (*Zdhhc17^−/−^*) and counterpart mice. The relative protein levels were analyzed (mean ± SEM, *n* = 5, Student's *t*‐test, ^**^ *p* < 0.01, ^****^ *p* < 0.0001). (P) The illustration of ZDHHC17 involves in cell cycle control in a palmitoylation dependent manner.

2‐BP has been wildly used as a canonical pan‐inhibitor for ZDHHC palmitoyl‐transferases, and exhibits a potential role in repressing tumorigenesis [23]. Here, we observed that 2‐BP could suppress cellular proliferation (measured with CCK8 assay) (Figure S1A), but with mildly affecting on cellular apoptosis (measured with Annexin V‐PE/7AAD assay) (Figure S1B), in diverse type of cell lines. Strikingly, 2‐BP readily promoted cell cycle arrest across these cell lines measured with EDU assays (Figure S1C). To exploit the potential role of 2‐BP in cell cycle control, we employed a fluorescent ubiquitination‐based cell cycle indicator (Fucci) system, which is commonly used as a sophisticated technology to easily determine G1 and/or S and/or G2/M phases of the cell cycle (Figure 1A) [24]. We observed that 2‐BP mainly induced cell cycle arrest by decreasing the subpopulation of S‐phase measured with both Fucci (Figure 1A) and flow cytometry assays (Figure 1B). Furthermore, cell cycle synchronization assays revealed that 2‐BP markedly deferred S‐phase progression, as confirmed by established cell cycle markers (Figure 1C) and flow cytometry analyses (Figure 1D) from a release of cell synchronization by double thymidine block. Since phosphorylation of retinoblastoma protein (p‐RB) is a checkpoint of G1/S phase [25], we observed that 2‐BP reduced p‐RB (Figure 1E and Figure S1D). These findings together suggest that 2‐BP negatively regulates cell cycle progression.

### 2.2 ZDHHC17 Drives Cell Cycle Progression

Although 2‐BP has been shown to influence numerous cellular processes, it remains a widely used and well‐characterized inhibitor of ZDHHC palmitoyl‐transferases [23]. Thus, we sought to investigate the potential ZDHHC in controlling cell cycle. To this end, we either overexpressed or depleted various ZDHHC family members and assessed their effects on cell cycle progression by flow cytometry assays. Among them, overexpression of ZDHHC1 and ZDHHC17 significantly increased, while depletion of *ZDHHC16* and *ZDHHC17* repressed the proportion of cells in S‐phase (Figure 1F). As a result, ZDHHC17 has been characterized to influence cell cycle and focused for further evaluation. Further validation showed that ZDHHC17, but not its catalytically inactive variant (C467S), could enhance p‐RB (Figure 1G and Figure S1E,F). Meanwhile, depletion of *ZDHHC17* attenuated p‐RB levels (Figure 1H and Figure S2A–C), accompanied by cell cycle arrest measured with flow cytometry (Figure 1I and Figure S2D,E), CCK‐8 assays (Figure S2F), EDU staining (Figure S2G), and Fucci assays (Figure S2H). Importantly, ZDHHC17 deficiency has been significantly involved in cell cycle arrest transcriptionally measured with RNA‐seq (Figure 1J,K).

Next, we constructed ZDHHC17‐overexpressed (OE) stable cell lines, and observed that enforcing expression of ZDHHC17 elevated p‐RB levels and led to faster cell proliferation, colony formation, and cell cycle (Figure S3A–D). To confirm the potent role of ZDHHC17 in regulating cell cycle in vivo, we performed xenografted mouse models. In agreement with previous findings [23], overexpression of ZDHHC17 promoted tumor growth in an immune‐deficient mouse model (Figure 1L), which correlated with increased p‐RB levels, confirmed by both immunoblotting and immunohistochemistry (IHC) (Figure 1M,N). Strikingly, ZDHHC17‐OE displayed mildly declined tumor growth rate in a syngenetic C57BL/6 mouse model (Figure S3E–G), possibly due to ZDHHC17‐induced immune surveillance. Consistently, flow cytometry analysis showed that more active CD8+ T cells infiltrated into the tumor microenvironment (TME) (Figure S3H–J). To further investigate the role of ZDHHC17 in cell cycle regulation, we utilized *Zdhhc17* knockout (KO) mice [23], and harvested different tissues. The results showed that tissues, including liver, lung, spleen, and heart derived from *Zdhhc17*‐KO mice exhibited reduced p‐RB levels (Figure 1O and Figure S3K–M). Together, these findings suggest ZDHHC17 as an oncogenic factor by promoting cell cycle progression in a palmitoyl‐transferase dependent manner (Figure 1P).

### 2.3 ZDHHC17 Catalyzes CDK4 Palmitoylation to Promote Tumorigenesis

**FIGURE 2:**
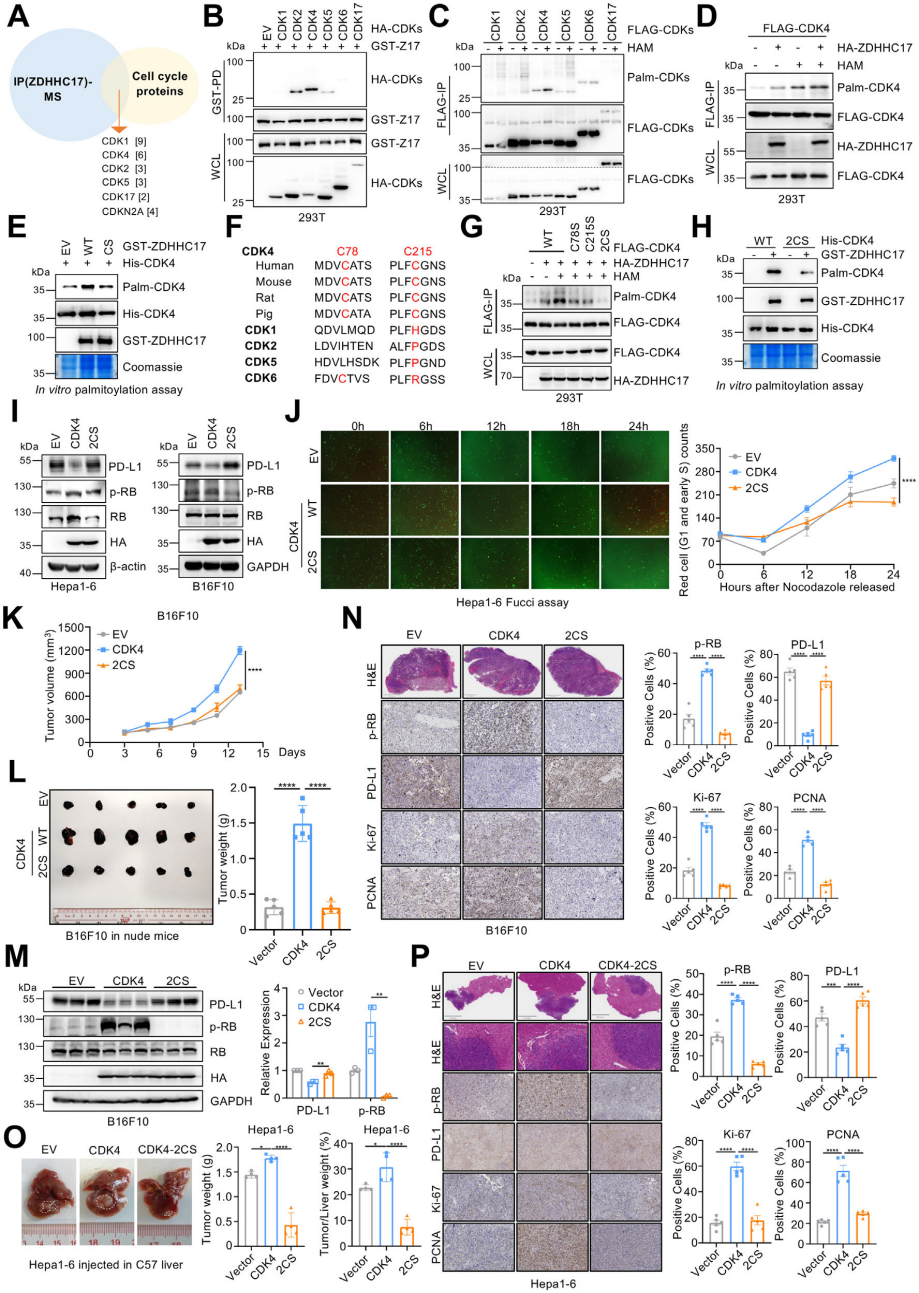
ZDHHC17 palmitoylates and activates CDK4 to accelerate the cell cycle. (A) Veen plot showed the overlap between MS analysis of ZDHHC17 binding proteins, and the cell cycle associated protein, and potential proteins have been listed. (B) WB analysis of IP and WCL derived from HEK293T cells transfected with indicated constructs. (C) ABE palmitoylation assay was performed to detect palmitoylation of indicated protein. (D) ABE palmitoylation assay was performed to detect CDK4 palmitoylation. (E) WB analysis of palmitoylated CDK4 by in vitro palmitoylation assay. (F) Alignment of the amino acids of CDKs and CDK4 in different species. (G,H) ABE palmitoylation assay was performed in cells (G) or in vitro (H). (I) WB analysis of WCL derived from Hepa1‐6 or B16F10 infected with lentivirus encoding indicated constructs and selected with hygromycin for 72 h. (J) Fluorescence imaging of Hepa1‐6 cells infected with Fucci followed by vector, CDK4, and CDK4‐2CS lentivirus treated with Nocodazole (330 nm) for 12 h. The red cell numbers were counted and plotted (mean ± SEM, *n* = 3, two‐way ANOVA, ^****^ *p* < 0.0001). (K,L) B16F10 cell lines generated in (I) were subjected for xenografted mouse model, and the growth curves (K) and tumor weights (L) between different groups were analyzed (mean ± SEM, *n* = 5, two‐way ANOVA for growth curves; one‐way ANOVA for tumor weights, ^****^ *p* < 0.0001). (M,N) WB analysis of WCL derived from tumor tissues, and the relative protein levels were analyzed (mean ± SEM, *n* = 3, one‐way ANOVA, ^**^ *p* < 0.01) (M). Representative images of immunohistochemistry (IHC) staining of paraffin section derived from tumor tissues, and the relative staining density were analyzed (mean ± SEM, *n* = 5, one‐way ANOVA, ^****^ *p* < 0.0001). Scale bar of IHC images, 100 µm (L). WB analysis of WCL derived from tumor tissues, and the relative protein levels were analyzed (mean ± SEM, *n* = 3, one‐way ANOVA, ^**^ *p* < 0.01) (N). (O,P) Representative images of orthotopic liver cancer model via Hepa1‐6 cells infected with vectors, CDK4 and CDK4‐2CS in C57BL/6 mice. Tumor weights and tumor/Liver weights percentage were calculated (mean ± SEM, *n* = 5, one‐way ANOVA, ^*^ *p* < 0.05, ^****^ *p* < 0.0001). IHC staining analysis of harvested tumor tissues (mean ± SEM, *n* = 5, Student's *t*‐test, ^***^ *p* < 0.001, ^****^ *p* < 0.0001) (O). Representative images of immunohistochemistry (IHC) staining (P).

To investigate the mechanism of ZDHHC17 in regulating the cell cycle, we performed mass spectrometry‐based analysis to identify ZDHHC17 interaction proteins. Among them, we have focused on the cell cycle‐associated proteins, including cyclin‐dependent kinases (CDKs), CDK1, CDK2, CDK4, and CDK5, as well a G1/S checkpoint protein CDKN2A (encoded p16), were present in the ZDHHC17‐IP products (Figure 2A). Further binding assays demonstrated that CDK4, to a lesser extent of CDK2, but not other CDKs we measured interacted with ZDHHC17 (Figure 2B). Since the palmitoylation activity of ZDHHC17 was involved in cell cycle control (Figure 1G), thus, we performed ABE‐based palmitoylation assays, and observed that CDK4, but much less extent of other CDKs, underwent ZDHHC17‐mediated palmitoylation (Figure 2C). Specifically, functions of ZDHHC17 in regulating p‐RB, and PD‐L1, another downstream effector of CDK4 [13, 14], could largely be blocked by CDK4/6 inhibitor (Figure 1G and Figure S1E), suggesting that ZDHHC17 positively regulates CDK4. In consistent with this finding, PD‐L1 levels could tightly negatively regulated by ZDHHC17 both in cell and in *vivo* conditions (Figure 1M–O and Figures S2A–C, S3A, S3K–M). Furthermore, ZDHHC17 but not its catalytic dead form (C467S) could promote CDK4 palmitoylation both in cells and in vitro (Figure 2D,E). By integrating point‐mutation and webtool‐based analysis [26], two cysteine residues (C78 and C215) were predicted as potential palmitoylation residues, and displayed conserved across multiple species, but not in other CDK family members (Figure 2F). As a result, mutation of these cysteine residues to serine (S) significantly attenuated ZDHHC17‐induced CDK4 palmitoylation both in cells (Figure 2G and Figure S4A), and in vitro palmitoylation assays (Figure 2H), with a mild effect on its conformation measured with cellular thermal shift assay (CETSA) (Figure S4B) [20].

Next, we investigated the role of CDK4 palmitoylation in cell cycle regulation. The results showed that enforced expression of WT, but not the palmitoylation‐deficient CDK4 (C78S and C215S, termed 2CS), increased p‐RB and decreased PD‐L1 expression (Figure 2I and Figure S4C). Meanwhile, WT but not the 2CS mutant CDK4 promoted cell cycle progression measured via Fucci fluorescence imaging (Figure 2J) and flow cytometry (Figure S4D), coupled with increased cell viability, proliferation, and colony formation (Figure S4E–H). To assess the in vivo role of CDK4 palmitoylation, we employed a xenografted mouse model with B16F10 cells. Among that, enforced expression of WT, but not 2CS mutant CDK4, enhanced tumor growth (Figure 2K,L). In addition, WT‐CDK4 expression markedly enhanced cell proliferation, p‐RB levels, and decreased PD‐L1 expression (Figure 2M,N). Moreover, we also evaluated the role of these CDK4 mutations in a syngeneic orthotopic liver mouse model by injecting Hepa1‐6 cells into liver. The results showed that CDK4 expression could markedly enhance Hepa1‐6‐based tumor growth in mouse livers compared with the tumors derived from palmitoylation‐deficient (2CS) CDK4 expressing cells (Figure 2O). In line with nude mouse model, WT CDK4, but not 2CS mutant, could elevate p‐RB levels, and enhance cell cycle and proliferation (Ki67, PCNA), as well as decreased PD‐L1 expression (Figure 2P). These findings imply that palmitoylation modification promotes CDK4 activity in controlling cell cycle and PD‐L1 expression.

### 2.4 Palmitoylation Stabilizes CDK4 and Enhances its Interaction With Cyclin D1

To evaluate the mechanism of palmitoylation‐mediated CDK4 activation, we initially assessed CDK4 subcellular localization, based on the well‐established function of palmitoylation in protein membrane targeting [23, 27]. Immunofluorescence (IF) analysis revealed that enforced expression of ZDHHC17 or 2CS mutation of CDK4 could not alter its cellular localization (Figure S5A,B). Interestingly, we found that palmitoylation played a potent role in regulating CDK4 expression, as CDK4 levels was significantly reduced upon 2‐BP administration (Figure 1E) or *ZDHHC17* depletion (Figure 1H), with a mild effect on its mRNA levels (Figure S5C), suggesting this is possibly through affecting CDK4 protein stability. While CDK4 has been recognized as a relative stable protein, and sometimes is used as control, E3 ligase like SKP2 has been identified regulating CDK4 stability [28]. Thus, we sought to investigate whether palmitoylation could regulate CDK4 stability. To this end, cycloheximide (CHX) chase assays were used and confirmed that ZDHHC17 overexpression prolonged CDK4 half‐life (Figure S5D), while *ZDHHC17*‐depletion shortened its half‐life (Figure S5E). In consistent with this finding, palmitoylation‐deficient mutant (2CS) strongly shortened CDK4 stability (Figure S5F), which could not be stabilized by ZDHHC17 (Figure S5D). These findings together suggest that ZDHHC17 plays a potent role in stabilizing CDK4 in a palmitoylation dependent manner.

**FIGURE 3:**
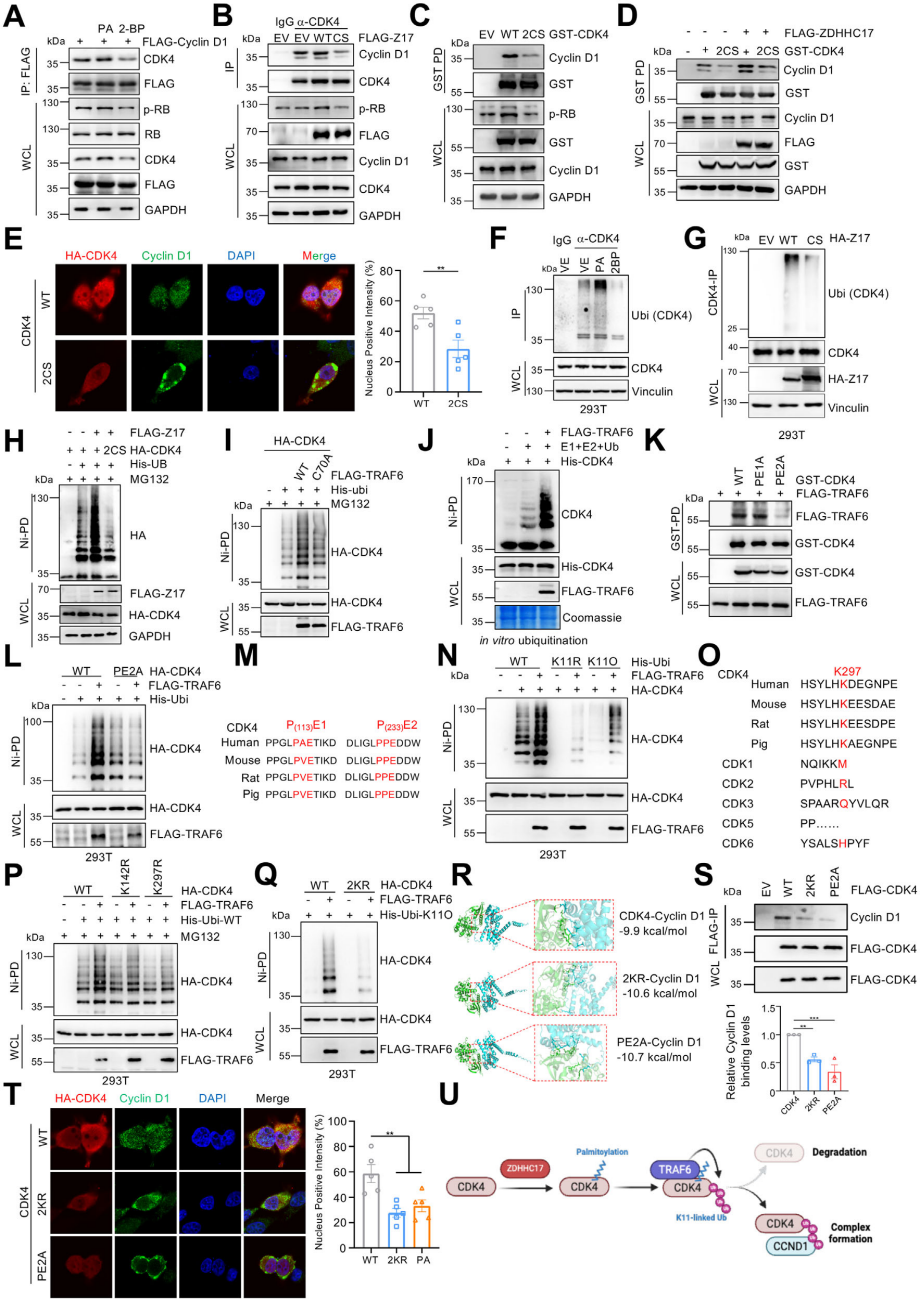
ZDHHC17 facilitates CDK4 interaction with Cyclin D1. (A) WB analysis of IP and WCL derived from HEK293T cells transfected with FLAG‐Cyclin D1 and treated with PA (100 µm) or 2‐BP (50 µm) for 12 h. (B–D) WB analysis of IP, GST pulldown, and WCL derived from HEK293T cells transfected with indicated constructed. (E) HEK293T cells were transfected with different constructs for 24 h, then subjected to IF analysis (mean ± SEM, Student's *t*‐test, ^**^ *p* < 0.01). Scale bar, 5 µm. (F,G) WB analysis of IP and WCL from anti‐CDK4 pull‐down products derived from HEK293T cells treated with PA or 2‐BP and B16F10 oeZDHHC17 cells. (H‐I) WB analysis of IP and WCL from Ni‐NTA pull‐down products derived from HEK293T cells transfected with indicated constructs and treated with MG132 (10 µm) for 12 h. (J) In vitro ubiquitination assays were performed with purified TRAF6 and His‐CDK4 by adding with E1, E2 (UBE1 and UbcH5b), and ubiquitin (obtained from UBbiotech) in the reaction buffer, which was subjected for IB analysis. (K) WB analysis of GST pulldown and WCL derived from HEK293T cells transfected with indicated constructed. (L,N) WB analysis of IP and WCL from Ni‐NTA pull‐down products derived from HEK293T cells transfected with indicated constructs. (M) Alignment of the amino acids of CDK4 in different species. (O) Homologically analysis of CDK4 K297 site in various species and CDK family members. (P,Q) WB analysis of IP and WCL from Ni‐NTA pull‐down products derived from HEK293T cells transfected with indicated constructs and treated with MG132 (10 µm) for 12 h. (R) Docking of the interaction between CDK4 and CCND1 with HDCOK. The binding affinity was predicted. (S) WB analysis of WCL derived from HEK293T cells transfected with indicated constructed. The relative protein levels were quantified (mean ± SEM, *n* = 3, Student's *t*‐test, ^**^ *p* < 0.01, ^***^ *p* < 0.001). (T) HEK293T cells were transfected with different constructs for 24 h, then subjected to IF analysis (mean ± SEM, Student's *t*‐test, ^**^ *p* < 0.01). Scale bar, 5 µm. (U) The illustration of ZDHHC17‐mediated CDK4 palmitoylation and subsequent TRAF6‐medited ubiqutination in regulation CDK4/Cyclin D1 interaction.

Since CDKs function is mainly dependent on their interaction with cyclin proteins [29], for instance, CDK4 performs its cell cycle regulation by interaction with cyclin D1 [30], we next assessed whether palmitoylation influences CDK4 interaction with cyclin D1. The results showed that CDK4‐cyclin D1 binding was enhanced by PA treatment and blocked by 2‐BP administration (Figure 3A). Consistently, expression of WT ZDHHC17 enhanced, whereas the 2CS mutant disrupted CDK4/cyclin D1 interaction in both exogenous and endogenous levels (Figure 3B and Figure S5G). To confirm the potential role of palmitoylation in mediating CDK4 interaction with cyclin D1, we observed that 2CS mutant attenuated CDK4 interaction with cyclin D1 (Figure 3C and Figure S5H,I). Meanwhile, ZDHHC17 enhanced WT but not 2CS CDK4 interaction with cyclin D1 (Figure 3D). Since CDK4 predominantly interacts with cyclin D1 to promote cyclin D1 nucleus translocation, in which activated CDK4‐cyclin D1 complex performs cell cycle control function [31], here we observed that WT CDK4, but not 2CS mutant, could promote cyclin D1 into nucleus to control cell cycle (Figure 3E and Figure S5J). To confirm the role of ZDHHC17 and the palmitoylation‐deficient mutant in CDK4 kinase activity, we performed in vitro kinase assays. The results showed that ZDHHC17 promoted WT, but not 2CS mutant, CDK4‐mediated phosphorylation of Rb in the presence of cyclin D1 (Figure S5K). These findings collectively demonstrate that ZDHHC17‐mediated palmitoylation stabilizes CDK4 and promotes its interaction with cyclin D1 in nucleus.

### 2.5 CDK4 Undergoes TRAF6‐Mediated K11‐Linked Ubiquitination

Next, we investigated whether palmitoylation regulates CDK4 stability and its interaction with cyclin D1 via ubiquitination modification. Strikingly, we observed that PA could enhanced, whereas 2‐BP could attenuate CDK4 ubiquitination in endogenous levels (Figure 3F). Meanwhile, ZDHHC17, but not its catalytically inactive mutant (CS), enhanced CDK4 ubiquitination (Figure 3G and Figure S6A), a modification typically associated with proteasome‐mediated degradation [32]. To clarify this unexpected result, we conducted ubiquitination assay using a palmitoylation‐deficient mutant CDK4‐2CS. The result showed that ZDHHC17 increased the ubiquitination of WT but not 2CS mutant CDK4 (Figure 3H). These findings suggest that this ubiquitination is dependent on ZDHHC17‐mediated CDK4 palmitoylation, and does not signal for degradation, instead, may play a role in regulating the interaction between CDK4 and cyclin D1 [33].

To identify the E3 ligase responsible for palmitoylation‐dependent CDK4 ubiquitination, we integrated mass spectrometry‐based profiling of CDK4 interaction partners with webtool‐based prediction analysis (Figure S6B) [34]. Among these potential E3 ligases and well‐established cell cycle related E3 ligases, TRAF6, with a lesser extent of DCAF13 and ITCH, has been identified strongly promoting CDK4 ubiquitination (Figure S6C,D). Additionally, among CDKs, TRAF6 promoted CDK4, a less extent of CDK6, ubiquitination modification (Figure S6E). Furthermore, the WT but not catalytic inactive TRAF6‐C70A, readily promoted CDK4 ubiquitination (Figure 3I). We further conducted in vitro ubiquitination assays and confirmed that TRAF6 directly promoted CDK4 ubiquitination (Figure 3J), suggesting CDK4 as a bona fide substrate of TRAF6. Next, CDK4, but not other CDK members we examined, has been defined to interact with TRAF6 (Figure S6F). Since TRAF6 typically interacts with its substrates via a conserved PXE domain [35], we scanned the amino acid sequence of CDK4, and identified two potential conserved PXE motifs (Figure 3K). To validate the roles of these PXE motifs, we generated PE‐to‐AA mutations and observed that mutations in second motif (PE2A) significantly impaired TRAF6 binding to CDK4 (Figure 3L). Consequently, PE2A mutant reduced TRAF6‐mediated CDK4 ubiquitination compared with the WT form (Figure 3M and Figure S6G), suggesting that TRAF6 directly regulates CDK4 ubiquitination through interacting with CDK4's PXE motif.

Given the non‐canonical role of TRAF6‐mediated ubiquitination besides stabilizing CDK4, we examined the specific ubiquitin linkage involved. Results showed that TRAF6‐mediated K11‐linked, but not K48‐linked chains, as previously reported by SKP2‐mediated CDK4 ubiquitination [28], significantly increased CDK4 ubiquitination (Figure 3M and Figure S6G). While, the Ubi‐K11R mutant blocked TRAF6‐mediated CDK4 ubiquitination (Figure 3N and Figure S6H–J), indicating that TRAF6‐mediated CDK4 K11‐linked ubiquitination. To explore the ubiquitination sites of CDK4 by TRAF6, we systematically mutated all lysine residues and performed ubiquitination assays (Figure S6K). Among them, K142R and K297R mutations markedly reduced CDK4 ubiquitination (Figure 3O and Figure S6K), and the double mutation of these sites (2KR) could prominently downregulate CDK4 K11‐linked ubiquitination (Figure 3P). Via homologically analyzing, we observed that K297 is conserved across different species, but different in other CDK family proteins (Figure 3Q). Interestingly, the 2KR mutant could decrease CDK4 stability as well (Figure S6L). In line with these findings, molecular docking results showed that compared with WT, 2KR and PE2A mutant could attenuate the interactive affinity of CDK4 with cyclin D1 (Figure 3R), which has been further validated by biochemical approach (Figure 3S), possibly resulting in decreased their cytoplasm colocalization (Figure 3S). These findings demonstrate that TRAF6‐mediated K11‐linked ubiquitination as a non‐degradative signal reinforcing CDK4‐cyclin D1 complex formation (Figure 3U).

### 2.6 ZDHHC17‐Mediated Palmitoylation Promotes CDK4 Ubiquitination

**FIGURE 4:**
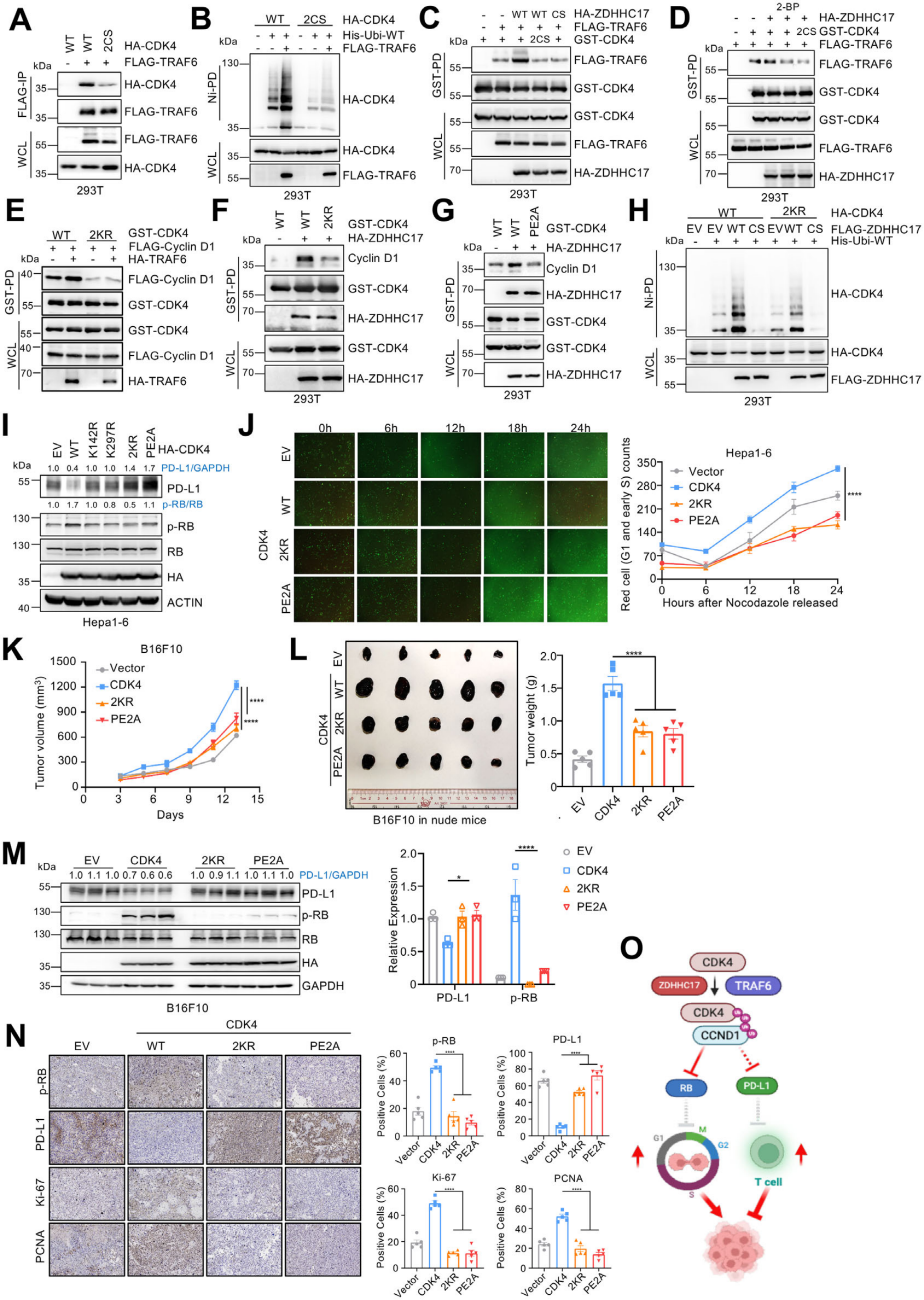
Palmitoylation regulates CDK4 ubiquitination. (A) WB analysis of FLAG pulldown product and WCL derived from HEK293T cells transfected with indicated constructs. (B) WB analysis of IP and WCL from Ni‐NTA pull‐down products derived from HEK293T cells transfected with indicated constructs. (C–G) WB analysis of GST pulldown product and WCL derived from HEK293T cells transfected with indicated constructs. (H) WB analysis of IP and WCL from Ni‐NTA pull‐down products derived from HEK293T cells transfected with indicated constructs. (I) WB analysis of WCL from Hepa1‐6 cells infected with indicated lentivirus and selected with hygromycin 5 days. (J) Cells generated in (H) were subjected for Fucci assays. Fluorescence imaging of Hepa1‐6 cells treated with Nocodazole (330 nm) for 12 h. The red cell numbers were counted and plotted (mean ± SEM, *n* = 3, two‐way ANOVA, ^****^ *p* < 0.0001). (K,L) B16F10 cell lines were subjected for xenografted mouse model, and the growth curves (K) and tumor weights (L) between different groups were analyzed (mean ± SEM, *n* = 5, two‐way ANOVA for growth curves; one‐way ANOVA for tumor weights, ^****^ *p* < 0.0001). (M,N) WB analysis of WCL derived from tumor tissues, and the relative protein levels were analyzed (mean ± SEM, *n* = 3, one‐way ANOVA, ^*^ *p* < 0.05, ^****^ *p* < 0.0001) (M). Representative images of immunohistochemistry (IHC) staining of paraffin section derived from tumor tissues, and the relative staining density were analyzed (mean ± SEM, *n* = 5, one‐way ANOVA, ^****^ *p* < 0.0001). Scale bar, 100 µm (N).

Given the potential role of ZDHHC17‐mediated CDK4 palmitoylation in enhancing TRAF6‐mediated CDK4 ubiquitination (Figure 3G–I), we examined the effect of palmitoylation on the ubiquitination of CDK4. The results showed that the palmitoylation‐deficient 2CS mutant disrupted the interaction between CDK4 and TRAF6 (Figure 4A and Figure S6M). Consistently, the 2CS mutation strongly inhibited TRAF6‐mediated CDK4 ubiquitination (Figure 4B and Figure S6N). Furthermore, the interdependence of palmitoylation and ubiquitination was further evidenced by the inability of ZDHHC17‐CS to enhance the interaction between TRAF6 and CDK4 (Figure 4C and Figure S6N), but this effect was abolished by 2‐BP treatment or CDK4 2CS mutation (Figure 4C,D). On the other hand, 2KR mutant blocked TRAF6‐elevated CDK4/Cyclin D1 interaction (Figure 4E), consistently, ZDHHC17 promoted the interaction between WT, but not 2KR mutant CDK4 with cyclin D1 (Figure 4F). Similarly, the PE2A mutant prevented ZDHHC17 enhancing the CDK4/cyclin D1 interaction (Figure 4G). While these mutants did not affect the interaction of ZDHHC17 with CDK4 (Figure S6O). Additionally, the 2KR mutant significantly reduced ZDHHC17‐mediated CDK4 ubiquitination (Figure 4H). Together, these findings suggest that ZDHH17‐mediated palmitoylation promotes CDK4 ubiquitination, sequentially facilitating CDK4/cyclin D1 interaction.

Next, we explored the functional role of CDK4 ubiquitination. To this end, we generated stable cell lines overexpressing CDK4‐K142R, ‐K297R, ‐2KR, and ‐PE2A mutants. The results showed that WT CDK4, but not the ubiquitination‐deficient or TRAF6‐binding‐deficient mutants, was able to elevate p‐RB and attenuate PD‐L1 expression in different cells (Figure 4I and Figure S7A), accompanied with cell cycle arrest (Figure 4J and Figure S7B). Biologically, WT, but not these CDK4 mutants enhanced cell proliferation detected by CCK‐8, EdU staining, and colony formation assays (Figure S7C–E). To further confirm the function of CDK4 ubiquitination, we employed an orthotopic tumor mouse model. In line with our in‐cell findings, in comparation with WT‐CDK4 expression, 2KR or PE2A CDK4 variants reduced tumor growth in vivo (Figure 4K,L), coupled with decreased p‐RB and proliferation marker Ki67 and cell cycle marker PCNA, increased PD‐L1 levels (Figure 4M,N). Similar results were also observed in Hepa1‐6‐derived liver orthotopic mouse model (Figure S7F,G). These observations imply that ZDHHC17‐mediated palmitoylation promotes CDK4 ubiquitination and subsequent oncogenic activity (Figure 4O).

### 2.7 Depletion of *Zdhhc17* Attenuates Cell Cycle in Vivo

**FIGURE 5:**
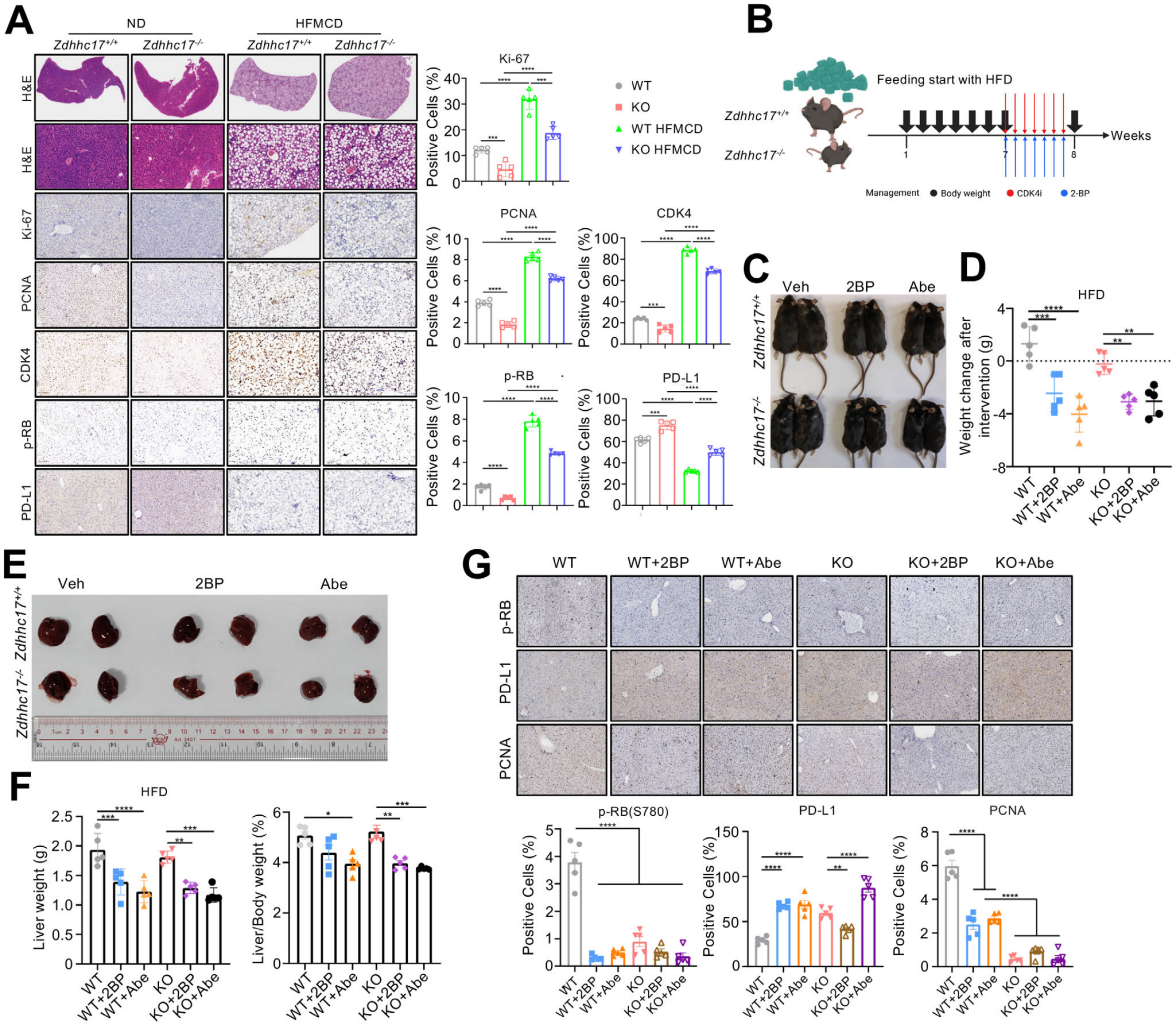
Depletion of *Zhdhc17* represses HCC models. (A) Representative image and IHC staining of livers harvested from *Zdhhc17^+/+^* or *Zdhhc17^−/−^* mice, which were subjected for HFMCD model. The relative staining of different markers was quantified (mean ± SEM, *n* = 5, one‐way ANOVA, ^***^ *p* < 0.001, ^****^ *p* < 0.0001). (B) Scheme of HFD feeding followed treatment of 2‐BP or CDK4 inhibitor in *Zdhhc17^+/+^* or *Zdhhc17^−/−^* mice. (C–F) Representative image of the mice (C), weight changes (D), liver images (E) from the mice as in (B). The relative liver weight and liver/body weight were quantified (mean ± SEM, *n* = 5, one‐way ANOVA, ^*^ *p* < 0.05, ^**^ *p* < 0.01, ^***^ *p* < 0.001, ^****^ *p* < 0.0001) (F). (G) IHC staining of liver tissues derived from (E), the staining was quantified (mean ± SEM, *n* = 5, one‐way ANOVA, ^**^ *p* < 0.01, ^****^ *p* < 0.0001).

To investigate the role of ZDHHC17 in cell cycle control in vivo, we employed *Zdhhc17*‐KO mice, and subjected them for high‐fat methionine‐choline‐deficient diet (HFMCD)‐mediated metabolic dysfunction‐associated steatohepatitis (MASH) mouse model [23]. The results showed that depletion of *Zdhhc17* largely attenuated hepatic cell proliferation (Ki67), cell cycle (PCNA), CDK4 levels, and its activity (p‐RB), while enhancing PD‐L1 expression (Figure 5A). In line with these findings, HFMCD‐feeding mice displayed higher levels of these markers with low PD‐L1 expression, which could readily be attenuated by *Zdhhc17* depletion (Figure 5A).

To validate the connection between ZDHHC17‐mediated palmitoylation and CDK4 activity in vivo, we treated mice with 2‐BP or CDK4 inhibitor (Abe) (Figure 5B). The results showed that *Zdhhc17* knockout reduced high‐fat diet (HFD)‐induced weight gain in mice, while both 2‐BP and Abe treatments significantly attenuated HFD‐induced weight gain [36], particularly in WT mice (Figure 5C,D and Figure S8A–C). Upon liver harvested, although both 2‐BP and Abe treatments were found to reduce the liver weight under HFD conditions, whereas *Zdhhc17* depletion further mitigated this effect (Figure 5E,F), but the ration of liver/body weight displayed a mild effect (Figure 5F). Importantly, *Zdhhc17* deficiency was associated with reduced DNA replication (as indicated by lower PCNA levels) and decreased CDK4 activity (reflected by reduced p‐RB levels), along with an increase in PD‐L1 expression (Figure 5G). In contrast, treatment with 2‐BP or Abe markedly suppressed p‐RB and PCNA levels and increased PD‐L1 expression in WT, but not in *Zdhhc17*‐KO mice (Figure 5G). These findings together suggest that ZDHHC17 plays a potential role in regulating cell cycle through CDK4 palmitoylation, at least in the context of HFD‐induced liver diseases.

### 2.8 High Expression of ZDHHC17 Indicates ICB Therapeutic Resistance

**FIGURE 6:**
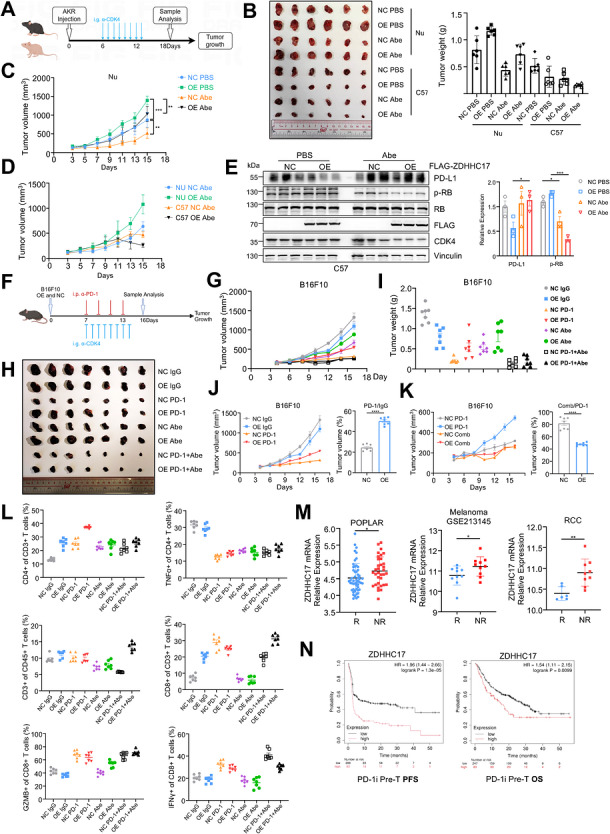
ZDHHC17 activates CDK4 to promote the balance of cell cycle and immune surveillance. (A) Scheme of implanted AKR overexpressed ZDHHC17 cells and drugs government in nude and C57BL/6 mice. (B–D) Tumor cells were subjected for xenografted mouse model and treated as illustrated (A), and the tumor weights (B) and growth curves (C,D) were analyzed in mice of (A) (mean ± SEM, *n* = 6, one‐way ANOVA for growth curves; one‐way ANOVA for tumor weights, ^**^ *p* < 0.01, ^***^ *p* < 0.001). (E) WB analysis of WCL derived from tumor tissues in C57 mice, and the relative protein levels were analyzed (mean ± SEM, *n* = 3, one‐way ANOVA, ^*^ *p* < 0.05, ^**^ *p* < 0.01, ^***^ *p* < 0.001, ^****^ *p* < 0.0001). (F‐I) B16F10 vector and OE‐ZDHHC17 implanted C57BL/6 mice were enrolled in different treatment groups as indicated (F). Tumor volumes of mice treated with control antibody, anti‐PD‐1 monoclonal antibody (mAb), Abemaciclib, or combined therapy were measured every two days and plotted individually. The growth curves (G) and tumor weights (H‐I) between different groups were analyzed (mean ± SEM, *n* = 7, two‐way ANOVA for growth curves; one‐way ANOVA for tumor weights). (J) The cell growth curve of 4 groups that mice received IgG and PD‐1 inhibitor (left panel). The shrinkage tumor volume under PD‐1 inhibitor government between vector and OE‐ZDHHC17 were calculated (right panel, mean ± SEM, *n* = 7, Student's *t*‐test, ^****^ *p* < 0.0001). (K) The cell growth curve of 4 groups that mice received PD‐1 inhibitor and combination treatment (left panel). The shrinkage tumor volume under combination/PD‐1 inhibitor treatment between vector and OE‐ZDHHC17 were calculated (right panel, mean ± SEM, *n* = 7, Student's *t*‐test, ^****^ *p* < 0.0001). (L) Flow cytometry staining analysis of tumor tissues. (M) The mRNA levels of ZDHHC17 in response (R) or non‐response (NR) for ICB treatment were analyzed in different Gene Expression Omnibus (GEO) and other databases, including HCC, lung, melanoma and RCC (Student's *t*‐test, ^*^ *p* < 0.05, ^**^ *p* < 0.01). (N) PFS and OS curves of patients who received anti‐PD‐1 treatment among all cancer types in Kaplan‐Meier Plotter database were analyzed based on ZDHHC17 expression before treating with anti‐PD‐1.

Given the dual role of CDK4 in cell cycle regulation and immune surveillance [37, 38], we investigated the impact of ZDHHC17 on tumor malignancy in vivo (Figure 6A). As expected, ZDHHC17 overexpression significantly drove CDK4‐dependent tumor growth in immunodeficient nude mice (Figure 6B), which was effectively suppressed by CDK4 inhibitors (Figure 6B,C). However, in the immunocompetent syngeneic mice, ZDHHC17 overexpression did not promote tumor growth in comparation to the counterpart mice (Figure 6B,D). Notably, enforced expression of ZDHHC17 led to an increased p‐RB levels and a decreased PD‐L1 expression, both of which were reversed by treatment with CDK4/6 inhibitors in both nude and C57BL/6 mice (Figure 6E and Figure S8D,E). These findings highlight the context‐dependent role of the ZDHHC17‐CDK4 axis in tumor progression under different immune conditions.

Given the potential oncogenic role of ZDHHC17 and its positive regulation on CDK4, we explored the therapeutic potential of combining CDK4/6 inhibitors with immune checkpoint blockade (ICB) in ZDHHC17‐OE tumors. To this end, we employed the well‐established B16‐based syngeneic mouse model (Figure 6F), and found that ZDHHC17 failed to promote tumor growth under this condition, possibly due to repressing PD‐L1 expression and restoring immune surveillance (Figure 6G–I). Consistent with this observation, anti‐PD‐1 treatment displayed less effective in suppressing tumors growth and weight in ZDHHC17‐OE group compared to their control counterparts (Figure 6J and Figure S8F–I). Notably, ZDHHC17‐OE tumors exhibited resistance to PD‐1 blockade alone but showed increased sensitivity to the combination of CDK4 inhibitor and anti‐PD‐1 therapy (Figure 6K). Consistent with these observations, the combination of CDK4 inhibitor with PD‐1 blockade not only arrested cancer cell cycle, but also blocked high‐PD‐L1‐mediated immune suppression (Figure 6L). Together, these preclinical studies suggest that tumors with high ZDHHC17 expression are more resistant to anti‐PD‐1 therapy alone but can be effectively targeted by using a combination of CDK4 inhibitors and immune checkpoint blockade.

At last, we aimed to assess the prevalence of ZDHHC17 expression and its association with immunotherapeutic response in cancer patients. To this end, we initially observed the correlation of ZDHHC17 with cell cycle in the human tumor samples (Figure S9A), and displayed higher expression in tumor compared to normal tissues, in particular in hepatocellular carcinoma (Figure S9B–H). Next, we analyzed multiple databases and observed a significant positive correlation between high ZDHHC17 expression and non‐response to ICB therapy across different cancer types, including non‐small‐cell lung cancer (POPLAR), melanoma (GSE213145), and renal cell carcinoma (RCC) (Figure 6M). Furthermore, elevated ZDHHC17 expression was associated with poorer overall survival (OS) and progression‐free survival (PFS) in patients undergoing anti‐PD‐1 treatment (Figure 6N). Collectively, these findings suggest that ZDHHC17 expression may serve as a biomarker for poor response to ICB therapy in cancer patients.

## 3 Discussion

**FIGURE 7:**
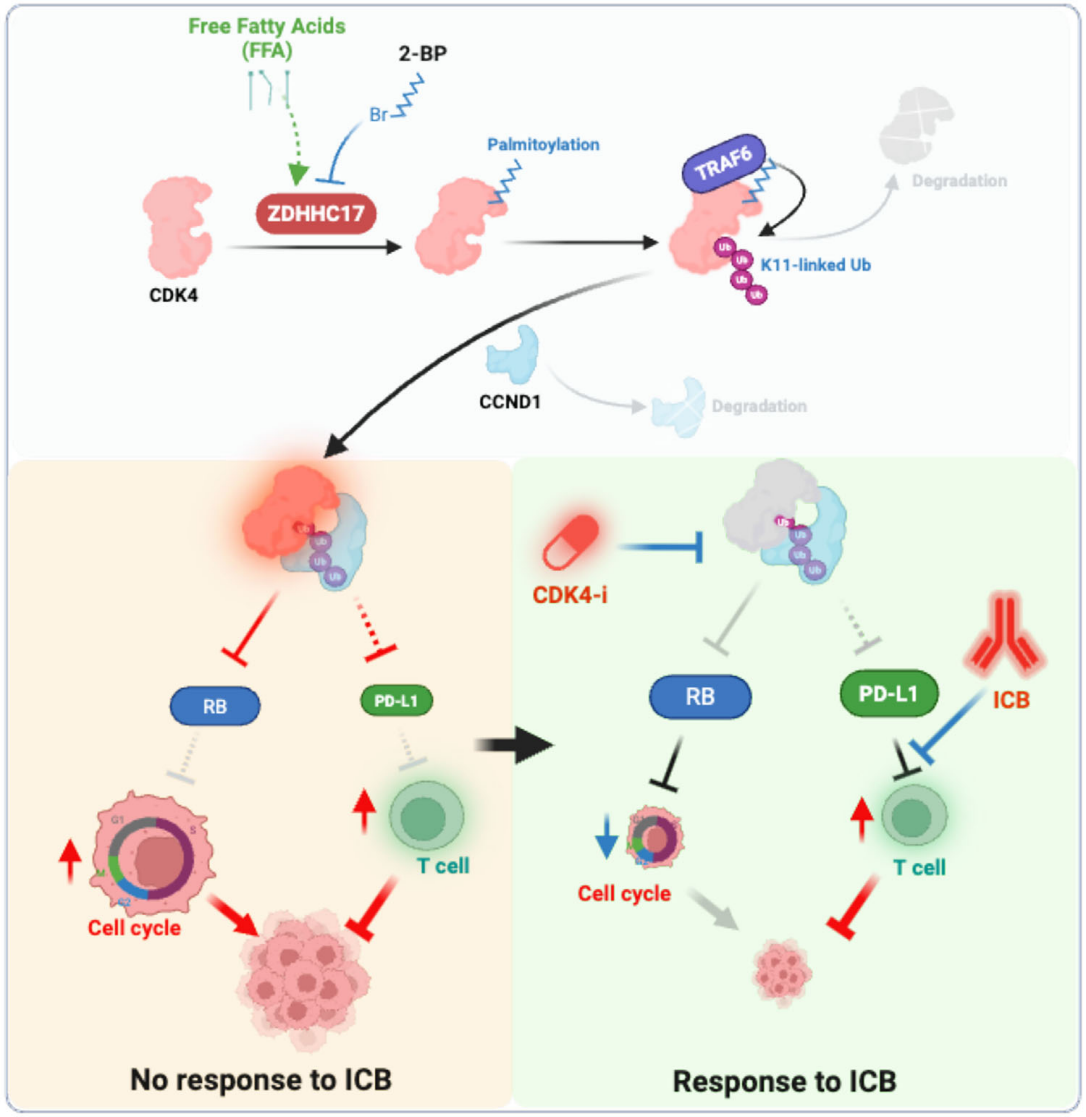
Model of ZDHHC17 in regulating CDK4 palmitoylation and cell cycle control. In top panel, the ZDHHC17‐mediated CDK4 palmitoylation could enhance TRAF6‐mediated CDK4 K11‐linked ubiquitination and its interaction with Cyclin D1, which promotes cell cycle by repressing RB, and boosts immune surveillant by repression of PD‐L1, contributing to tumor cell no response to ICB (left panel). While treatment with CDK4 inhibitors could block tumor cell cycle, and combination with ICB could repress PD‐L1, thus offering a new strategy for ZDHHC17‐driven cancers (right panel).

Although fatty acids are well known as major membrane components and energy sources for cell growth, their role in promoting cell cycle progression remains largely unexplored. Although 2‐BP is known as a non‐specific palmitoyl‐transferase inhibitor [23], our initial observation that 2‐BP blocks cell cycle progression suggests that this process may be regulated by ZDHHC‐mediated protein palmitoylation. Subsequently, we demonstrate that ZDHHC17‐mediated palmitoylation licenses CDK4 activation through subsequent stabilization of the kinase and its interaction with cyclin D1 to drive cell cycle progression (Figure 7). Furthermore, given the critical role of CDK4 in immune regulation, ZDHHC17 function is also tightly linked to immune responses by modulating PD‐L1 expression. These findings provide a rationale for combining CDK4 inhibitors with anti‐PD‐1 therapy for ZDHHC17‐high expression cancers (Figure 7).

Cell cycle is tightly regulated by cell phase dependent E3 ligases, such as CDC20, CDH1, and SKP2, via ubiquitinating and degrading cyclin proteins [39], thereby controlling CDKs kinase activity. However, apart from phosphorylation [40], the direct regulation of CDKs remains poorly defined. Here, we identify ZDHHC17‐mediated palmitoylation of CDK4 as a novel post‐translational modification. Rather than altering CDK4 subcellular localization, this modification regulates its stability and interaction with cyclin D1. Unlike canonical degradation related K48‐linked ubiquitination, this stabilization is pursued by TRAF6‐mediated K11‐linked ubiquitination. This atypical ubiquitination pathway not only reduces K48‐linked ubiquitination (which canonically leads to proteasomal degradation), but also facilitates CDK4‐cyclin D1 complex formation, integrated contributing to CDK4 kinase activity. Of note, a previous study reported that the C78A mutation does not affect CDK4 binding to cyclin D1 in pulmonary vascular cell setting [41], which may be explained by the use of a single mutation as opposed to our double C78/215 mutation. Thus, several key questions remain unclear and warrant further investigation: the precise mechanism by which palmitoylation affects CDK4 ubiquitination and its interaction with cyclin D1; how K11‐linked ubiquitination prevents K48‐linked degradation; and the identity of the E3 ligase responsible for K48‐linked ubiquitination of CDK4.

Given the critical role of CDK4 in driving cell cycle progression, the clinical relevance of CDK4 inhibition is underscored by its FDA approval in hormone receptor‐positive breast cancer [42]. However, therapeutic resistance to CDK4 inhibitors has emerged as a major challenge, driven by mechanisms such as compensatory CDK6 upregulation [43] or bypass signaling activation via alternative CDK4 substrates [44]. To overcome these resistance mechanisms, combination therapies involving CDK4 inhibitors and other targeted treatments have been developed, yielding significant clinical benefits [45]. In this study, we revealed that ZDHHC17‐mediated palmitoylation, followed by TRAF6‐mediated ubiquitination, activates CDK4, not only regulating its canonical role in cell cycle progression but also influencing tumor immune surveillance. As a result, tumors with high ZDHHC17 expression exhibit both aggressive cell cycle progression and a relatively immunosuppressive “cold” tumor microenvironment. This leads to a delicate balance in tumor growth under immunocompetent conditions, compared to the significantly enhanced tumor growth observed in immunodeficient settings (Figure 7). Consequently, ZDHHC17‐high expression tumors are characterized by immune‐cold conditions and resistance to immune checkpoint blockade (ICB) therapy. Given the recent advances in CDK4 inhibitor combination regimens, we propose that co‐targeting CDK4 and PD‐1/PD‐L1 could synergistically disrupt signaling in ZDHHC17‐driven tumors, as previously reported in breast cancer [46], could be an effective approach for treating these cancers. Of note, targeting ZDHHC17 itself may provide therapeutic benefits, while the lack of selective compounds. Our work provides a therapeutic blueprint for circumventing this limitation by exploiting the downstream convergence of ZDHHC17 signaling on CDK4 activation. Available CDK4 inhibitors can be repurposed to counteract both cell cycle dysregulation and immune evasion in ZDHHC17‐high tumors. However, future efforts should prioritize the development of specific inhibitors against ZDHHC17, which remains a significant challenge in the field.

In summary, we identify a novel mechanism by which fatty acids drive cell cycle progression through ZDHHC17‐mediated CDK4 palmitoylation, highlighting the potential of combining CDK4 inhibitors with ICB as a therapeutic strategy for ZDHHC17‐driven tumors.

## 4 Method and Materials

### 4.1 Plasmid Construction and Transfection

The coding sequence of ZDHHC17 (NCBI accession: NM_032383.4) and CDK4 (NCBI accession: NM_000075.4) was amplified with PrimerSTAR HS DNA Polymerase (Takara; Cat#R010A) using primers:

Z17: Forward: 5'‐GCATAGATCTATGCAGCGGGAGGAGG‐3' (BglII site),

Reverse: 5'‐GCATGTCGACCTACACAAGCTGGTACCCAGATC‐3' (SalI site);

CDK4: Forward: 5'‐GCATGGATCCATGGCTACCTCTCGATATGAG‐3' (BamHI site),

Reverse: 5'‐GCATGTCGACTCACTCCGGATTACCTTCATC‐3' (SalI site);

Purified PCR product (QIAquick PCR Purification Kit, Qiagen; Cat#28104) and pcDNA3.1(+) vector (Invitrogen; Cat#V79020) were digested with endonucleases at 37°C for 2 h. Ligation was performed using DNA Ligation Kit (Takara; Cat#6023) at room temperature (RT) for 30 min. Competent *E. coli* DH5α (Solarbio; Cat#C1100‐20) were transformed and plated on LB‐ampicillin (100 µg/mL) agar. Positive clones were verified by Sanger sequencing (Azenta). For transfection, cells at 70%–80% confluence received plasmid DNA using polyethylenimine (PEI, Polysciences; Cat#23966) at a 1:3 (µg:µL) ratio. For site‐directed mutagenesis plasmid construction, mutation‐specific primers were designed using web‐based tools Primer Spanner (https://ps.biocloud.org.cn/index.php). PCR product was digested with restriction enzyme DpnI‐HF at 37°C for 4 h and then transformed within *E. coli* DH5α for clone to verified.

### 4.2 Cell Culture and Stable Cell Line Generation

HEK293T cells and other Human and murine tumor cell lines were maintained in DMEM supplemented with 10% FBS (Gibco; Cat#10099141) and 1% penicillin‐streptomycin (HyClone; Cat#SV30010). Lentiviral particles were produced by co‐transfecting psPAX2 (Addgene; Cat#12260), pMD2.G (Addgene; Cat#12259), and transfer plasmids using PEI. Viral supernatant was collected 48 and 72 h post‐transfection, filtered (0.45 µm), and used to infect target cells with 1 µg/mL polybrene (Sigma; Cat#H9268). Stable clones were selected using 2 µg/mL puromycin or 200 µg/mL hygromycin B for 7 days. Gene overexpression/knockdown was confirmed by qRT‐PCR and Western blot. All stable cell lines were replaced with cryopreserved cell lines at 3 months of culture.

### 4.3 Western Blotting

Tissues were lysed in RIPA buffer (Beyotime; Cat#P0013B), and cells were lysed in EBC lysis (50 mm Tris pH 7.5, 120 mm NaCl, 1% NP‐40) buffer containing protease and phosphatase inhibitor cocktail for general use (Beyotime; Cat#P1045). Protein concentration was determined by BCA assay (ThermoFisher; Cat#23225). Equal amounts (30 µg) of protein were separated on 8% or 10% SDS‐PAGE gels and transferred to PVDF membranes (Millipore; Cat#IPVH00010). Membranes were blocked with 5% non‐fat milk (Biofroxx; Cat#1172GR500) in TBST (Tris‐buffered saline add 0.1% Tween‐20) for 1 h at RT, then incubated with primary antibodies overnight at 4°C. After washing three times with TBST, HRP‐conjugated secondary antibodies (1:5000, Proteintech; Cat#SA00001‐2) were incubated for 1 h at RT. Signals were developed with ECL Prime (ThermoFisher; Cat#34095, 32109) and quantified using ImageJ (NIH).

### 4.4 Co‐Immunoprecipitation (Co‐IP)

Cells were lysed in EBC buffer supplemented with protease/phosphatase inhibitors. Lysates were pre‐cleared with Protein A/G beads (Thermo Fisher; Cat#20421) for 1 h at 4°C. For each IP, 500 µg lysate was incubated with 2 µg primary antibody overnight at 4°C. Immune complexes were captured with Protein A/G beads (2 h, 4°C), washed three times with NETN buffer (20 mm Tris, pH 8.0, 150 mm NaCl, 1 mm EDTA, and 0.5% NP‐40). Input and IP samples were analyzed by Western blot.

### 4.5 CCK8 Cell Proliferation Assay

Cells were seeded in 96‐well plates at a density of 2 × 10^3^ cells/well (Corning, USA; Cat#3599) and allowed to adhere for 24 h in complete medium. Following treatment with specified concentrations of compounds or experimental interventions, cells were incubated for 24, 48, 72, or 96 h under standard culture conditions (37°C, 5% CO_2_). Subsequently, 10 µL CCK‐8 reagent (Dojindo Molecular Technologies; Cat#CK04) was added to each well and incubated for 2 h in the dark. Absorbance at 450 nm was measured using a microplate reader (ThermoFisher/Multiskanskyhigh). Triplicate wells were analyzed per condition.

### 4.6 Colony Formation Assay

Single‐cell suspensions were prepared using 0.25% trypsin‐EDTA (Gibco; Cat#25200072) and plated at 800 cells/well in 6‐well plates (NEST Biotechnology; Cat#703001). Cells were maintained for 10–14 days with medium changes every 48 h. Colonies were washed with PBS and fixed with 10% acetic acid/10% methanol for 2 h at RT, and then stained with 0.1% crystal violet (Beyotime; Cat#C0121) in PBS for 2 h. Next, the plates were washed and air‐dried, and the number of clones was calculated via ImageJ (NIH).

### 4.7 EdU Staining

Cells were pulsed with 50 µm EdU (Click‐iT EdU Alexa Fluor 488 Imaging Kit, Thermo Fisher; Cat#C10337) for 2 h prior to fixation with 4% paraformaldehyde (15 min, RT). Permeabilization was performed using 0.5% Triton X‐100 (Beyotime; Cat#ST795) in PBS for 15 min. Click‐iT reaction cocktail was prepared according to manufacturer instructions and incubated with samples for 30 min protected from light. Nuclei were counterstained with DAPI (Sigma‐Aldrich; Cat#D9542; 1 µg/mL, 5 min). Fluorescent images were acquired using an inverted fluorescence microscope (Olympus/IX83) with consistent exposure settings across groups. EdU positive cells were quantified using Qupath 0.4.1 from ≥ 3 random fields per sample.

### 4.8 Flow Cytometric Cell Cycle/Apoptosis Analysis

Cells were harvested, washed twice with ice‐cold PBS, and fixed in 75% ethanol at −20°C overnight. After washing twice with ice‐cold PBS, fixed cells were treated with propidium iodide (PI)/RNase Staining Buffer Solution (BD Biosciences; Cat#550825) at RT for 15 min in the dark. Cell cycle distribution was acquired and analyzed using a CytoFLEX flow cytometer (Beckman Coulter) with CytExpert software (v2.4). Data from 10 000 events per sample were processed using FlowJo software (v10.8.1). For apoptosis detection, cells were stained with Annexin V‐PE/7‐AAD (Vazyme; Cat#A213‐02) according to manufacturer protocols.

### 4.9 Flow Cytometric Surface Marker Detection

Single‐cell suspensions were prepared from mouse organ or tumor tissue using gentleMACS Dissociator (Miltenyi Biotec; Cat#130‐093‐235) and filtered through 70‐µm cell strainers (Falcon; Cat#352350). Red blood cells were lysed using ACK buffer (Gibco; Cat#A1049201; 5 min, RT).

Cells were washed twice with FACS buffer (PBS + 2% FBS + 1 mm EDTA) and resuspended at 1 × 10^6^ cells/mL. Cells were incubated with Fc block (BD Biosciences; Cat#553142; 1:100, 10 min, 4°C) to minimize nonspecific binding.

Surface staining was performed using fluorochrome‐conjugated antibodies (1:100 dilution, BioLegend) for 30 min at 4°C in the dark. Then cells were washed twice with FACS buffer and processed for intracellular staining. Cells were fixed in 4% paraformaldehyde (PFA; Biosharp; Cat#BL539A; 15 min, RT). After fixation, cells were washed twice with FACS buffer and resuspended in 100 µL of permeabilization buffer for 30 min at 4°C. Cells were incubated with fluorochrome‐conjugated antibodies against intracellular targets (1:100 dilution, BioLegend) for 30 min at 4°C in the dark. After that, cells were washed twice with permeabilization buffer, followed by one wash with FACS buffer. Data were acquired on a Full spectrum flow cytometer (Cytek Aurora) using its software. Compensation controls were performed using single‐stained samples, and fluorescence minus one (FMO) control were included for accurate gating. Data were analyzed using FlowJo (v10.8.1).

### 4.10 Immunofluorescence

Cells planted in a confocal dish and after intervention were fixed in 4% paraformaldehyde at RT for 15 min. After treating with 0.3% Triton X‐100 at RT for 15 min, cells were subjected to 5% BSA at RT for 60 min to reduce nonspecific binding. Primary antibodies were incubated overnight at 4°C, and Alexa Fluor‐conjugated antibodies (1:1000) for 1 h at RT. After washing three times, the cells were stained with DAPI for 5 min and analyzed using confocal microscopy (olympus/FV3000). Quantification of the fluorescent intensity was performed with ImageJ software.

### 4.11 Quantitative Real‐Time PCR (qRT‐PCR)

Total RNA was extracted with TRIzol (Invitrogen; Cat#15596026) and reverse‐transcribed using PrimeScript RT Master Mix (Takara; Cat#RR036A). qPCR was performed on a system (Applied Biosystems QuantStudio 5) with TB Green Premix Ex Taq II (Takara; Cat#RR820A). Primers (0.2 µm each) were designed using Primer‐BLAST (NCBI).

### 4.12 GST Pull‐Down Assay

GST‐tagged protein and other constructs were transfected in HEK293T cells. Cell lysates expressing GST‐protein were incubated with GST beads (2 h, 4°C). Beads were washed 3× with NETN and boiled with 2× laemmli buffer. pull‐down products and input were analyzed by immunoblotting.

### 4.13 Ubiquitination Assay (in Cell and in Vitro)

HEK293T cells were transfected with His‐Ub‐WT or His‐K11‐only and the indicated plasmids. 36 h post‐transfection if contained His‐Ub‐WT, cells were treated with 10 µm MG132 (carbobenzoxy‐Leu‐Leu‐leucinal) for 12 h. All cells were washed with PBS twice and lysed in buffer A (6 m guanidine‐HCl, 0.1 m Na_2_HPO_4_/NaH_2_PO_4_, and 10 mm imidazole (pH 8.0)), and then subjected to sonicate. The supernatants obtained after high‐speed centrifugation were incubated with Ni‐NTA nickel beads (Qiagen, Cat#30210) for 3 h at room temperature. The beads underwent sequential washes: twice with buffer A, twice with buffer A/TI (1:3 ratio of buffer A to buffer TI), and once with buffer TI (25 mm Tris–HCl, 20 mm imidazole, pH 6.8). The captured proteins were then separated by 8% SDS‐PAGE and analyzed by immunoblotting.

For in vitro ubiquitin assay, purified CDK4 was incubated with E1, E2 (UBE1 and UbcH5b), and ubiquitin (obtained from UBbiotech) in the reaction buffer at 37°C for 2 h. Reactions were resolved by SDS‐PAGE for immunoblotting.

### 4.14 Protein Purification

Recombinant GST‐ZDHHC17 WT, GST‐ZDHHC17 CS were generated by performing the BL21(DE3) *E. coli* (Cat#EC0114) strain with pGEX‐4T1‐ZDHHC17. The starter culture was diluted 1:100 into 1 L fresh LB medium and grown at 37°C until the OD600 reached 0.6‐0.8. Protein expression was induced by adding 0.5 mm IPTG (Solarbio; Cat#I8070), followed by incubation at 16°C for 16–18 h with shaking at 200 rpm. Cells were harvested by centrifugation at 4000 × g for 15 min at 4°C, and the pellet was resuspended in 20 mL lysis buffer (50 mm Tris‐HCl pH 8.0, 300 mm NaCl, 10 mm imidazole, 1 mm PMSF, and protease inhibitors and lysozyme 1 mg/mL). The suspension was incubated on ice for 30 min and sonicated (3× 30 s pulses, 50% amplitude, on ice). Following incubation of the supernatant with Glutathione‐sepharose slurry (Cytiva; Cat#17007501) for 2 h at 4°C, the beads were washed three times with PBS buffer. The bound proteins were eluted with 10 mm reduced glutathione (Sigma; Cat#G4251) in 50 mm Tris‐HCl, pH 8.0. Eluted proteins were analyzed via Coomassie blue staining and quantified against a bovine serum albumin (BSA) standard.

For recombinant His‐CDK4 was generated by transforming the BL21 (DE3) *E. coli* strain with pET‐28a‐CDK4 and by the same strategy as GST‐tagged proteins. The main difference was that the supernatant was incubated with Ni‐NTA nickel beads in binding buffer (50 mm NaH2PO4, 300 mm NaCl, 10 mm imidazole, pH 8.0) for 2 h at 4°C. The beads were washed five times with wash buffer (50 mm NaH_2_PO_4_, 300 mm NaCl, 20 mm imidazole, pH 8.0), and bound proteins were eluted with elution buffer (50 mm NaH_2_PO_4_, 300 mm NaCl, 250 mm imidazole, pH 8.0).

### 4.15 Protein Purification and In Vitro Kinase Assay

The cDNA encoding RB (S780) was cloned into the pGEX‐4T1 vector and transformed into Escherichia coli BL21(DE3). Protein expression was induced with 0.5 mm isopropyl β‐D‐1‐thiogalactopyranoside (IPTG) at 16°C for 16–20 h. Cells were harvested by centrifugation, resuspended in PBS, and lysed by sonication on ice. The lysate was centrifuged at 12 000 × g for 30 min at 4°C, and the supernatant was incubated with pre‐equilibrated Glutathione Sepharose 4B beads at 4°C for 2 h. The beads were washed three times with PBS, and the bound protein was eluted with 50 mm Tris‐HCl (pH 8.0) containing 10 mm reduced glutathione. The eluted fractions containing the target protein, as identified by SDS‐PAGE, were pooled, aliquoted, and stored at –80°C.

The kinase reaction was performed in a 30 µL volume containing 5× kinase reaction buffer (250 mm HEPES pH 7.5, 50 mm MgCl_2_, 5 mm DTT, 0.5 mm Na_3_VO_4_), 1 µg of substrate protein (GST or GST‐RB), appropriate amounts of purified kinases (immunoprecipitated Flag‐CDK4/2CS, HA‐Cyclin D1, and GST‐Z17), and ATP (final concentration 200 µm). The reaction mixture was incubated at 30°C for 30 min. The reaction was terminated by adding an equal volume of 2× SDS sample buffer containing 100 mm DTT, followed by boiling at 100°C for 5 min. Samples were resolved by SDS‐PAGE, transferred to PVDF membranes, and subjected to Western blotting using an anti‐phospho‐RB (S780) specific antibody.

### 4.16 Acyl‐Biotin Exchange (ABE) Palmitoylation Assay

ABE palmitoylation assays were performed as described previously [23]. Cells that transfected with the indicated plasmids were lysed with lysis buffer 1 (50 mm Tris‐HCl pH 7.5, 150 mm NaCl, 1 mm MgCl_2_, 1% NP‐40, and protease inhibitor). After high‐speed centrifuged, the supernatants rotary incubated with 50 mm N‐ethylmaleimide at 4°C for 1.5 h, then rotary incubated with tag‐beads or endogenous antibodies and strep‐beads at 4°C for 3 h. Then the beads washed four times with lysis buffer 1 and washed three times with lysis buffer 2 (50 mm Tris‐HCl pH 7.2, 150 mm NaCl, 1 mm MgCl_2_). The beads rotary incubated with lysis buffer 2 contained 1 m hydroxylamine (HAM) or not at room temperature for 1 h, then washed four times with buffer 2 and washed three times with buffer 3 (50 mm Tris‐HCl pH 7.2, 150 mm NaCl, 1 mm MgCl_2_), and continued rotary incubated with buffer 3 contained 2 µm biotin‐BMCC at 4°C for 1 h. Finally, proteins were washed three times and subjected to immunoblotting analysis.

For in vitro assays, purified proteins (His‐CDK4, His‐CDK4‐2CS, GST‐ZDHHC17, GST‐ZDHHC17‐CS) were obtained by the method described above. 2 µg CDK4 protein, 2 µg ZDHHC17 protein, 20 µm palmitoyl alkyne coenzyme A, and 50 ng/µL BSA were incubated with 1× buffer (1 m Tris‐HCl, pH 8.0, 0.1 mm EDTA, 10× buffer) at 30°C for 2 h. Then the reaction buffer was subjected to ABE palmitoylation assay.

### 4.17 Hematoxylin and Eosin (H&E) Staining and Immunohistochemistry (IHC)

Tissue sections were deparaffinized, rehydrated, and stained with hematoxylin (Sigma; Cat#H9627; 5 min) followed by eosin (Sigma; Cat# HT110116; 2 min). Slides were dehydrated, cleared in xylene, and mounted with Permount (Fisher Scientific; Cat#SP15‐100). Histopathological analysis was performed by a board‐certified pathologist blinded to experimental conditions.

Formalin‐fixed, paraffin‐embedded tissue sections (3 µm) were deparaffinized and rehydrated. Antigen retrieval was performed in citrate buffer (pH 6.0) using a pressure cooker (95°C, 15 min). Endogenous peroxidase activity was quenched with 3% H_2_O_2_ (10 min, RT). Sections were blocked with 5% BSA (Sigma; Cat#A7906) and incubated with primary antibody overnight at 4°C. Detection was performed using HRP‐conjugated secondary antibody and DAB substrate (Dako; Cat#K5007). Slides were counterstained with hematoxylin, dehydrated, and mounted. Images were acquired using a Kfbio/KF‐PRO‐020 system. Positive cells or area were calculated via QuPath (V0.4.1).

### 4.18 RNA‐seq, GO and KEGG Pathway Analysis

Total RNA was extracted from KYSE30 shZDHHC17 stable cell lines, utilizing TRIzol (Takara; Cat#9109) in accordance with the manufacturer's instructions. The quantity and purity of the extracted total RNA were assessed using a Bioanalyzer 2100 and the RNA 6000 Nano LabChip Kit (Agilent, CA, USA), ensuring a RIN number exceeding 7.0. Approximately 10 µg of total RNA, representative of a particular adipose tissue type, was employed to isolate Poly (A) mRNA utilizing magnetic beads with poly‐T oligonucleotides (Invitrogen). Following this purification step, the mRNA was fragmented into smaller segments through the application of divalent cations at elevated temperatures. Subsequently, the cleaved RNA fragments underwent reverse transcription to generate the final cDNA library, adhering to the established protocol outlined in the mRNA‐Seq sample preparation kit (Illumina, San Diego, USA), with the average insert size for the paired‐end libraries being 300 bp (± 50 bp). Finally, paired‐end sequencing was conducted on an Illumina sequencing platform.

Differentially expressed genes (DEGs) were identified using DESeq2 with a significance threshold of |log2 fold change| > 2 and adjusted *p*‐value < 0.05. GO enrichment analysis was performed using the clusterProfiler R package (v4.0) to annotate DEGs into three categories: biological process (BP), molecular function (MF), and cellular component (CC). KEGG pathway analysis was conducted to identify significantly enriched pathways associated with the DEGs. The Benjamini‐Hochberg method was used to adjust *p*‐values for multiple testing, and terms with adjusted *p*‐values < 0.05 were considered statistically significant. Visualization of enriched terms was performed using ggplot2 (v3.3.5).

### 4.19 Gene Set Enrichment Analysis (GSEA)

GSEA was performed using the Fgsea package, and gene sets were obtained from the KEGG Database. Pre‐ranked GSEA was conducted using log_2_ fold changes from RNA‐seq data. Permutations were set to 1000, and gene sets with a false discovery rate (FDR) < 0.25 and nominal *p*‐value < 0.05 were considered significantly enriched. Enrichment plots were generated to visualize the leading‐edge subsets of genes contributing to the enrichment score.

### 4.20 Animal Experiments

All animal experiments were approved by the Institutional Animal Care and Use Committee (IACUC) of Sun Yat‐sen University Laboratory Animal Center (SYSU‐IACUC‐2024‐002709). Every effort was made to minimize animal suffering and reduce the number of animals used. Male BALB/c nude mice and C57BL/6 mice (6–8 weeks old; GemPharmatech Co., Ltd) were housed under specific pathogen‐free (SPF) conditions with a 12 h light/dark cycle, controlled temperature (22 ± 2°C), and humidity (50% ± 10%). Mice were allowed to acclimate for one week prior to experiments.

For tumor implantation, B16F10 and AKR cells were harvested during the logarithmic growth phase, washed twice with PBS, and resuspended in serum‐free medium. Viable cells (B16F10: 5 × 10^5^ cells and AKR: 2.5 × 10^6^ cells in 100 µL) were subcutaneously injected into the right flank of each mouse using a 27‐gauge needle. Tumor growth was monitored every 2–3 days using calipers, and tumor volume was calculated using the formula: Volume (mm^3^) = (Length × Width^2^) / 2.

When tumors reached an average volume of 100–150 mm^3^, mice were randomly divided into treatment and control groups (*n* = 6–8 per group). CDK4/6 inhibitor (Abemaciclib) was subsequently dissolved in DMSO, PEG300, Tween80, and normal saline, administered via oral gavage at a dose of 60 mg/kg every day for 8 days for B16F10 or 40 mg/kg for AKR. The control group received an equivalent volume of vehicle. Body weights were measured weekly, and tumor volume were measured once every other day to assess toxicity and therapeutic efficacy. Mice were humanely euthanized if tumor volume exceeded 2000 mm^3^ for B16F10 or 1500 mm^3^ for AKR and if signs of distress were observed.

At the endpoint, tumors were excised, weighed, and divided for further analysis. One portion was fixed in 4% paraformaldehyde (Biosharp; Cat# BL539A) for 24 h, embedded in paraffin, and sectioned for IHC or H&E staining. Another portion was snap‐frozen in liquid nitrogen and stored at −80°C for RNA or protein extraction. And the remaining portion was immediately prepared for Flow Cytometric Surface Marker Detection.

### 4.21 HFD model of *Zdhhc17* Knock‐Out Mice

*Zdhhc17* knock‐out mice were used as we reported [23]. Mice were continuously fed a 60% high‐fat diet, with weekly replenishment and body weight monitoring. At the beginning of week 8, the control solvent, 2‐BP, and the CDK4/6 inhibitor (Abe) were administered, respectively. At the end of week 8, mouse body weights were measured, and specimens were collected. The specimens were used for subsequent analysis and experiments.

### 4.22 ZDHHC17 mRNA Expression in Datasets

The individual participant data, including ZDHHC17 expression and status after therapy (Response or nonresponse) from the phase II POPLAR trial (NCT01903993, N = 192) [47] were used in this study. Other public data were downloaded from Gene Expression Omnibus and The Cancer Genome Atlas databases [48]. And the cutoff value of ZDHHC17 mRNA expression was defined as the median.

### 4.23 Statistical Analysis

Data are presented as mean ± SEM from ≥ 3 independent experiments. Statistical significance was determined by two‐tailed Student's *t*‐test (two groups), one‐way ANOVA with Tukey's post‐hoc test (multiple groups), and two‐way ANOVA with Tukey's post‐hoc test (multiple factors) using GraphPad Prism 9.0. *p*‐values < 0.05 were considered significant.

## Author Contributions

This study was conceived and designed by J. Guo and C. Cheng. The methodology was developed by Z. Wang, P. Song, and P. Feng. Acquisition of data (provided animals, acquired and managed patients, provided facilities, etc.) was carried out by Z. Wang, P. Song, X. Wu, L. Wang, and W. Xie. Analysis and interpretation of data (e.g., statistical analysis, biostatistics, computational analysis) was performed by Z. Wang, P. Song, X. Wu, L. Wang, and W. Xie. The manuscript was written by J. Guo, Z. Wang, and P. Song. Administrative, technical, or material support (i.e., reporting or organizing data, constructing databases) was provided by P. Feng, X. Wu, L. Wang, W. Xie, and C. Cheng. Study supervision was conducted by J. Guo, C. Cheng, and P. Feng. All authors approved the final manuscript.

## Ethics Statement

All animal experiments were approved by the Institutional Animal Care and Use Committee (IACUC) of Sun Yat‐sen University Laboratory Animal Center (No. SYSU‐IACUC‐2024‐002709).

## Consent

No written consent has been obtained from the patients as there is no patient identifiable data included in this case report/series.

## Conflicts of Interest

The authors declare no conflicts of interest.

## Supporting information




**Supporting File 1**: advs75693‐sup‐0001‐SuppMat.pdf.

**Supporting File 2**: advs75693‐sup‐0002‐Data.zip.

## Data Availability

The data that support the findings of this study are available on request from the corresponding author. The data are not publicly available due to privacy or ethical restrictions.
