# Supplementary material for: ZDHHC17‐Mediated CDK4 Palmitoylation Drives Cell Cycle Progression and Orchestrates Cancer Immune Surveillance
Source: Adv Sci (Weinh). 2026 May 14;13(44):e75693. doi: 10.1002/advs.75693 (PMC13335960; doi:10.1002/advs.75693)
Supplement: Supplementary file 1 [file ADVS-13-e75693-s001.pdf]

Supporting information for

CDK4 Palmitoylation Drives Tumor Cell Cycle Progression

Zekang Wang, Peipei Song, Xueji Wu, Lei Wang, Wei Xie, Pinning Feng, Chao Cheng, Jianping Guo

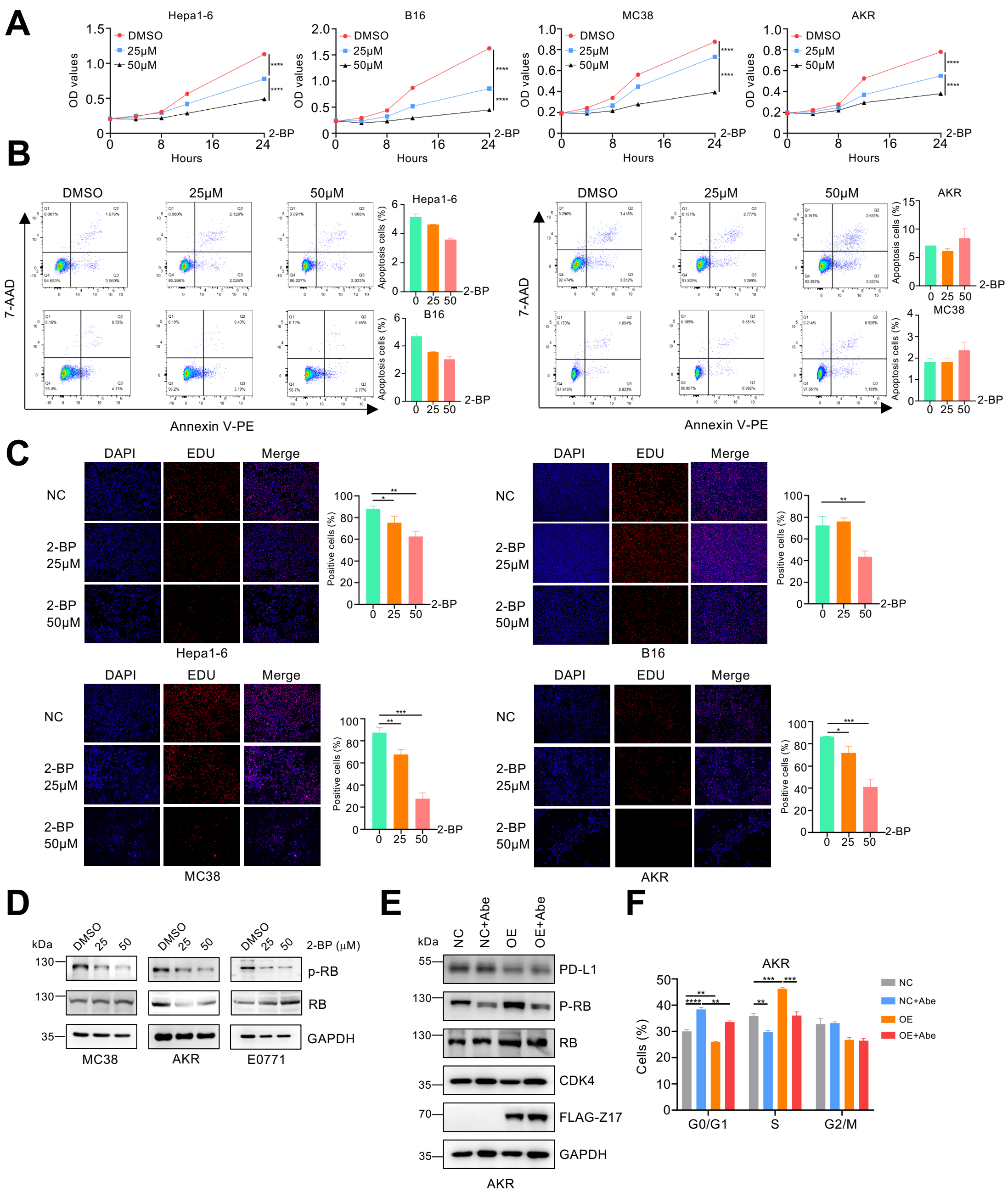

Figure S1. 2-BP represses cell proliferation and cell cycle via ZDHHC17. The CCK-8 assays of **(A)** Hepa1-6, B16F10, MC38, and AKR cells treated with 2-BP (0, 25, 50 μM) for indicated hours (mean ± SEM, n=3, two-way ANOVA, ****$P < 0.0001$). **(B)** Apoptosis analysis of Hepa1-6, B16F10, MC38, and AKR cells treated with 2-BP (0, 25, 50 μM) for 12 hrs via Annexin V-PE/7AAD assays (mean ± SEM, n=3one-way). **(C)** Representative images of EDU staining of Hepa1-6, B16F10, MC38, and AKR cells treated with 2-BP (0, 25, 50 μM) for 12 hrs, and the positive cells were analyzed (mean ± SEM, n=3, one-way ANOVA, *$P < 0.05$, **$P < 0.01$, ***$P < 0.001$, ****$P < 0.0001$). **(D)** WB analysis of WCL derived from MC38, AKR and E0771 cells treated with 2-BP (0, 25, 50 μM) for 12 hrs. **(E-F)** WB and Flow cell cycle analysis derived from AKR cells infected with vector or ZDHHC17 lentivirus treated with or without Abemaciclib.

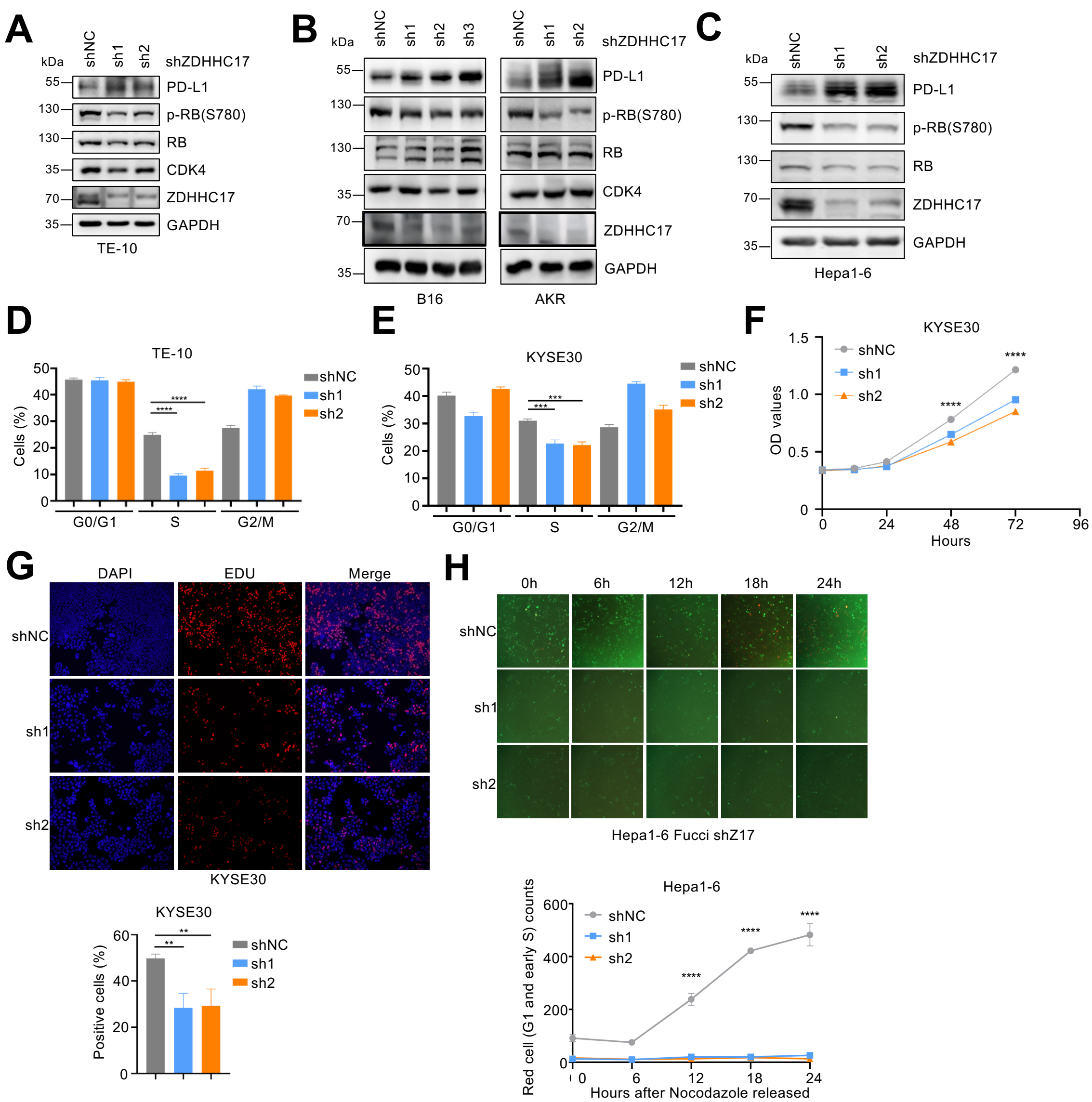

Figure S2. Knock-down *ZDHHC17* suppresses cell proliferation and cell cycle. (A) WB analysis of WCL derived from TE-10 cells infected with shZDHHC17 lentivirus. **(B)** WB analysis of WCL derived from B16F10 and AKR cells infected with shZDHHC17 lentivirus. **(C)** WB analysis of WCL derived from Hepa1-6 cells infected with shZDHHC17 lentivirus. **(D-E)** Flow cytometry analysis of cell cycle in TE-10 and KYSE30 cells infected with shZDHHC17 lentivirus (mean ± SEM, n=3, one-way ANOVA, ****$P < 0.0001$). **(F)** The CCK-8 assays of KYSE30 cells infected with shZDHHC17 lentivirus (mean ± SEM, n=3, two-way ANOVA, ****$P < 0.0001$). **(G)** Representative images of EDU staining of KYSE30 cells infected with shZDHHC17 lentivirus (mean ± SEM, n=3, one-way ANOVA, **$P < 0.01$). **(H)** Fluorescence imaging of Fucci infected Hepa1-6 shZDHHC17 cells treated with Nocodazole (330 nM) for 12 hrs (mean ± SEM, n=3, two-way ANOVA, ****$P < 0.0001$).

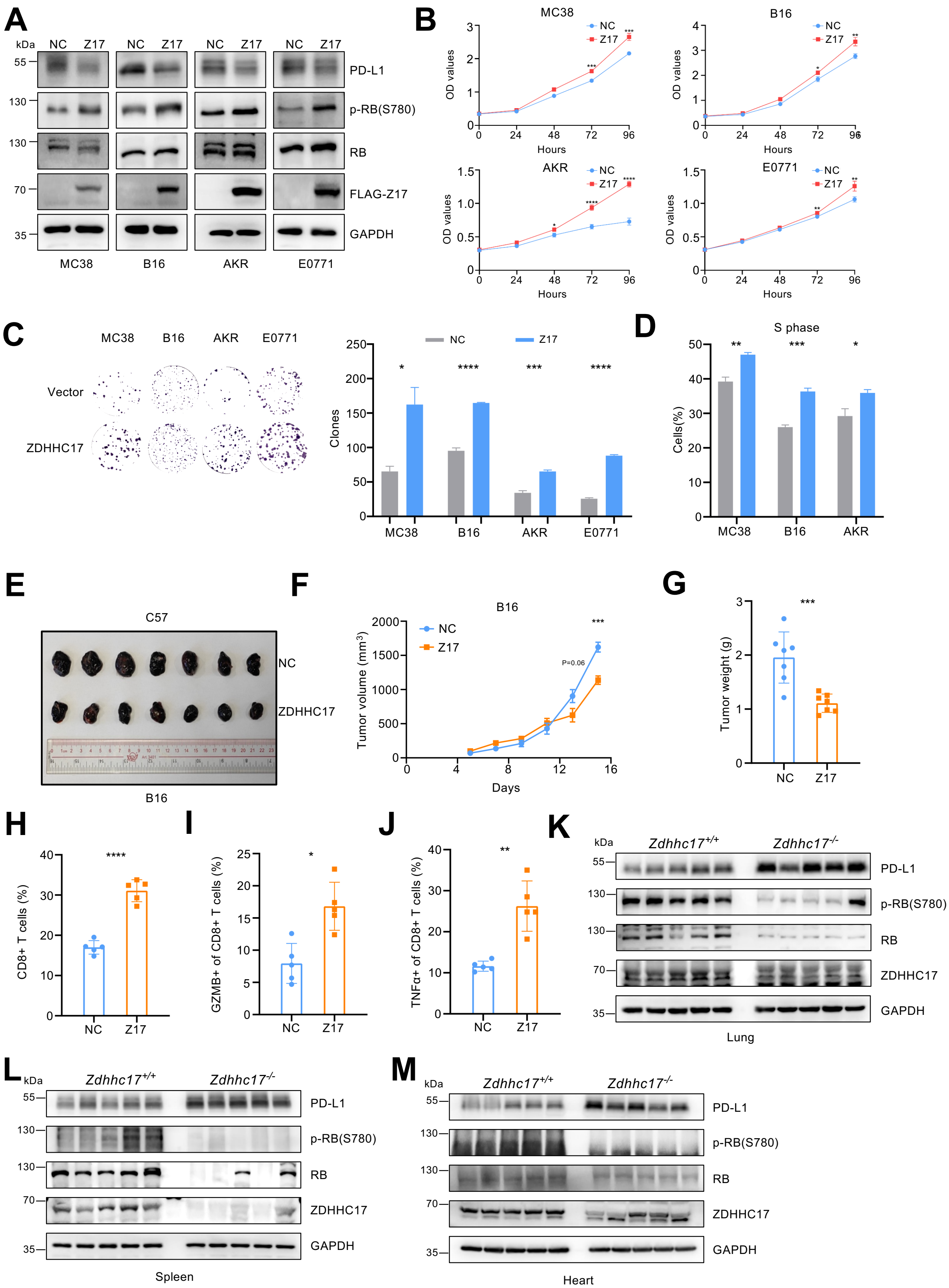
A
kDa
NC
Z17
PD-L1
p-RB(S780)
RB
FLAG-Z17
GAPDH
MC38
B16
AKR
E0771
B
OD values
Hours
C
Vector
ZDHHC17
Clones
D
S phase
Cells(%)
E
C57
NC
ZDHHC17
B16
F
Tumor volume (mm³)
Days
P=0.06
G
Tumor weight (g)
H
CD8+ T cells (%)
I
GZMB+ of CD8+ T cells (%)
J
TNFα+ of CD8+ T cells (%)
K
Zdhhc17+/+
Zdhhc17-/-
ZDHHC17
Lung
L
Spleen
M
Heart

Figure S3. ZDHHC17 promotes cell proliferation and cell cycle. (A) WB analysis of WCL derived from MC38, B16F10, AKR and E0771 cells infected with FLAG-ZDHHC17 lentivirus. **(B)** CCK-8 assays of MC38, B16F10, AKR and E0771 cells infected with FLAG-ZDHHC17 lentivirus (mean ± SEM, n=3, two-way ANOVA, *$P < 0.05$, **$P < 0.01$, ***$P < 0.001$, ****$P < 0.0001$). **(C)** Representative images of colony formation assays of MC38, B16F10, AKR and E0771 cells infected with FLAG-ZDHHC17 lentivirus (mean ± SEM, n=3, Student' *t* test, *$P < 0.05$, **$P < 0.01$, ***$P < 0.001$, ****$P < 0.0001$). **(D)** Flow cytometry analysis of cell cycle in MC38, B16F10, and AKR cells infected with FLAG-ZDHHC17 lentivirus (mean ± SEM, n=3, Student' *t* test, ****$P < 0.0001$). **(E-G)** B16F10 overexpressed FLAG-ZDHHC17 cells were subjected for xenografted mouse model without treatment, and the tumor image was presented (E), growth curves (F) and weights (G) were analyzed (mean ± SEM, n=7, two-way ANOVA for growth curves; Student's *t* test for tumor weights, **$P < 0.01$, ***$P < 0.001$). **(H-J)** Flow cytometry of tumor tissues derived from (E), the staining was quantified (mean ± SEM, n=5, Student's *t* test, **$P < 0.01$, ****$P < 0.0001$). **(K-M)** WB analysis of WCL derived from lung **(K)**, spleen **(L)** and heart **(M)** tissues of *Zdhhc17*-KO ($Zdhhc17^{-/-}$) and counterpart mice.

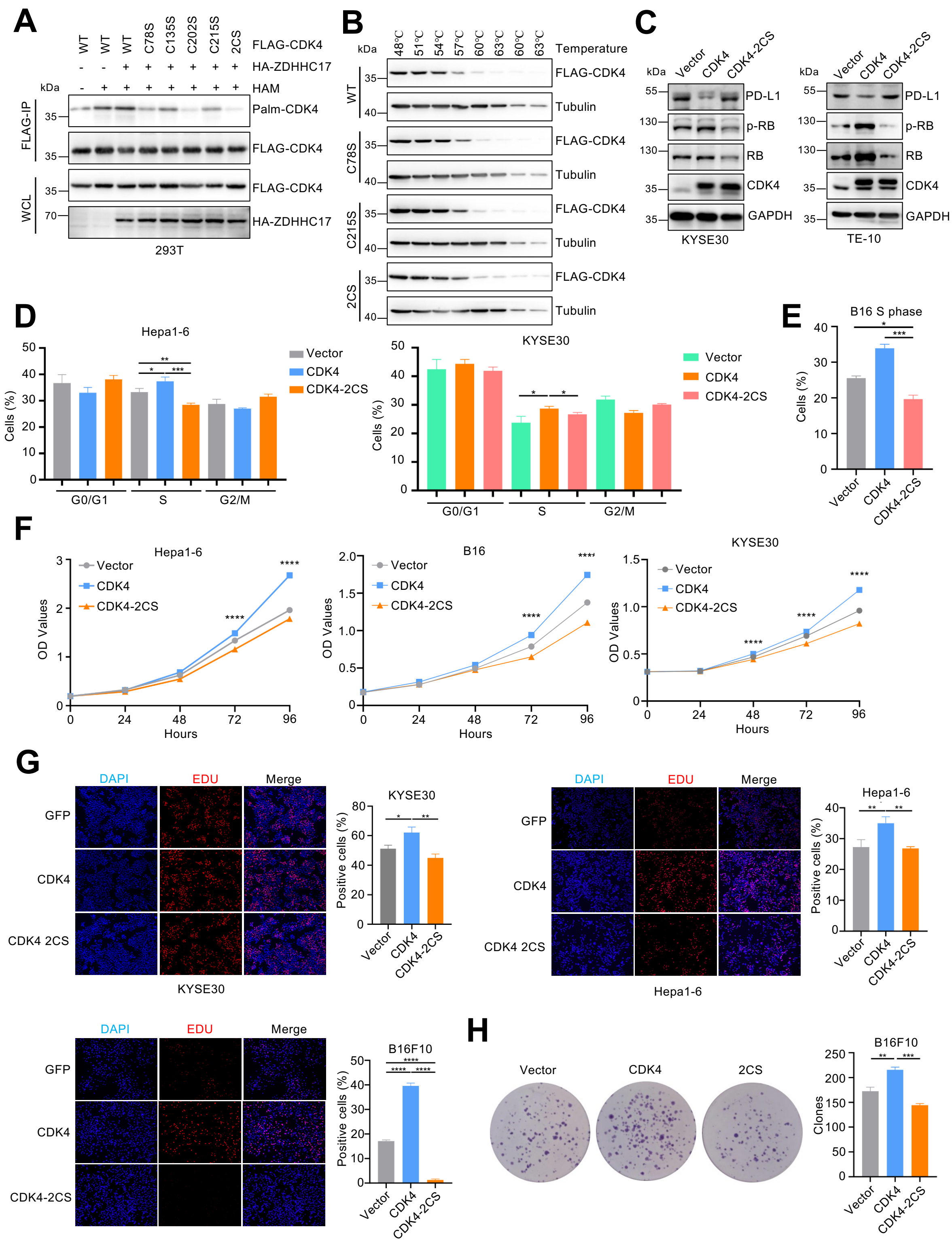
A
WT WT WT C78S C135S C202S C215S 2CS FLAG-CDK4
HA-ZDHHC17
HAM
kDa
FLAG-IP
WCL
Palm-CDK4
FLAG-CDK4
FLAG-CDK4
HA-ZDHHC17
293T
B
48℃ 51℃ 54℃ 57℃ 60℃ 63℃ 60℃ 63℃ Temperature
WT
C78S
C215S
2CS
FLAG-CDK4
Tubulin
C
Vector CDK4 CDK4-2CS
PD-L1
p-RB
RB
CDK4
GAPDH
KYSE30
TE-10
D
Hepa1-6
KYSE30
Vector
CDK4
CDK4-2CS
Cells (%)
G0/G1
S
G2/M
E
B16 S phase
Cells (%)
F
Hepa1-6
B16
KYSE30
OD Values
Hours
G
DAPI
EDU
Merge
GFP
CDK4
CDK4 2CS
CDK4-2CS
KYSE30
Hepa1-6
B16F10
Positive cells (%)
H
Vector
CDK4
2CS
Clones

Figure S4. CDK4-2CS suppresses cell proliferation. (A) WB analysis of IP and WCL from FLAG pull-down products derived from HEK293T cells transfected with indicated constructs. **(B)** WB Analysis of Protein Thermal Stability from HEK293T cells transfected with indicated constructs. **(C)** WB analysis of WCL derived from KYSE30 and TE-10 cells infected with HA-CDK4 and HA-CDK4-2CS lentivirus. **(D-E)** Flow cytometry analysis of cell cycle in Hepa1-6, B16F10and KYSE30 cells infected with HA-CDK4 and HA-CDK4-2CS lentivirus (mean ± SEM, n=3, one-way ANOVA, *$P < 0.05$, **$P < 0.01$, ***$P < 0.001$, ****$P < 0.0001$). **(F)** CCK-8 assays of Hepa1-6, B16F10and KYSE30 cells infected with HA-CDK4 and HA-CDK4-2CS lentivirus (mean ± SEM, n=3, two-way ANOVA, *$P < 0.05$, **$P < 0.01$, ***$P < 0.001$, ****$P < 0.0001$). **(G)** Representative images of EDU staining of KYSE30, Hepa1-6 and B16F10 cells infected with HA-CDK4 and HA-CDK4-2CS lentivirus (mean ± SEM, n=3, one-way ANOVA, *$P < 0.05$, **$P < 0.01$, ***$P < 0.001$, ****$P < 0.0001$). **(H)** Representative images of colony formation assays of B16F10 cells infected with HA-CDK4 and HA-CDK4-2CS lentivirus (mean ± SEM, n=3, one-way ANOVA, **$P < 0.01$, ***$P < 0.001$).

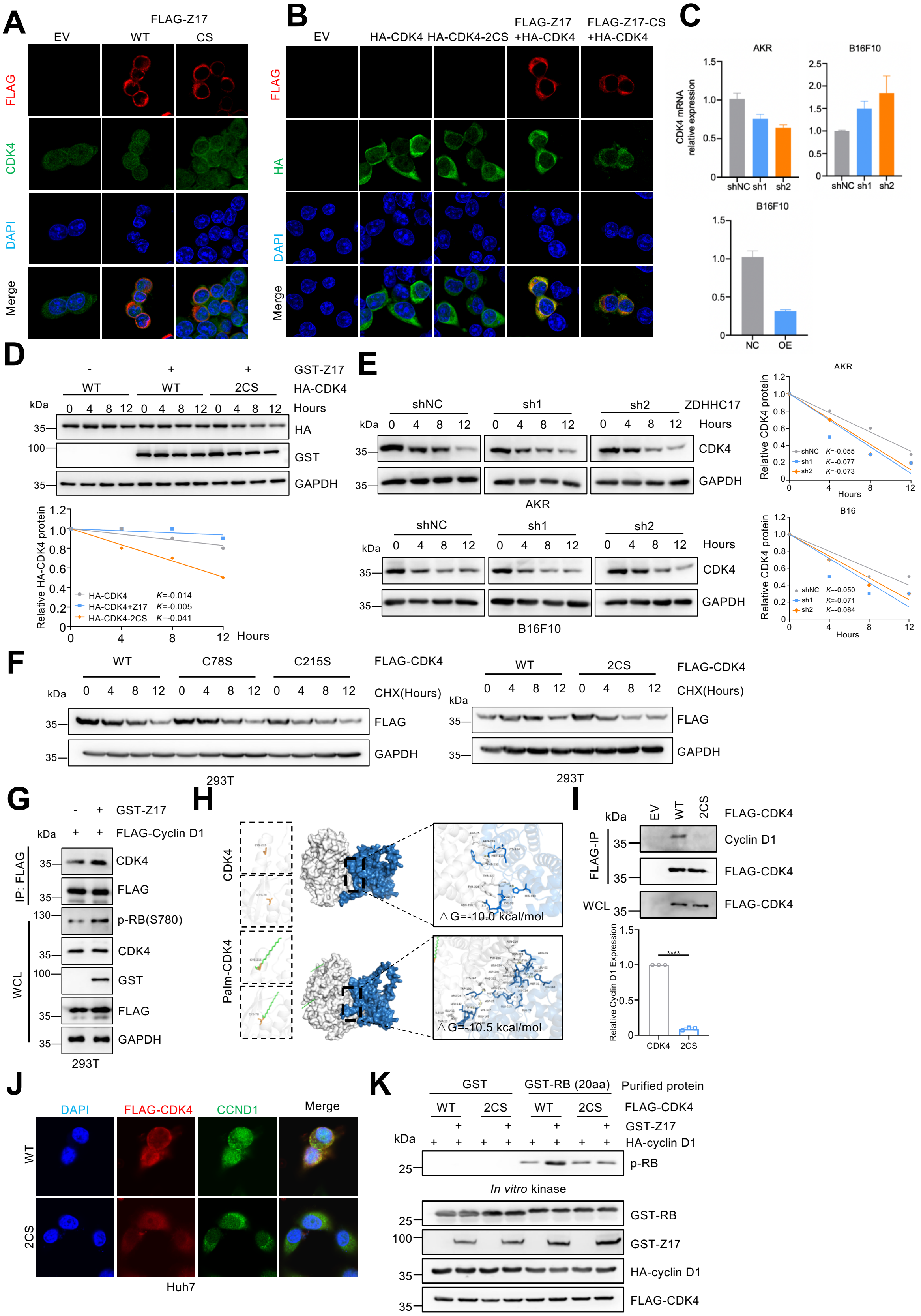
A
FLAG-Z17
EV
WT
CS
FLAG
CDK4
DAPI
Merge
B
EV
HA-CDK4
HA-CDK4-2CS
FLAG-Z17 +HA-CDK4
FLAG-Z17-CS +HA-CDK4
FLAG
HA
DAPI
Merge
C
AKR
B16F10
CDK4 mRNA relative expression
shNC
sh1
sh2
NC
OE
D
GST-Z17
HA-CDK4
WT
2CS
Hours
kDa
HA
GST
GAPDH
Relative HA-CDK4 protein
HA-CDK4 K=-0.014
HA-CDK4+Z17 K=-0.005
HA-CDK4-2CS K=-0.041
Hours
E
shNC
sh1
sh2
ZDHHC17
Hours
CDK4
GAPDH
AKR
B16F10
Relative CDK4 protein
shNC K=-0.055
sh1 K=-0.077
sh2 K=-0.073
B16
shNC K=-0.050
sh1 K=-0.071
sh2 K=-0.064
F
WT
C78S
C215S
FLAG-CDK4
CHX(Hours)
FLAG
GAPDH
293T
G
GST-Z17
FLAG-Cyclin D1
IP: FLAG
WCL
CDK4
FLAG
p-RB(S780)
GST
GAPDH
293T
H
CDK4
Palm-CDK4
△G=-10.0 kcal/mol
△G=-10.5 kcal/mol
I
EV
WT
2CS
FLAG-CDK4
FLAG-IP
Cyclin D1
FLAG-CDK4
WCL
Relative Cyclin D1 Expression
****
CDK4
2CS
J
DAPI
FLAG-CDK4
CCND1
Merge
WT
2CS
Huh7
K
GST
GST-RB (20aa)
Purified protein
WT
2CS
FLAG-CDK4
GST-Z17
HA-cyclin D1
p-RB
In vitro kinase
GST-RB
GST-Z17
HA-cyclin D1
FLAG-CDK4

Figure S5. ZDHHC17 potentially promotes CDK4 stability. (A-B) Representative images of immunofluorescence staining of HEK293T cells transfected with indicated constructs. **(C)** qRT-PCR analysis of CDK4 mRNA relative expression from AKR and B16F10 shZDHHC17 and B16F10 overexpressed ZDHHC17 cell lines. **(D)** WB analysis of WCL derived from HEK293T cells transfected with indicated constructs employed by CHX assays, decay curves were quantified. **(E)** WB analysis of WCL derived from B16F10 and AKR cells infected with shZDHHC17 lentivirus employed by CHX assays, decay curves were quantified. **(F)** WB analysis of WCL derived from HEK293T cells transfected with indicated constructs employed by CHX assays**. (G)** WB analysis of WCL and IP products derived from HEK293T cells transfected with indicated constructs. (**H**) Docking of the interaction between CDK4 and CCND1 with HDOCK. The binding affinity was predicted by PRODIGY. (**I**) WB analysis of WCL derived from HEK293T cells transfected with indicated constructed. The relative protein levels were quantified (mean ± SEM, n=3, Student's *t* test, $^{**}P < 0.01$, $^{***}P < 0.001$). (**J**) Huh7 cells were infected with different constructs and selected with puromycin for 72 hrs, then subjected to IF analysis. (**K**) *In vitro* kinase assays were performed with bacterially purified GST-RB (21aa, containing S780 site) as substrate, and the purified CDK4/cyclin D1 from 293T cell transfected with indicated constructs were used as kinase.

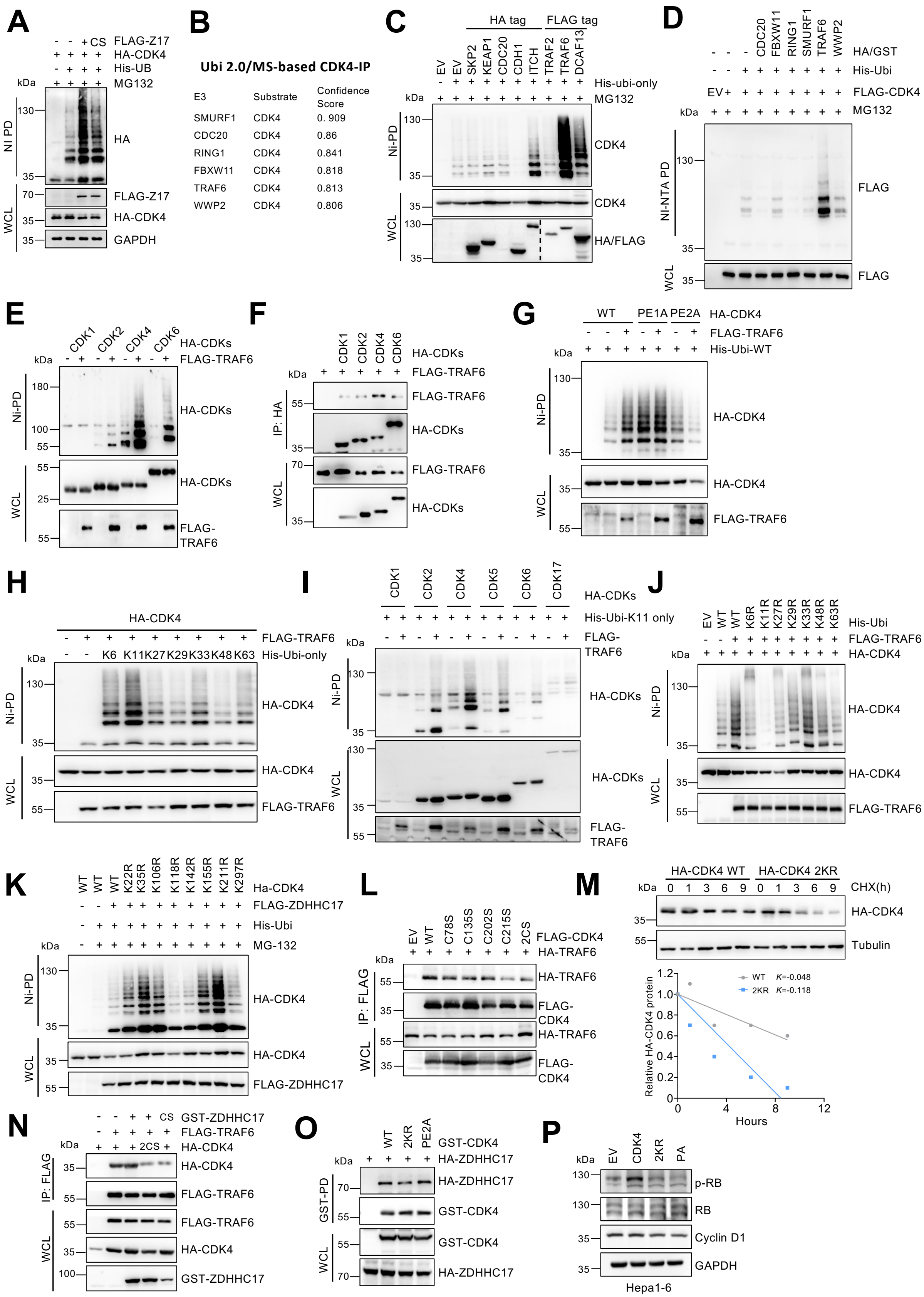
A
FLAG-Z17
HA-CDK4
His-UB
MG132
NI PD
HA
WCL
FLAG-Z17
HA-CDK4
GAPDH
B
Ubi 2.0/MS-based CDK4-IP
E3 Substrate Confidence Score
SMURF1 CDK4 0. 909
CDC20 CDK4 0.86
RING1 CDK4 0.841
FBXW11 CDK4 0.818
TRAF6 CDK4 0.813
WWP2 CDK4 0.806
C
HA tag
FLAG tag
EV EV SKP2 KEAP1 CDC20 CDH1 ITCH TRAF2 TRAF6 DCAF13
His-ubi-only
MG132
Ni-PD
CDK4
WCL
CDK4
HA/FLAG
D
EV CDC20 FBXW11 RING1 SMURF1 TRAF6 WWP2
HA/GST
His-Ubi
FLAG-CDK4
MG132
NI-NTA PD
FLAG
WCL
FLAG
E
CDK1 CDK2 CDK4 CDK6
HA-CDKs
FLAG-TRAF6
Ni-PD
HA-CDKs
WCL
HA-CDKs
FLAG-TRAF6
F
CDK1 CDK2 CDK4 CDK6
HA-CDKs
FLAG-TRAF6
IP: HA
FLAG-TRAF6
HA-CDKs
WCL
FLAG-TRAF6
HA-CDKs
G
WT PE1A PE2A
HA-CDK4
FLAG-TRAF6
His-Ubi-WT
Ni-PD
HA-CDK4
WCL
HA-CDK4
FLAG-TRAF6
H
HA-CDK4
FLAG-TRAF6
K6 K11 K27 K29 K33 K48 K63
His-Ubi-only
Ni-PD
HA-CDK4
WCL
HA-CDK4
FLAG-TRAF6
I
CDK1 CDK2 CDK4 CDK5 CDK6 CDK17
HA-CDKs
His-Ubi-K11 only
FLAG-TRAF6
Ni-PD
HA-CDKs
WCL
HA-CDKs
FLAG-TRAF6
J
EV WT WT K6R K11R K27R K29R K33R K48R K63R
His-Ubi
FLAG-TRAF6
HA-CDK4
Ni-PD
HA-CDK4
WCL
HA-CDK4
FLAG-TRAF6
K
WT WT WT K22R K35R K106R K118R K142R K155R K211R K297R
Ha-CDK4
FLAG-ZDHHC17
His-Ubi
MG-132
Ni-PD
HA-CDK4
WCL
HA-CDK4
FLAG-ZDHHC17
L
EV WT C78S C135S C202S C215S 2CS
FLAG-CDK4
HA-TRAF6
IP: FLAG
HA-TRAF6
FLAG-CDK4
WCL
HA-TRAF6
FLAG-CDK4
M
HA-CDK4 WT
HA-CDK4 2KR
CHX(h)
0 1 3 6 9 0 1 3 6 9
HA-CDK4
Tubulin
WT K=-0.048
2KR K=-0.118
Relative HA-CDK4 protein
Hours
N
GST-ZDHHC17
FLAG-TRAF6
HA-CDK4
IP: FLAG
HA-CDK4
FLAG-TRAF6
WCL
FLAG-TRAF6
HA-CDK4
GST-ZDHHC17
O
WT 2KR PE2A
GST-CDK4
HA-ZDHHC17
GST-PD
HA-ZDHHC17
GST-CDK4
WCL
GST-CDK4
HA-ZDHHC17
P
EV CDK4 2KR PA
p-RB
RB
Cyclin D1
GAPDH
Hepa1-6

Figure S6. TRAF6 interacts with and ubiquitinates CDK4. (A) WB analysis of IP and WCL from Ni-NTA pull-down products derived from HEK293T cells transfected with indicated constructs and treated with MG132 (10 μM) for 12 hrs. **(B)** Potential E3 enzymes interacting with CDK4 were overlapped proteins predicted by UbiBrowser2.0 database and CDK4-MS results. **(C-E)** WB analysis of IP and WCL from Ni-NTA pull-down products derived from HEK293T cells transfected with indicated constructs and treated with MG132 (10 μM) for 12 hrs. **(F)** WB analysis of IP and WCL derived from HEK293T cells transfected with indicated constructs. **(G-K)** WB analysis of IP and WCL from Ni-NTA pull-down products derived from HEK293T cells transfected with indicated constructs and treated with MG132 (10 μM) for 12 hrs. (**L**) WB analysis of WCL derived from HEK293T cells transfected with indicated constructs employed by CHX assays, decay curves were quantified. **(M)** WB analysis of IP and WCL derived from HEK293T cells transfected with indicated constructs. **(N-O)** WB analysis of IP and WCL derived from HEK293T cells transfected with indicated constructs. **(P)** WB analysis of WCL derived from Hepa1-6 cells infected with indicated lentivirus.

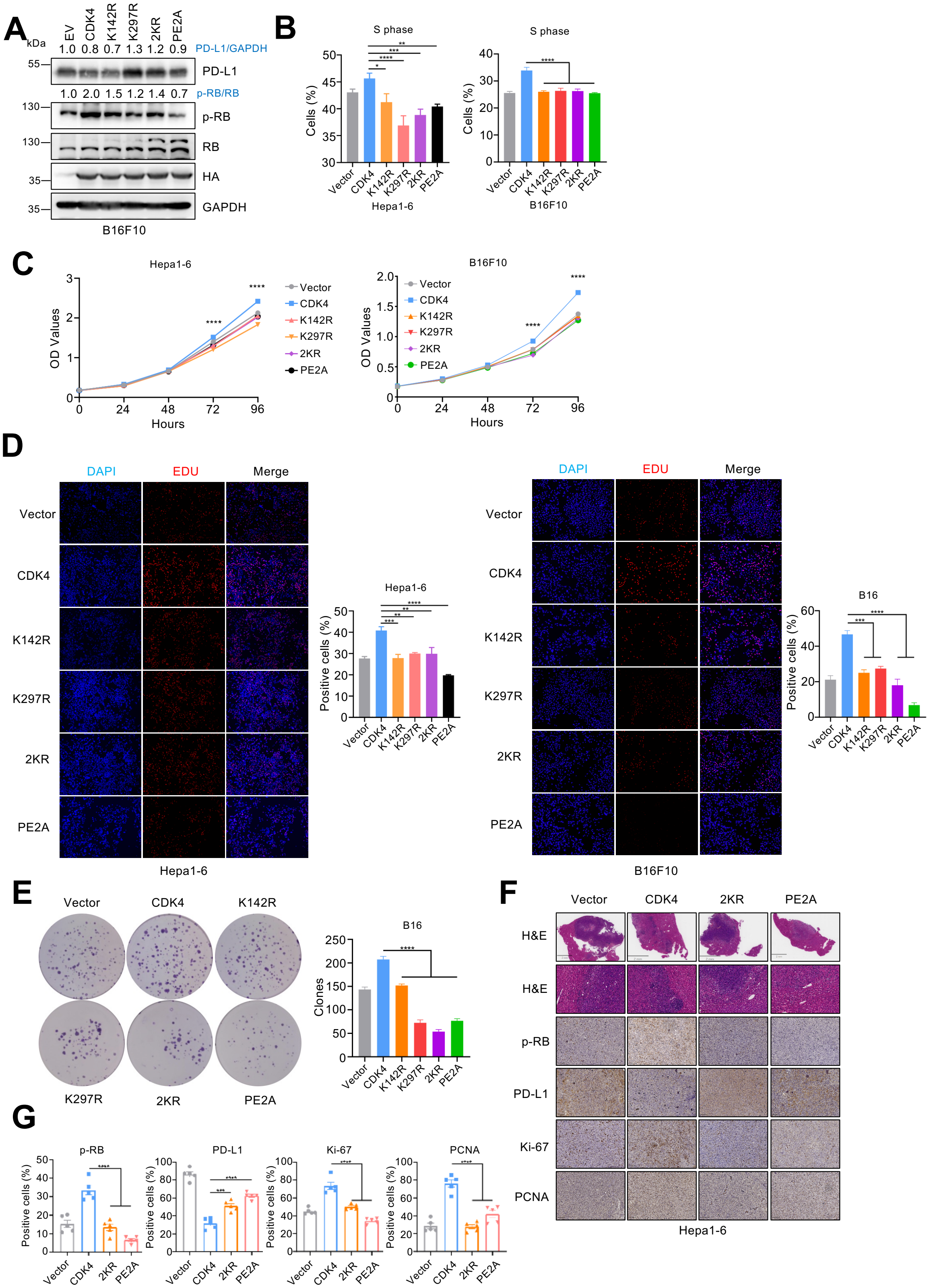
A
kDa
EV
CDK4
K142R
K297R
2KR
PE2A
1.0 0.8 0.7 1.3 1.2 0.9
PD-L1/GAPDH
55
PD-L1
1.0 2.0 1.5 1.2 1.4 0.7
p-RB/RB
130
p-RB
130
RB
35
HA
35
GAPDH
B16F10
B
S phase
Cells (%)
Vector
CDK4
K142R
K297R
2KR
PE2A
Hepa1-6
S phase
Cells (%)
B16F10
C
Hepa1-6
OD Values
Hours
Vector
CDK4
K142R
K297R
2KR
PE2A
B16F10
OD Values
Hours
D
DAPI
EDU
Merge
Vector
CDK4
K142R
K297R
2KR
PE2A
Hepa1-6
Positive cells (%)
B16F10
B16
E
Vector
CDK4
K142R
K297R
2KR
PE2A
B16
Clones
F
H&E
H&E
p-RB
PD-L1
Ki-67
PCNA
Hepa1-6
G
p-RB
PD-L1
Ki-67
PCNA
Positive cells (%)

Figure S7. CDK4-2KR and PE2A suppresses tumor cell proliferation. (**A**) WB analysis of WCL derived from B16F10 cells infected with HA-CDK4, CDK4-K142R, K297R, 2KR and PE2A lentivirus. **(B)** Flow cytometry analysis of cell cycle in Hepa1-6 and B16F10 cells infected with HA-CDK4, CDK4-K142R, K297R, 2KR and PE2A lentivirus (mean ± SEM, n=3, one-way ANOVA, **P* < 0.05, ***P* < 0.01, ****P* < 0.001, *****P* < 0.0001). **(C)** CCK-8 assays of Hepa1-6 and B16F10 cells infected with HA-CDK4, CDK4-K142R, K297R, 2KR and PE2A lentivirus (mean ± SEM, n=3, two-way ANOVA, **P* < 0.05, ***P* < 0.01, ****P* < 0.001, *****P* < 0.0001). **(D)** Representative images of EDU staining of Hepa1-6 and B16F10 cells infected with HA-CDK4 and HA-CDK4-2KR and PE2A lentivirus (mean ± SEM, n=3, one-way ANOVA, **P* < 0.05, ***P* < 0.01, ****P* < 0.001, *****P* < 0.0001). **(E)** Representative images of colony formation assays of B16F10 cells infected with HA-CDK4 and HA-CDK4-2CS lentivirus (mean ± SEM, n=3, one-way ANOVA, *****P* < 0.0001). **(F-G)** Representative images of IHC staining analysis of harvested Hepa1-6 tumor tissues **(F)**. Positive cells proportion were quantified (mean ± SEM, n=5, one-way ANOVA, ***P < 0.001, ****P < 0.0001) **(G)**.

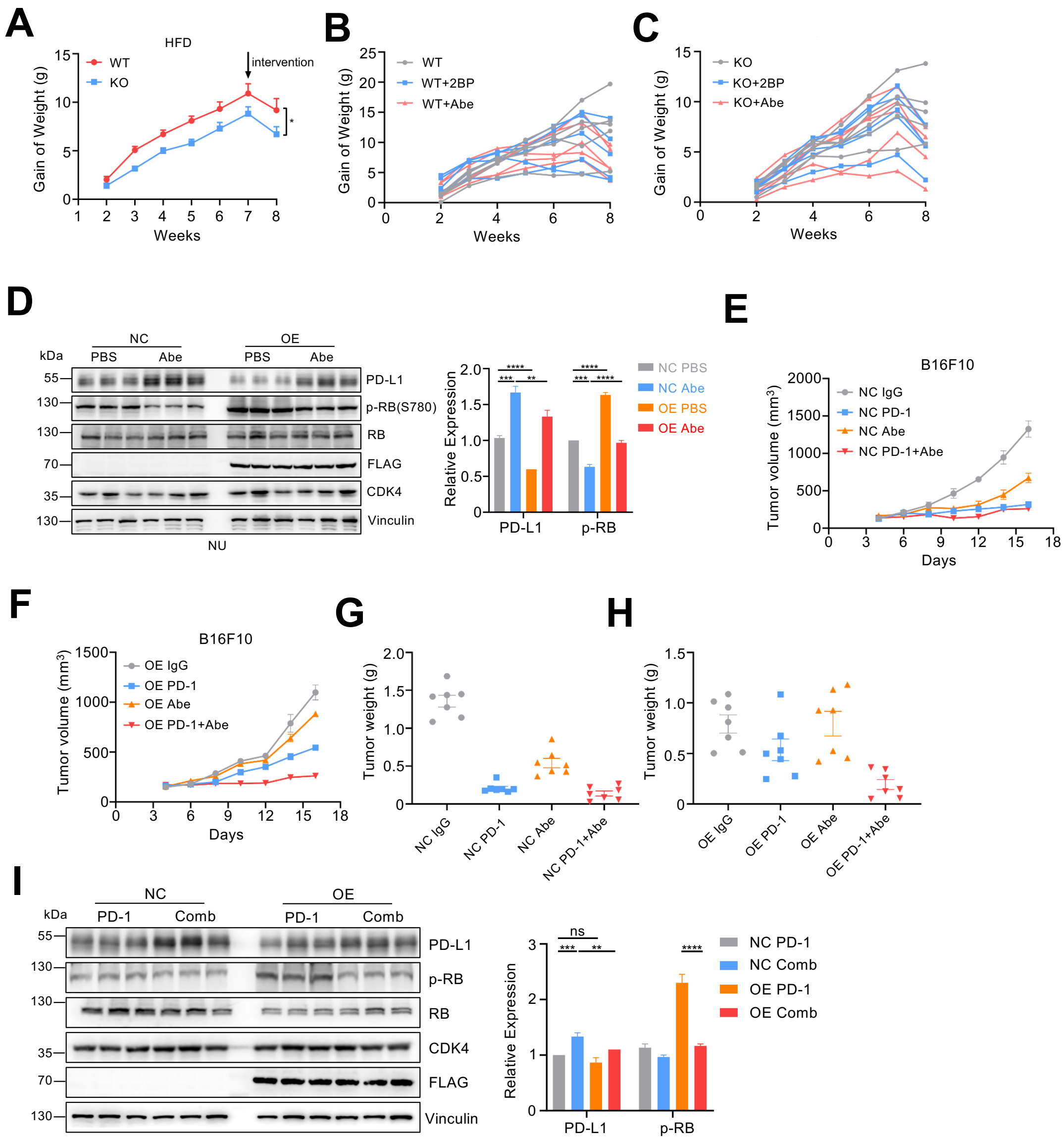

Figure S8. Zdhhc17 KO promotes immune response. (A) Body weight changes during HFD period (mean ± SEM, n=15, two-way ANOVA, *P < 0.05). **(B-C)** Body weight changes during HFD period specific to each mouse. Changes of WT mice were displayed in **(B)** and KO mice were displayed in **(C). (D)** WB analysis of WCL derived from tumor tissues in nude mice. **(Fig 6B)**. **(E-F)** The cell growth curve of 4 groups that mice implanted Vector group cells **(E)** and overexpressed ZDHHC17 group cells **(F)**. **(G-H)** The tumor weight among 4 groups that mice implanted Vector group cells **(G)** and overexpressed ZDHHC17 group cells **(H)**. **(I)** WB analysis of WCL derived from tumor tissues under PD-1 inhibitor and Abemaciclib government mice **(Fig 6B),** Student's *t* test, ns, no significance, **P < 0.01, ***P < 0.001, ****P < 0.0001.

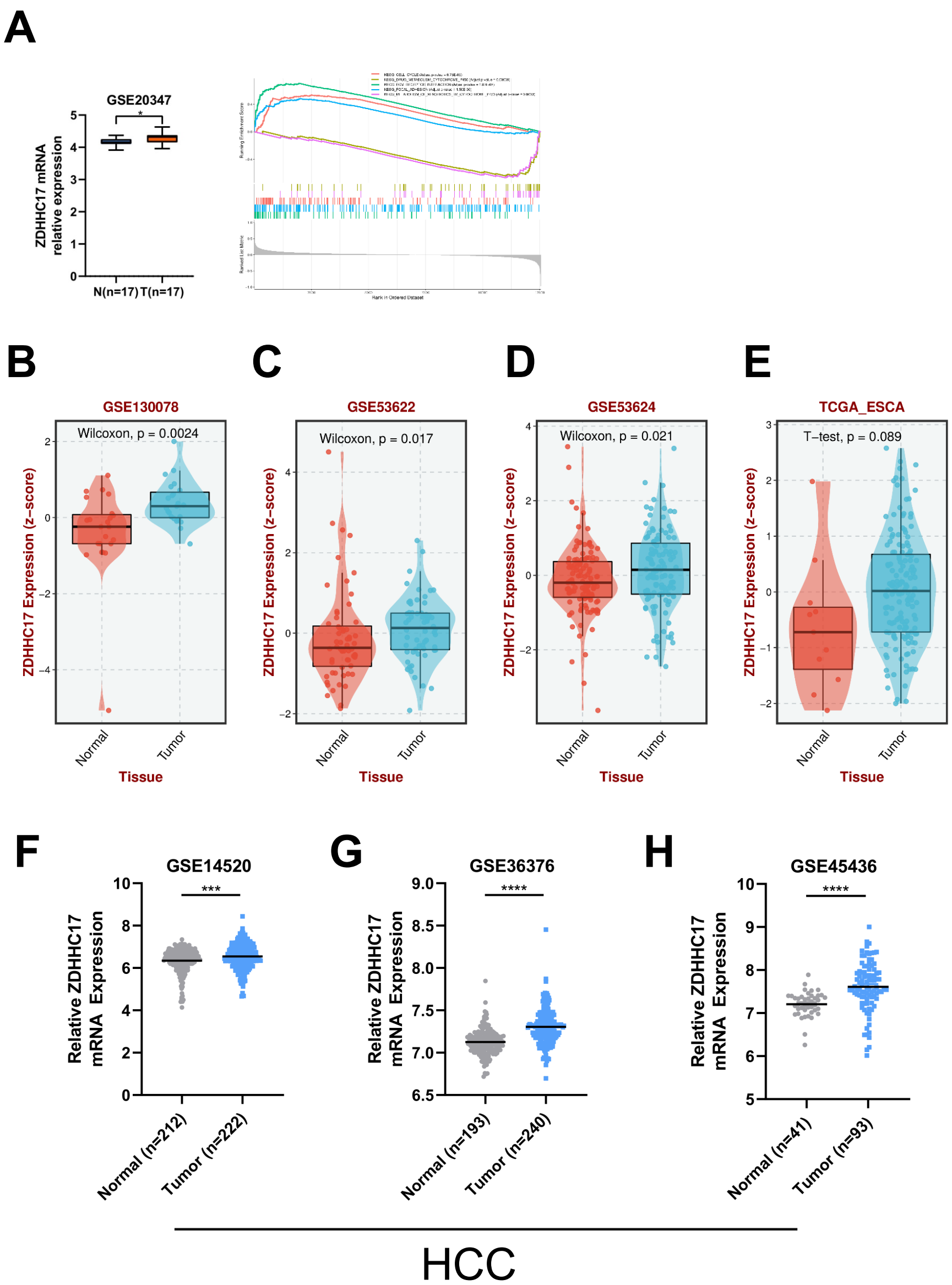

Figure S9. ZDHHC17 is high expression in tumors. (A-D) Relative ZDHHC17 mRNA expression between esophageal tumor tissues and normal tissues in different GEO datasets, GSE20347 **(A,** Student's *t* test**)**, GSE130078 **(B,** Wilcoxon test**)**, GSE53622 **(C,** Wilcoxon test**)** and GSE53624 **(D,** Wilcoxon test**)**. GSEA analysis was performed in GSE20347 **(A)**. **(E)** Relative ZDHHC17 mRNA expression between esophageal tumor tissues and normal tissues in The Cancer Genome Atlas Program (TCGA) database, Student's *t* test. **(F-H)** Relative ZDHHC17 mRNA expression between Hepatocellular Carcinoma tumor tissues and normal tissues in GEO database, Student's *t* test, *P < 0.05, ***P < 0.001, ****P < 0.0001.
