# Supplementary material for: ZDHHC17‐Mediated CDK4 Palmitoylation Drives Cell Cycle Progression and Orchestrates Cancer Immune Surveillance
Source: Adv Sci (Weinh). 2026 May 14:e75693. Online ahead of print. doi: 10.1002/advs.75693 (PMC13335960; doi:10.1002/advs.75693)

Fig.1C

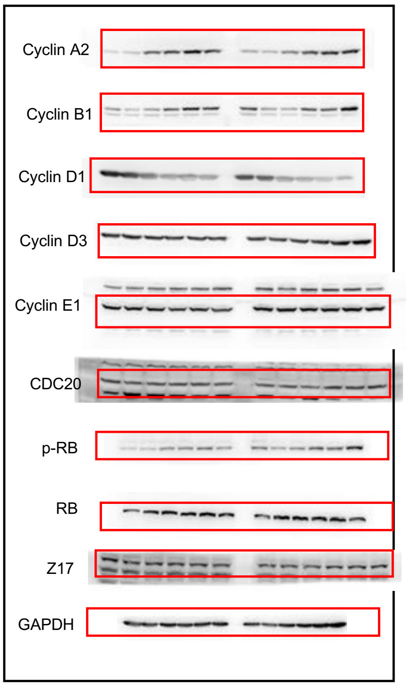

Fig.1E

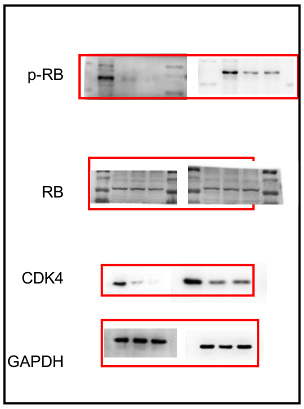

Fig.1G

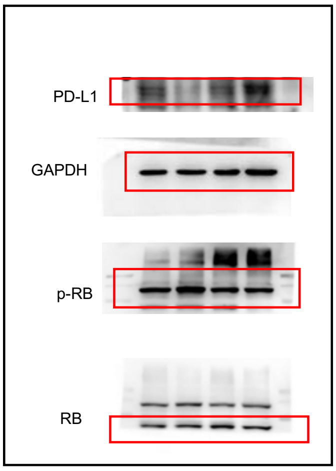

Fig.1H

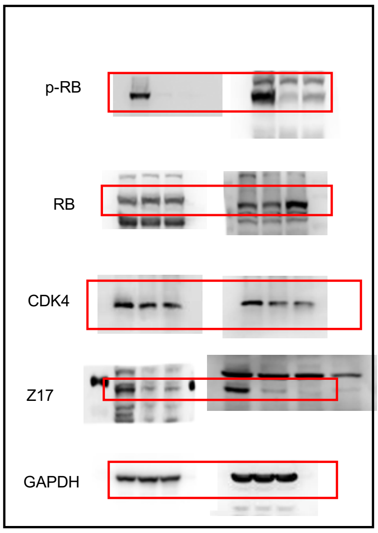

Fig.1N

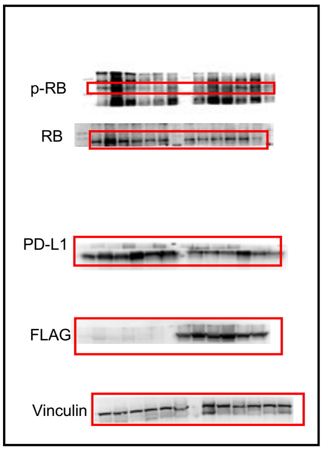

Fig.1O

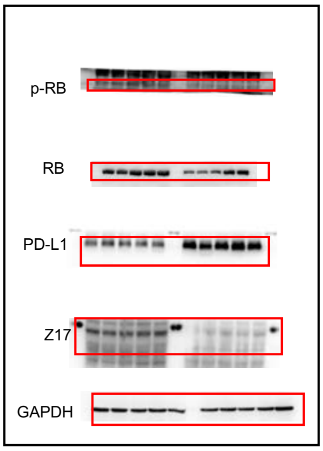

Fig.2B

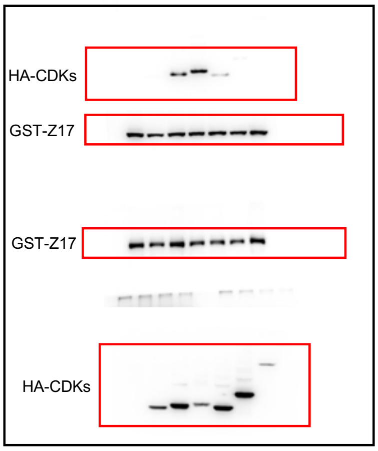

Fig.2C

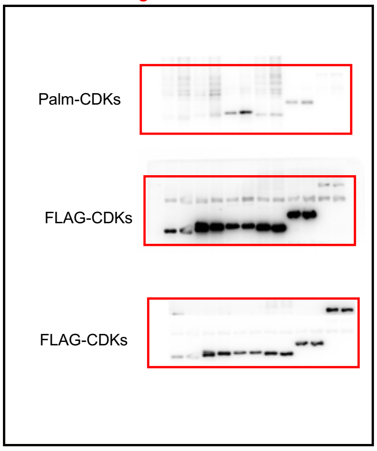

Fig.2D

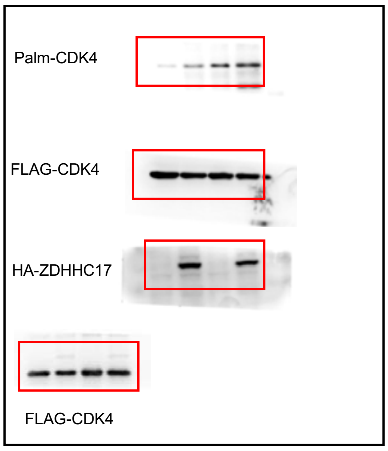

Fig.2E

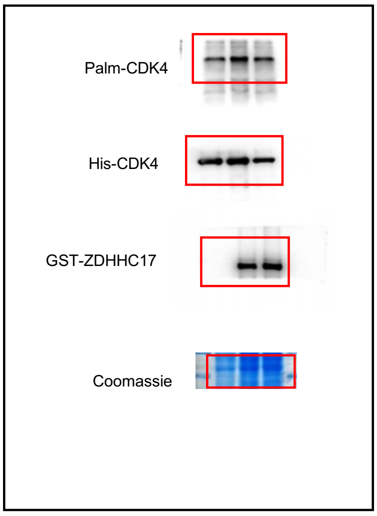

Fig.2G

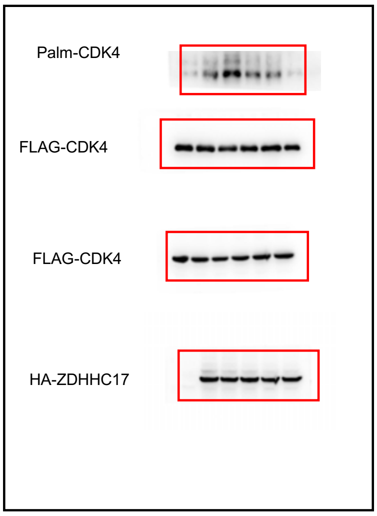

Fig.2H

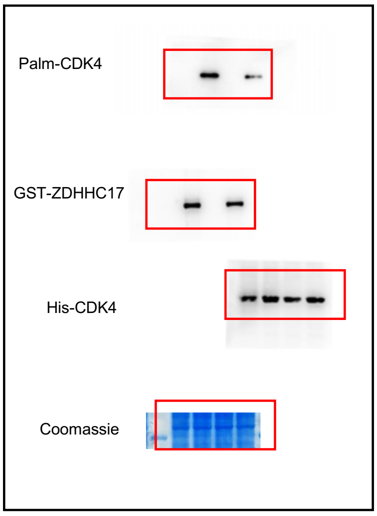

Fig.2I

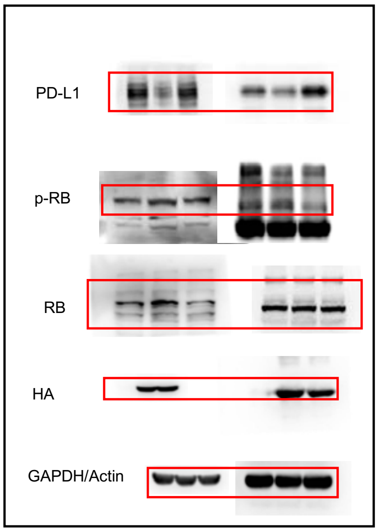

Fig.2M

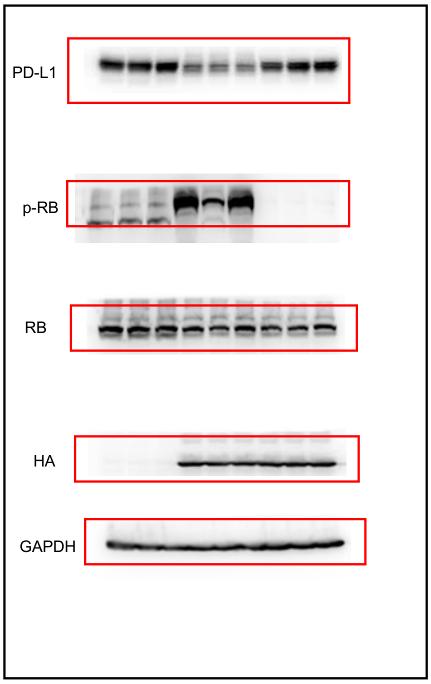

Fig.3A

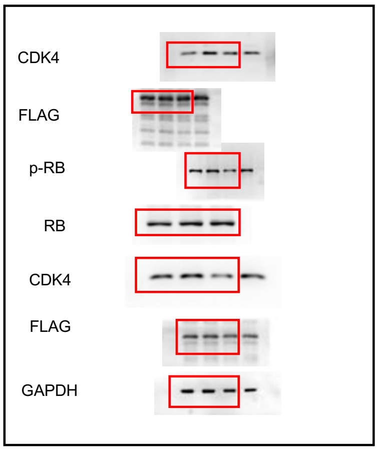

Fig.3B

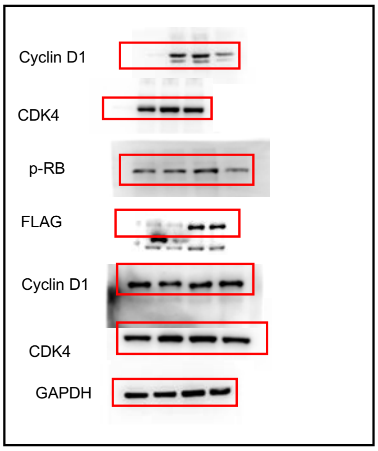

Fig.3C

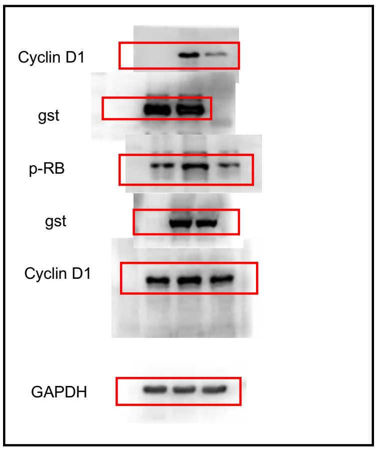

Fig.3D

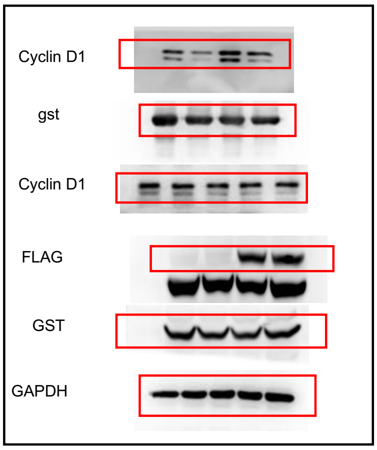

Fig.3F

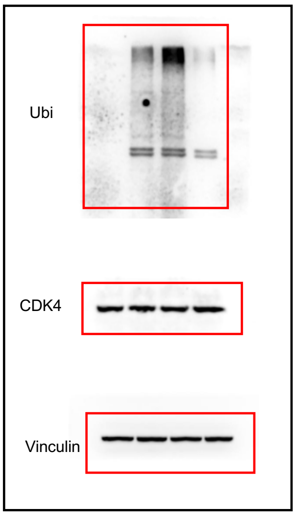

Fig.3G

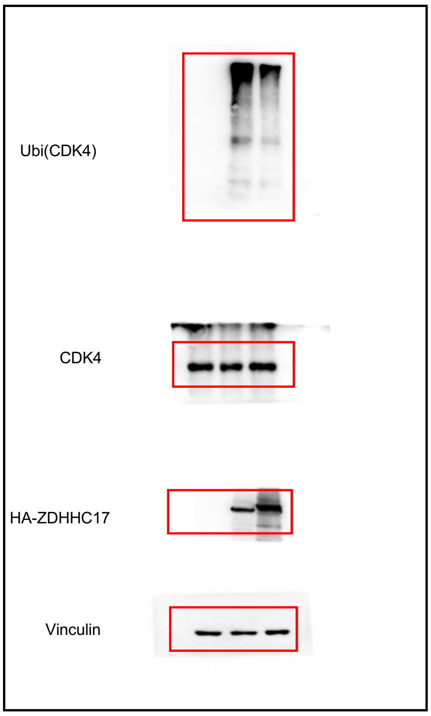

Fig.3H

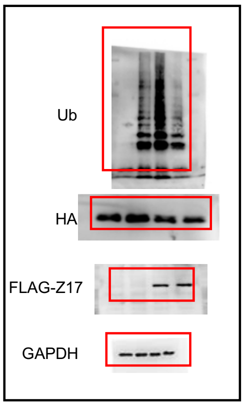

Fig.3I

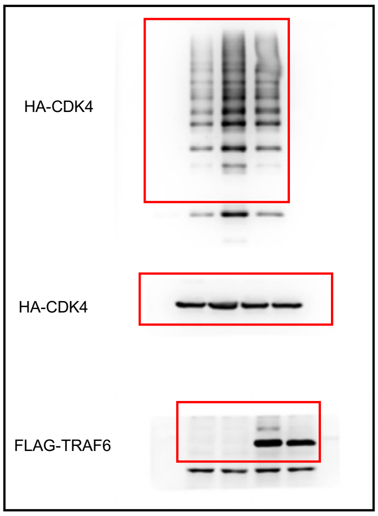

Fig.3J

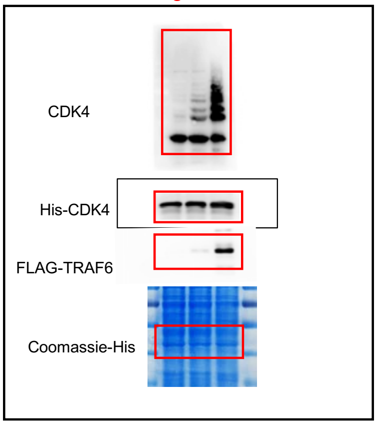

Fig.3K

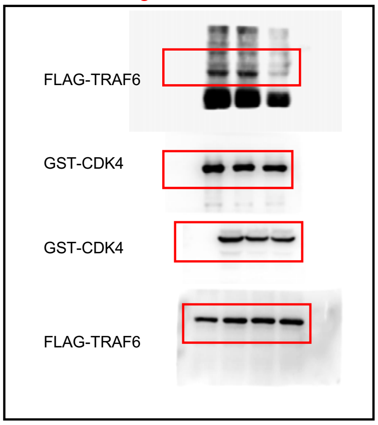

Fig.3L

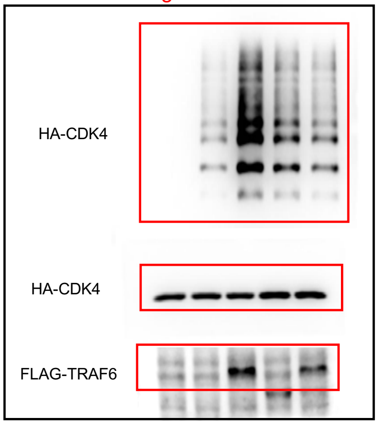

Fig.3N

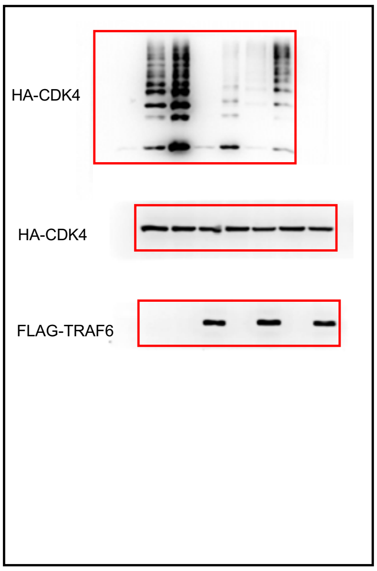

Fig.3P

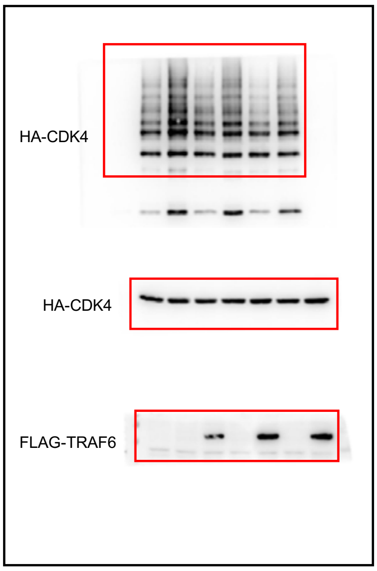

Fig.3Q

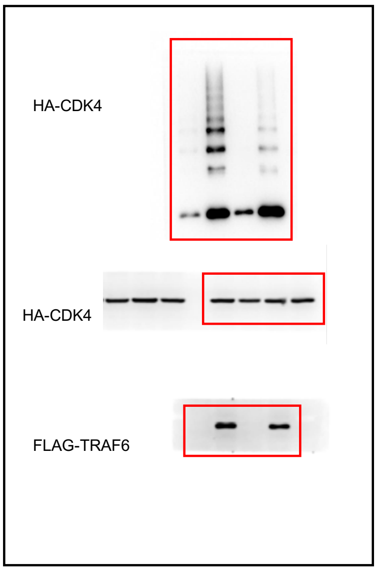

Fig.3S

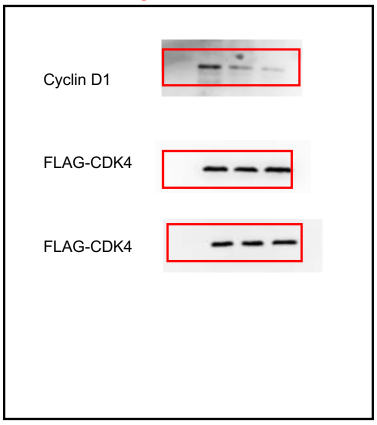

Fig.4A

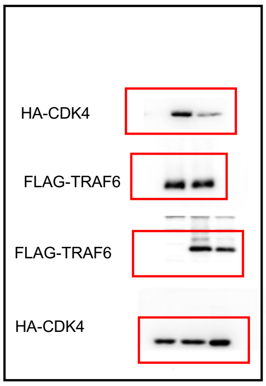

Fig.4B

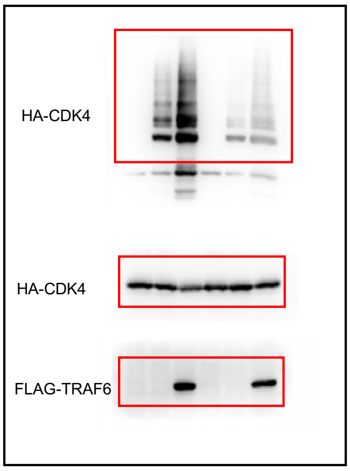

Fig.4C

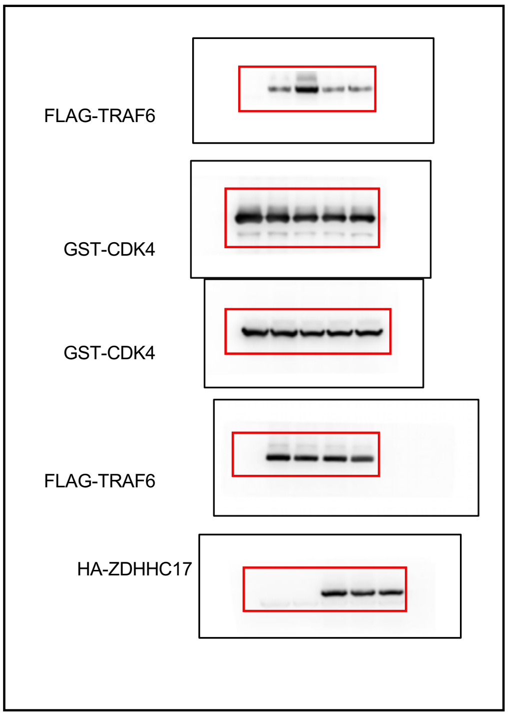

Fig.4D

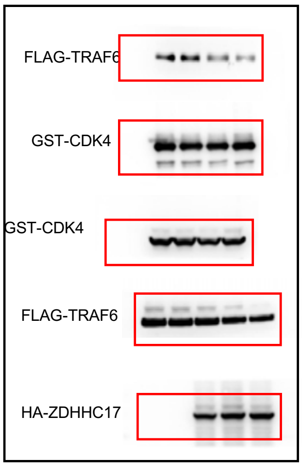

Fig.4E

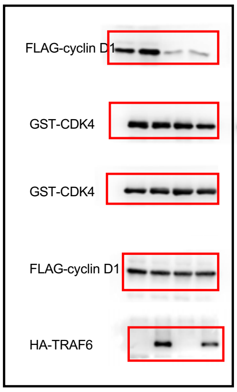

Fig.4F

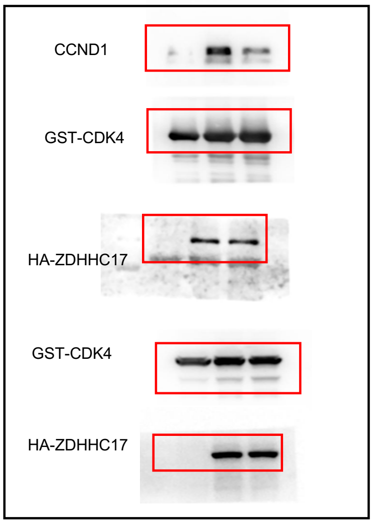

Fig.4G

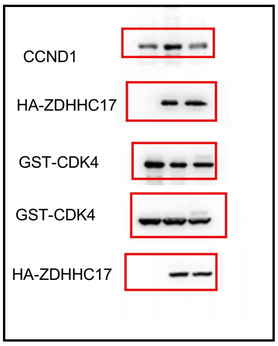

Fig.4H

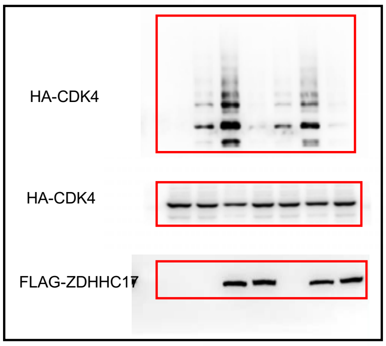

Fig.4I

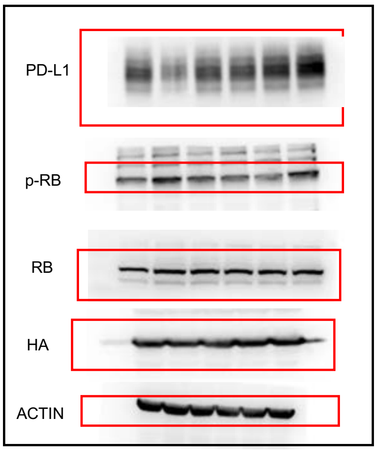

Fig.4M

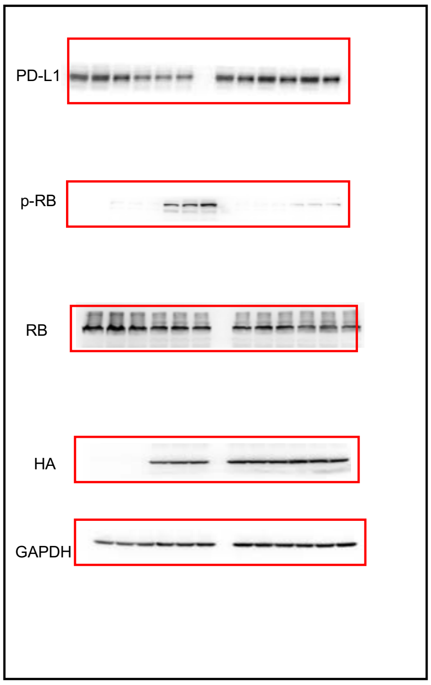

Fig.6E

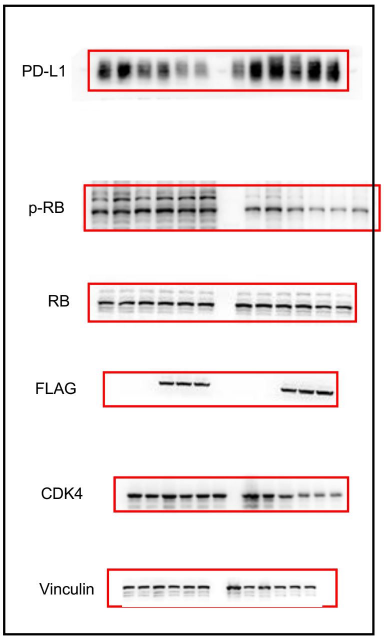

Fig.S1D

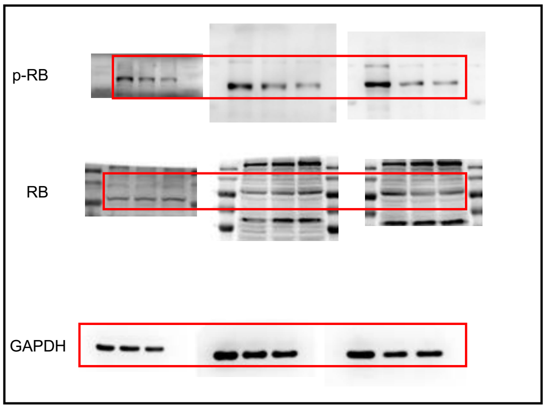

Fig.S1E

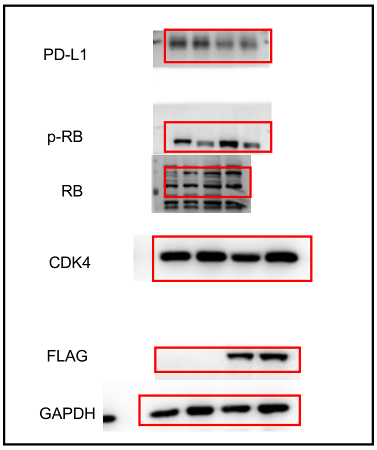

Fig.S2A

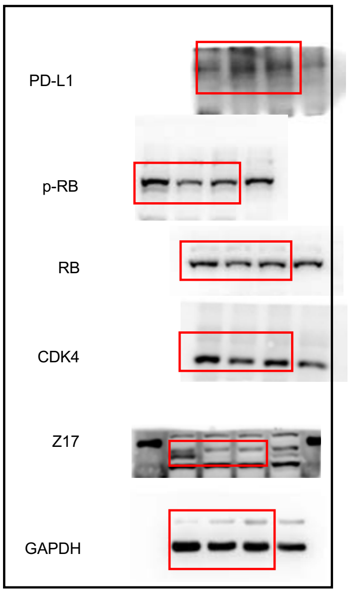

Fig.S2B

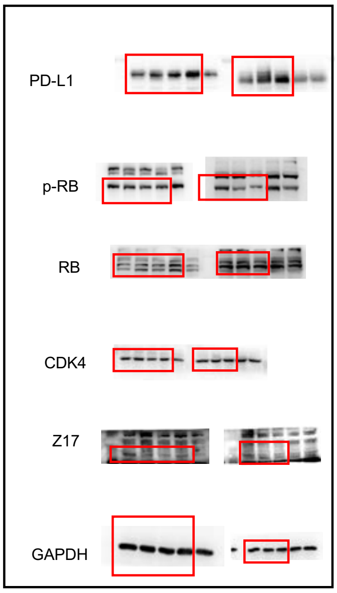

Fig.S2C

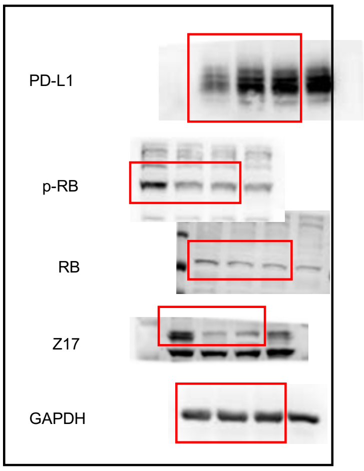

Fig.S3A

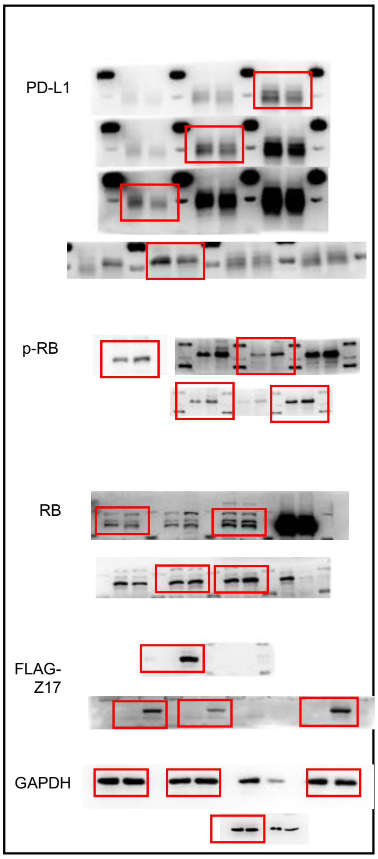

Fig.S3K

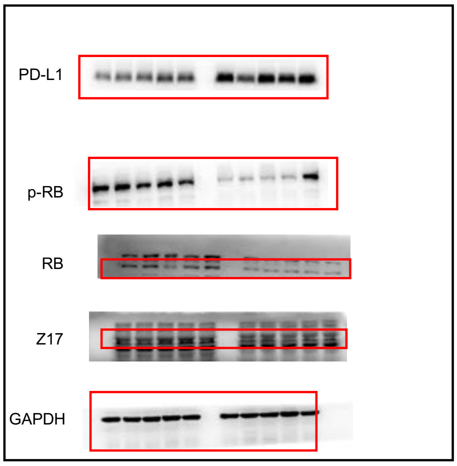

Fig. S3L

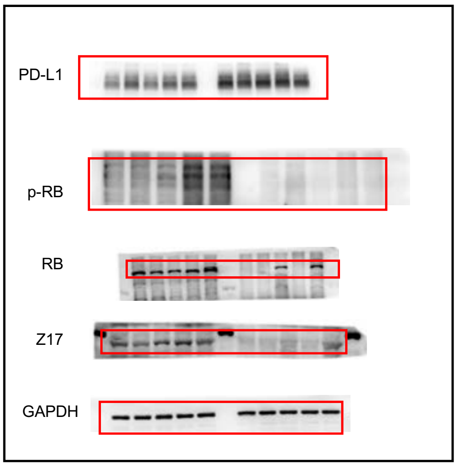

Fig. S3M

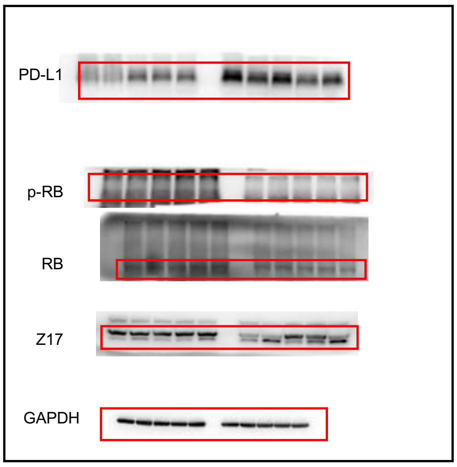

Fig.S4A

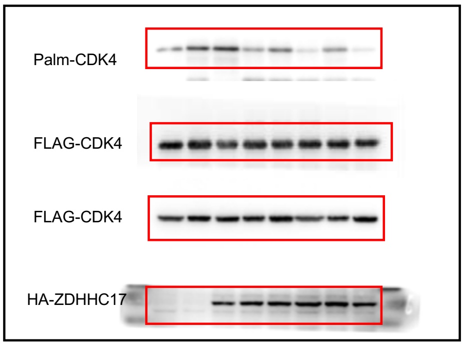

Fig.S4B

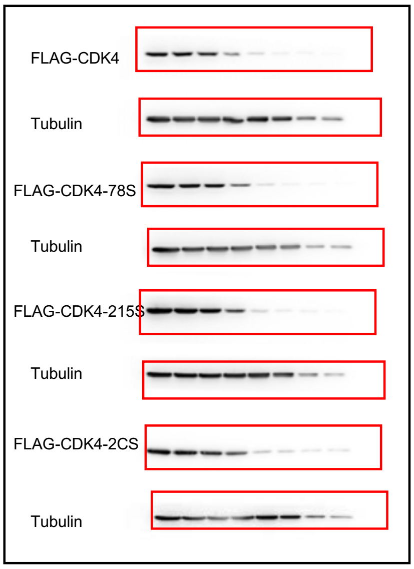

Fig.S4C

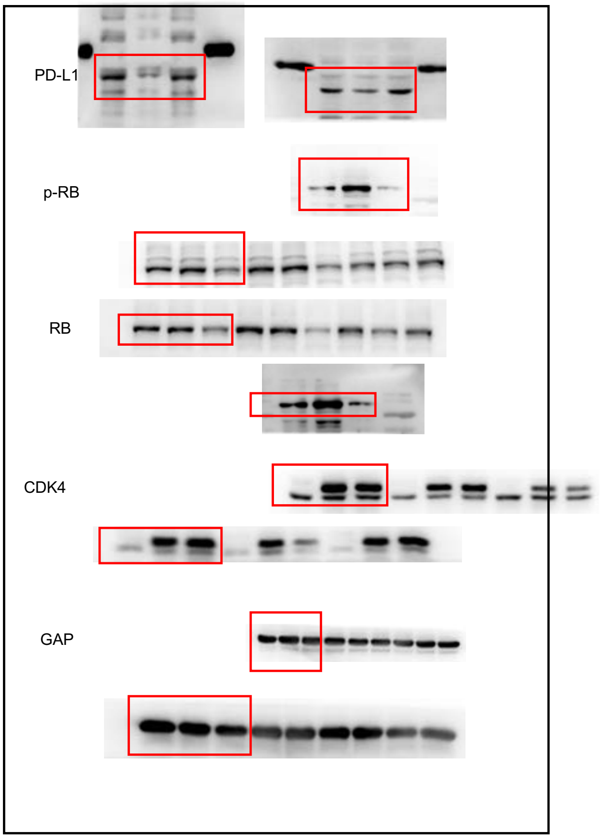

Fig.S5D

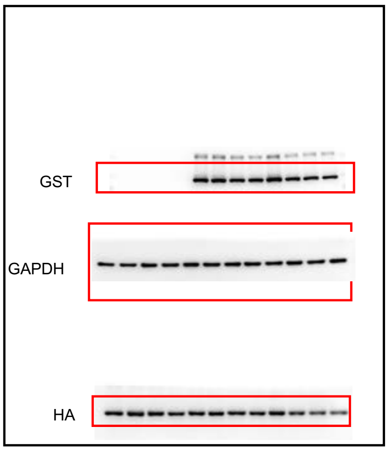

Fig. S5E

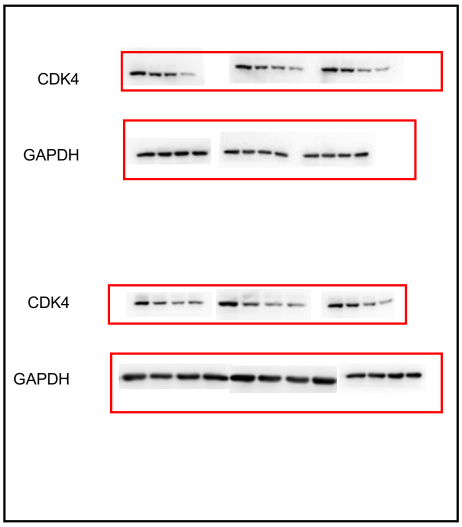

Fig. S5F

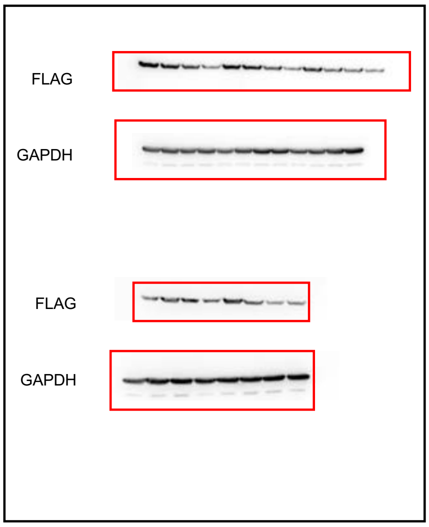

Fig. S5G

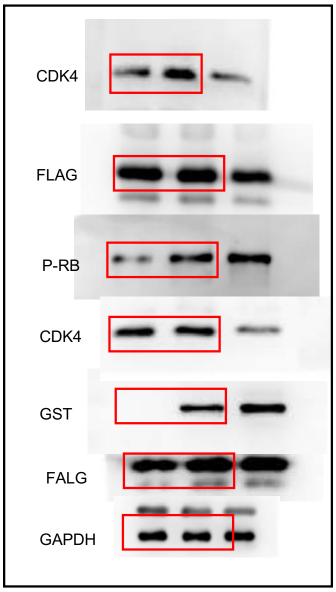

Fig. S5I

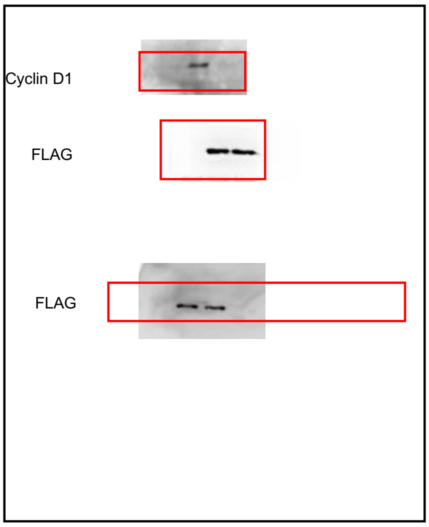

Fig. S5G

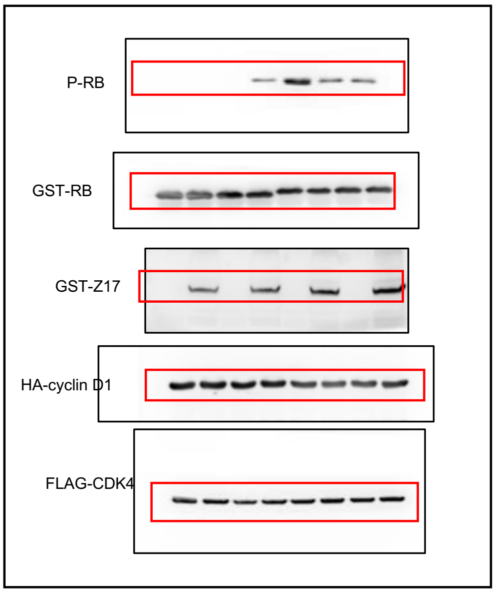

Fig.S6A

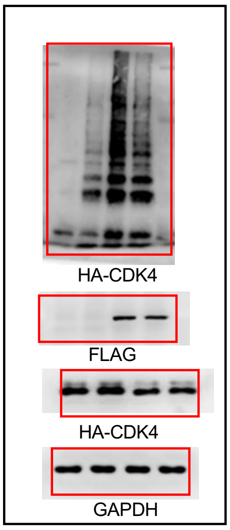

Fig.S6C

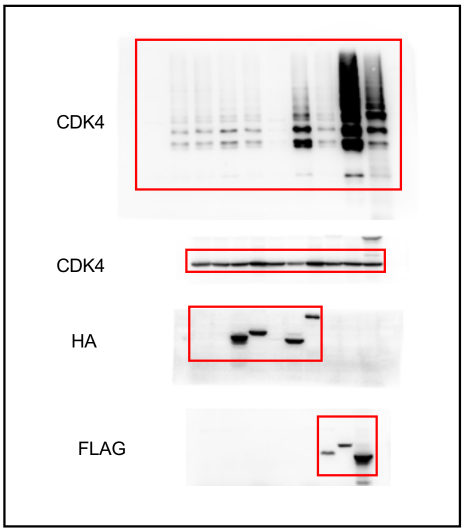

Fig.S6D

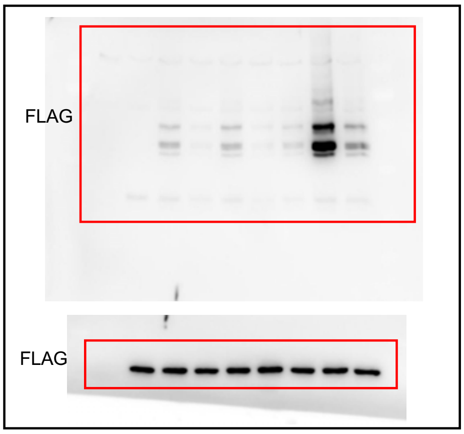

Fig.S6E

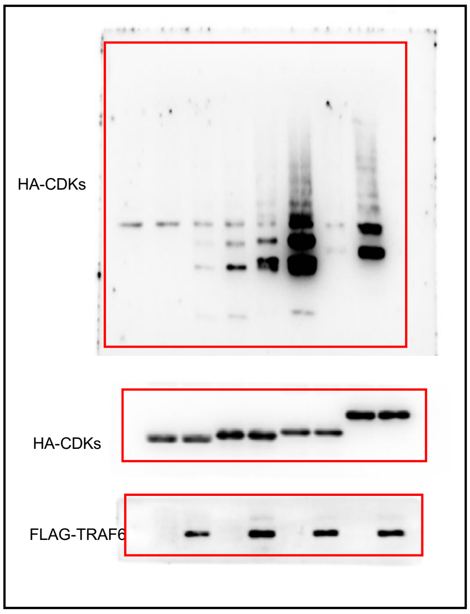

Fig.S6F

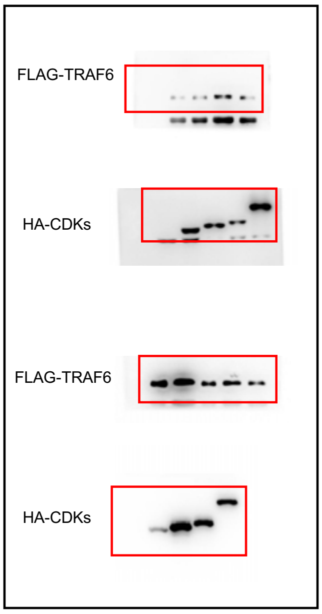

Fig.S6G

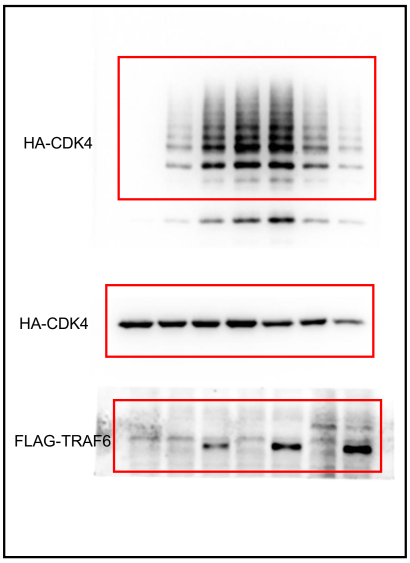

Fig. S6N

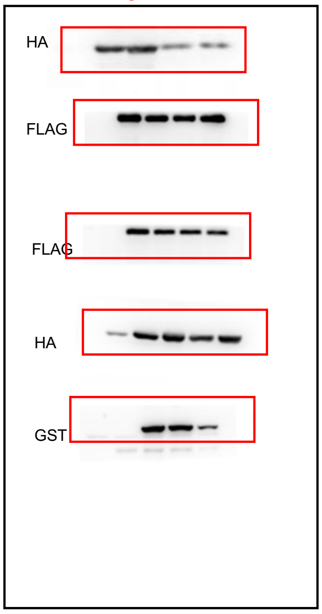

Fig. S6P

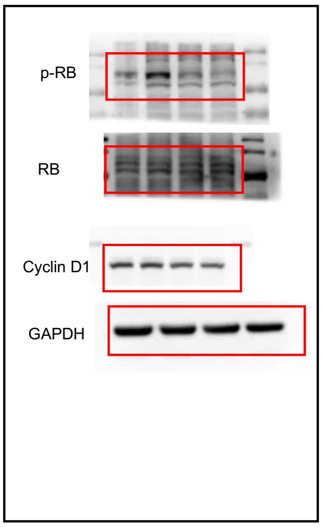

Fig.S6H

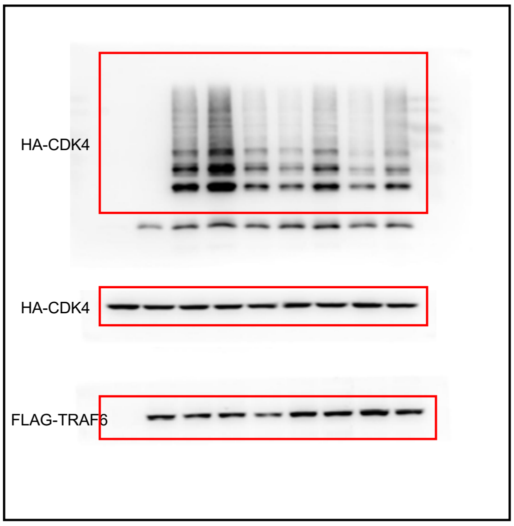

Fig.S6I

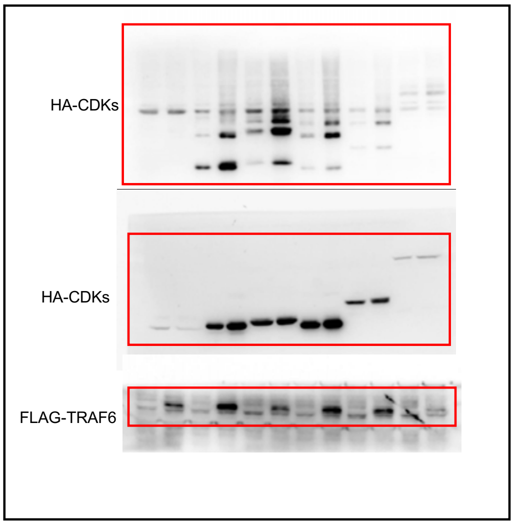

Fig.S6J

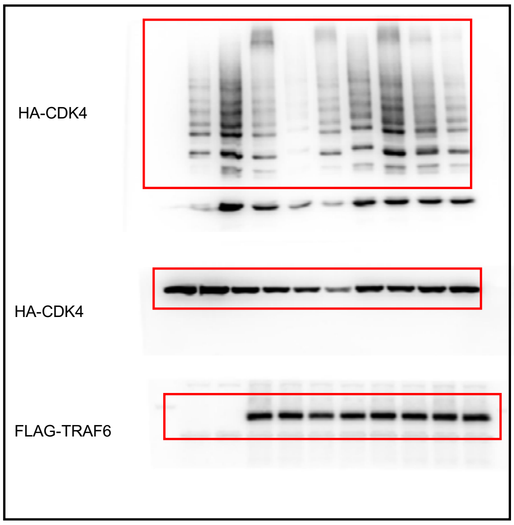

Fig.S6K

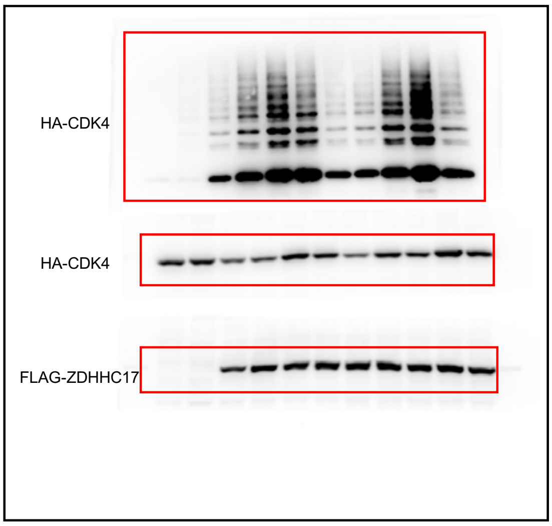

Fig.S6L

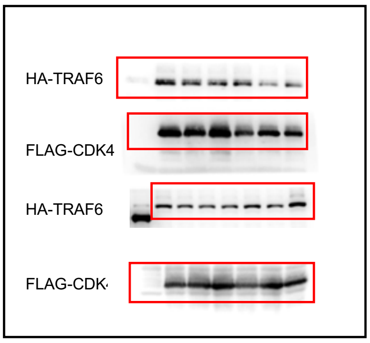

Fig.S6M

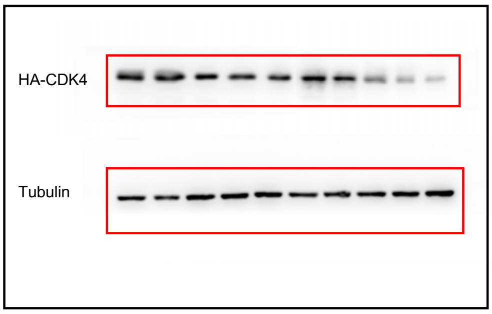

Fig.S6O

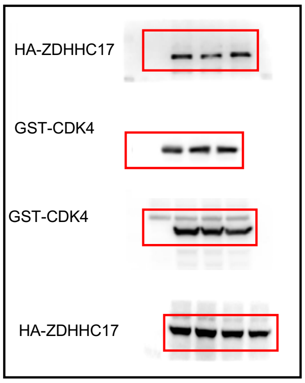

Fig.S7A

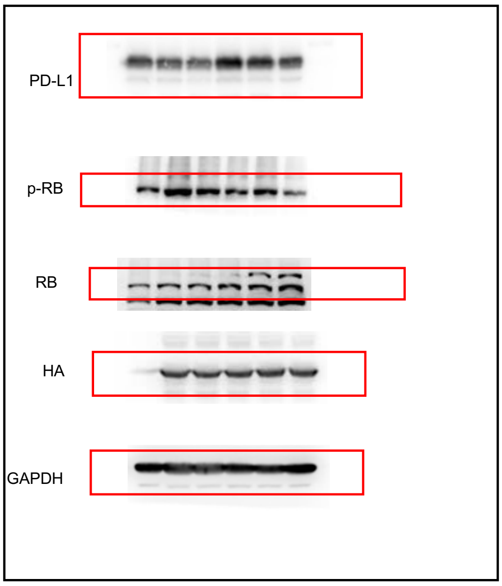

Fig. 1A

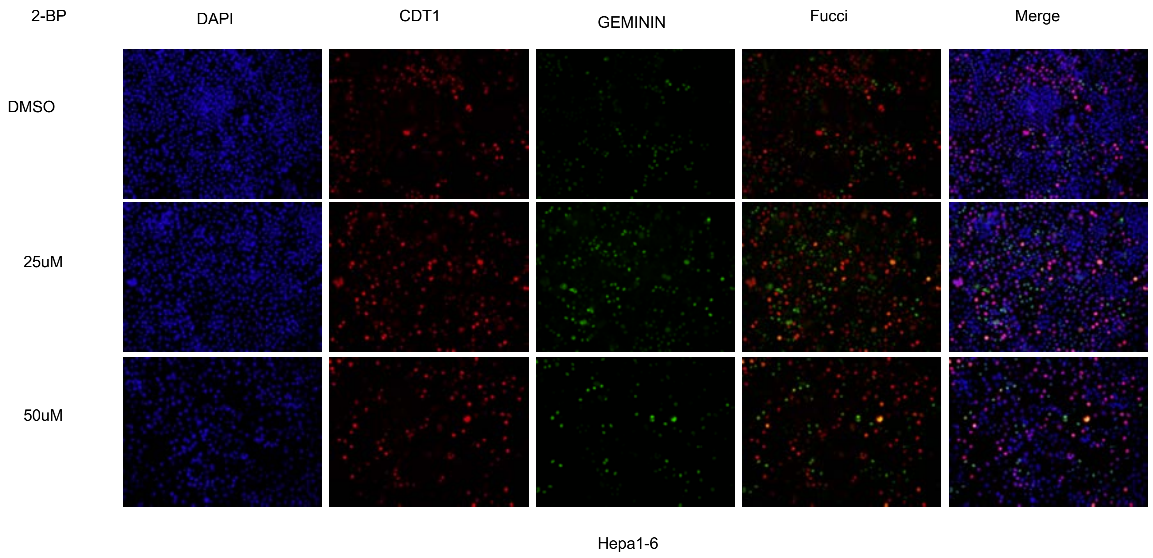
2-BP
DAPI
CDT1
GEMININ
Fucci
Merge
DMSO
25uM
50uM
Hepa1-6

Fig. 1M

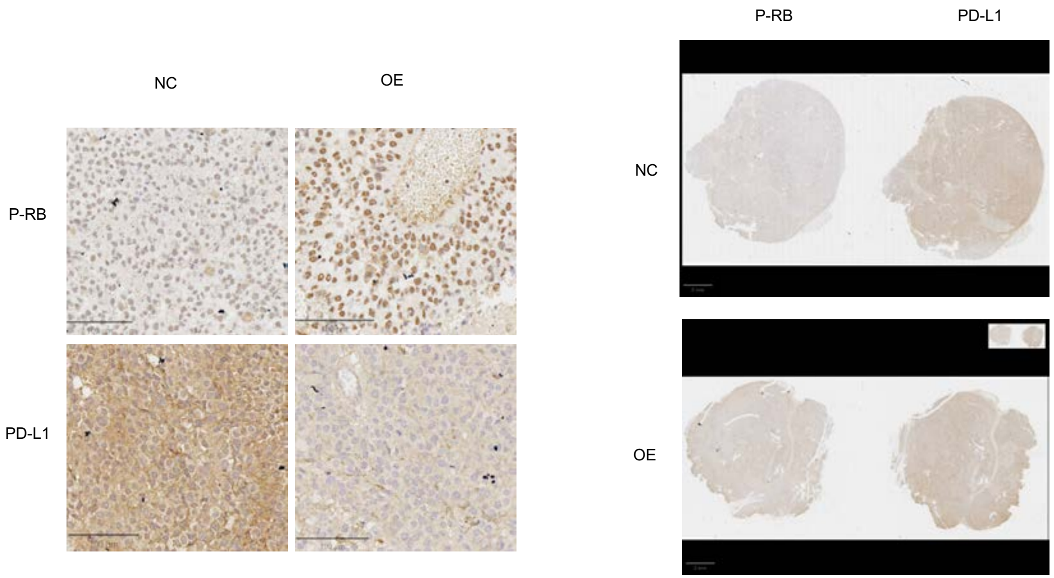
NC
OE
P-RB
PD-L1
P-RB
PD-L1
NC
OE

Fig. 2J

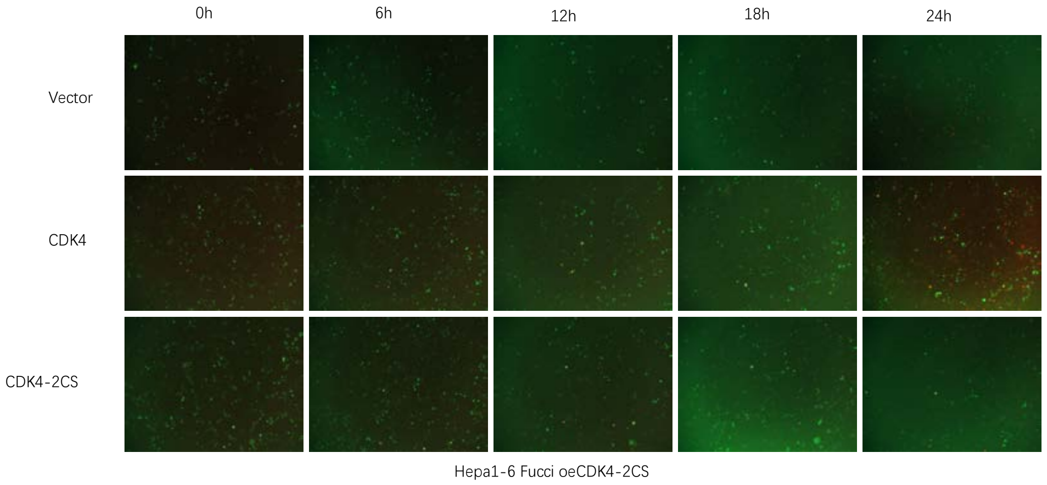
0h
6h
12h
18h
24h
Vector
CDK4
CDK4-2CS
Hepa1-6 Fucci oeCDK4-2CS

Fig. 2N

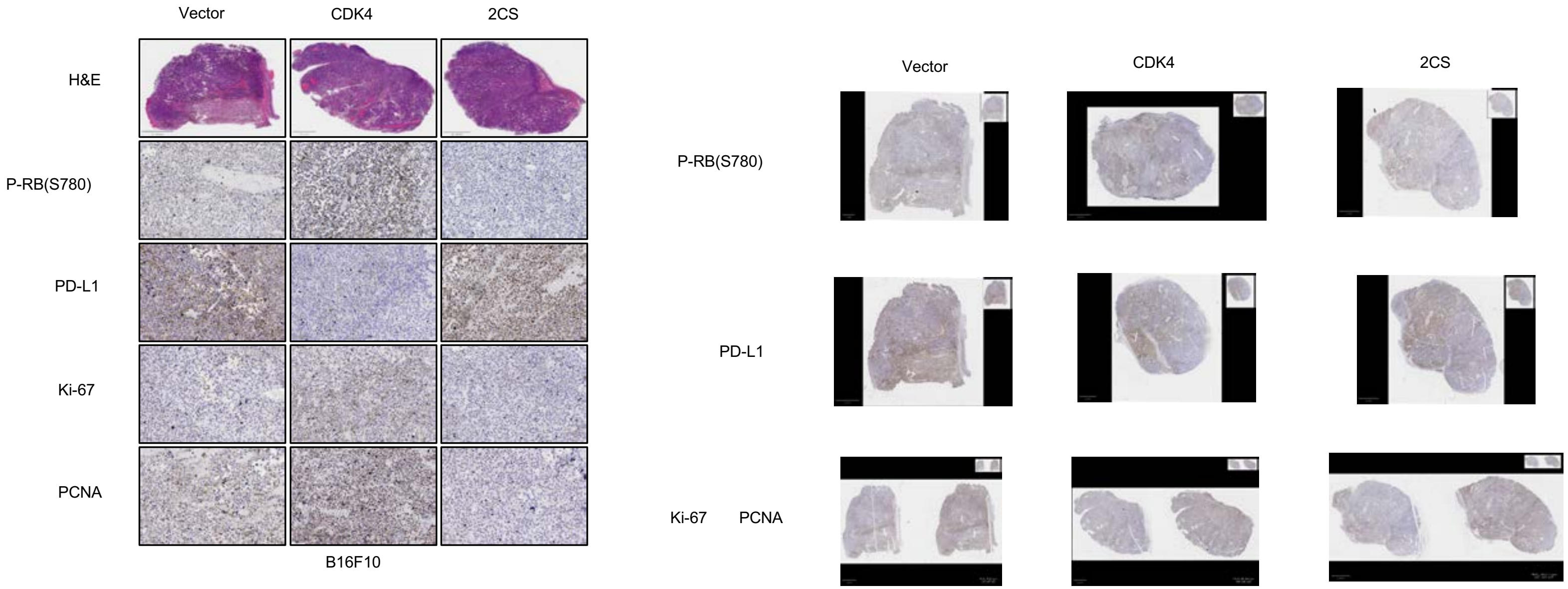
Vector
CDK4
2CS
H&E
P-RB(S780)
PD-L1
Ki-67
PCNA
B16F10
Vector
CDK4
2CS
P-RB(S780)
PD-L1
Ki-67
PCNA

Fig. 2P

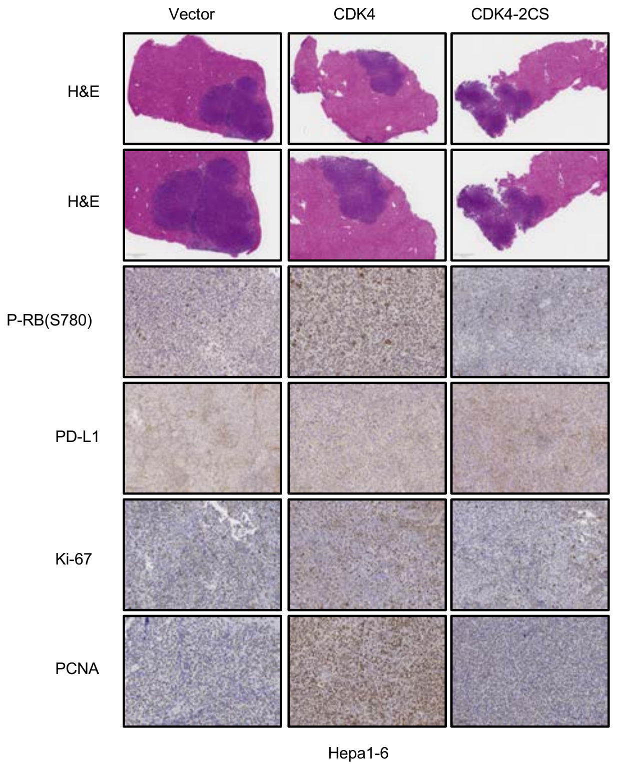

Vector
CDK4
CDK4-2CS
H&E
H&E
P-RB(S780)
PD-L1
Ki-67
PCNA
Hepa1-6

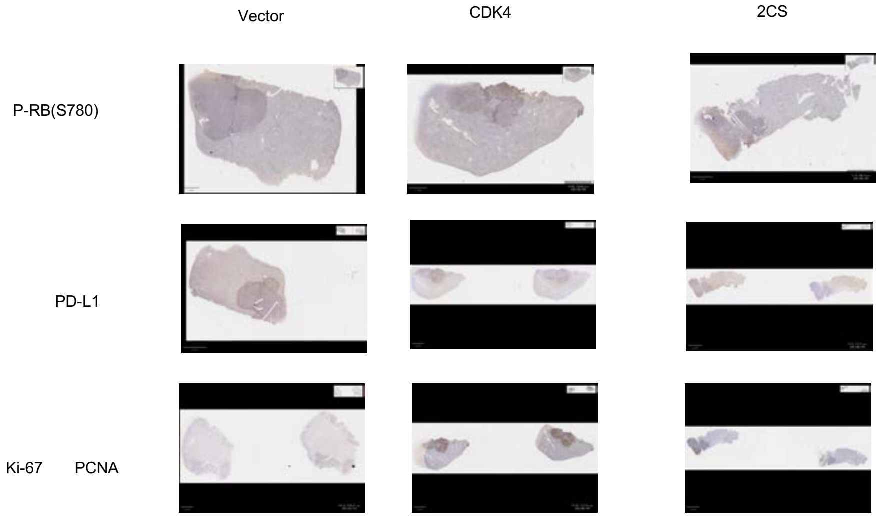

Vector
CDK4
2CS
P-RB(S780)
PD-L1
Ki-67
PCNA

Fig. 3E

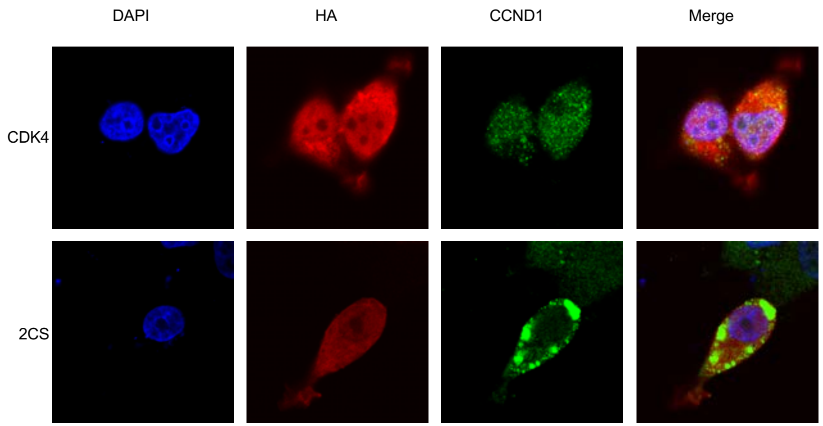

Fig. 3T

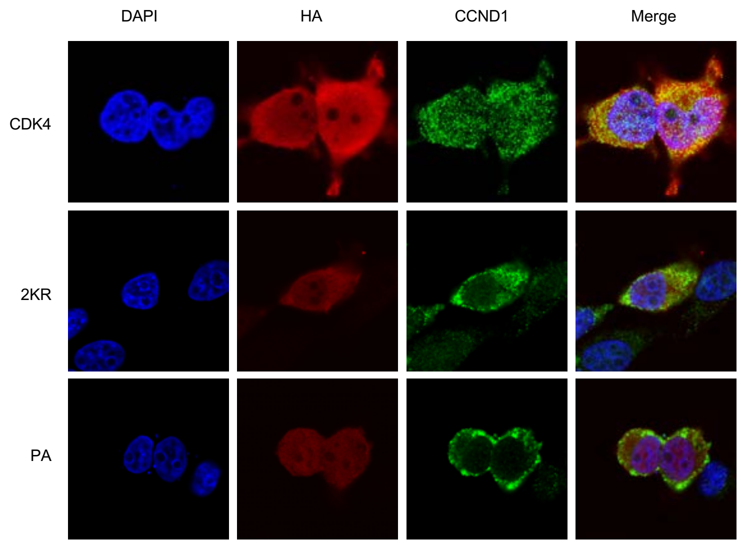

Fig. 4J

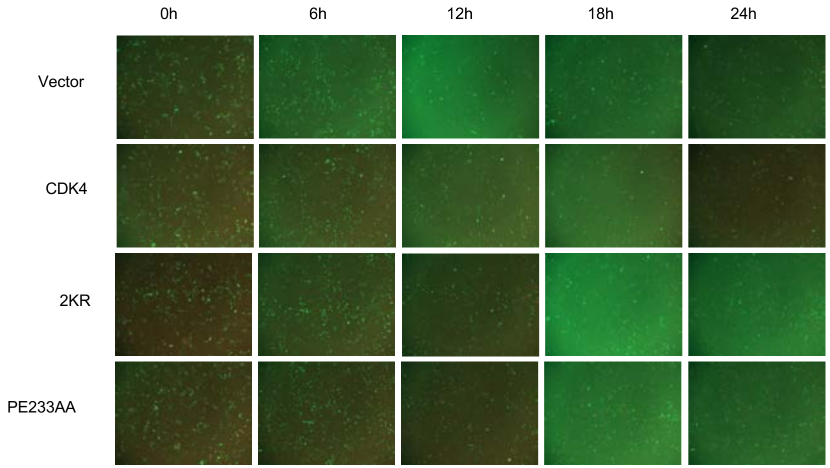
0h
6h
12h
18h
24h
Vector
CDK4
2KR
PE233AA

Fig. 4N

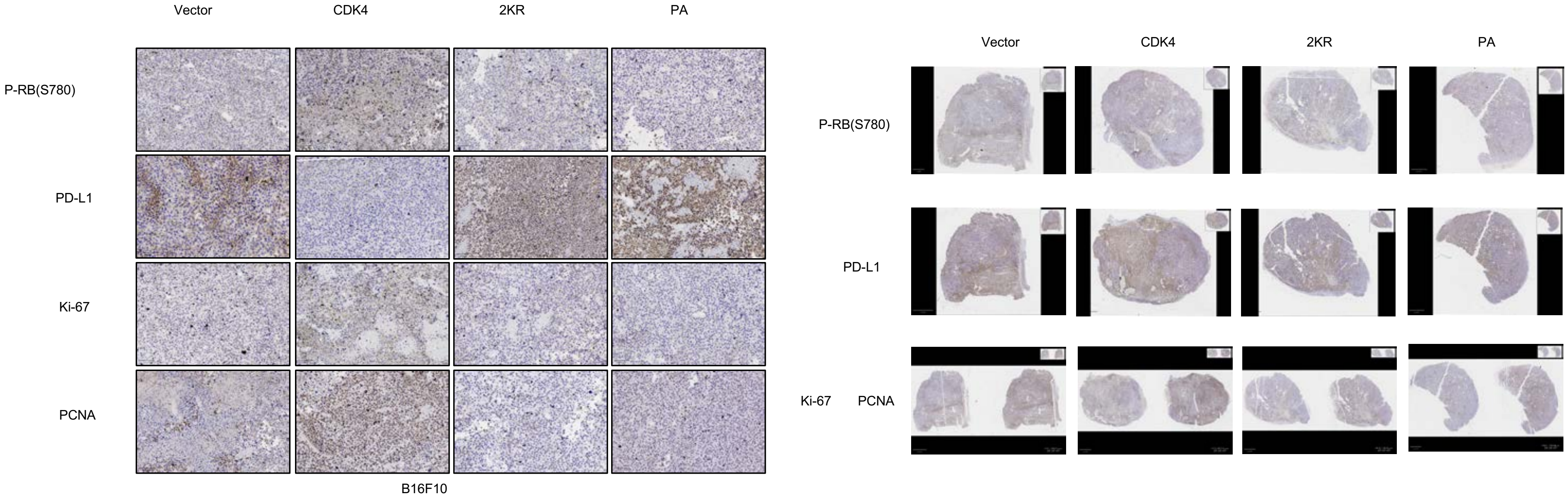
Vector
CDK4
2KR
PA
P-RB(S780)
PD-L1
Ki-67
PCNA
B16F10
Vector
CDK4
2KR
PA
P-RB(S780)
PD-L1
Ki-67
PCNA

Fig. 5A

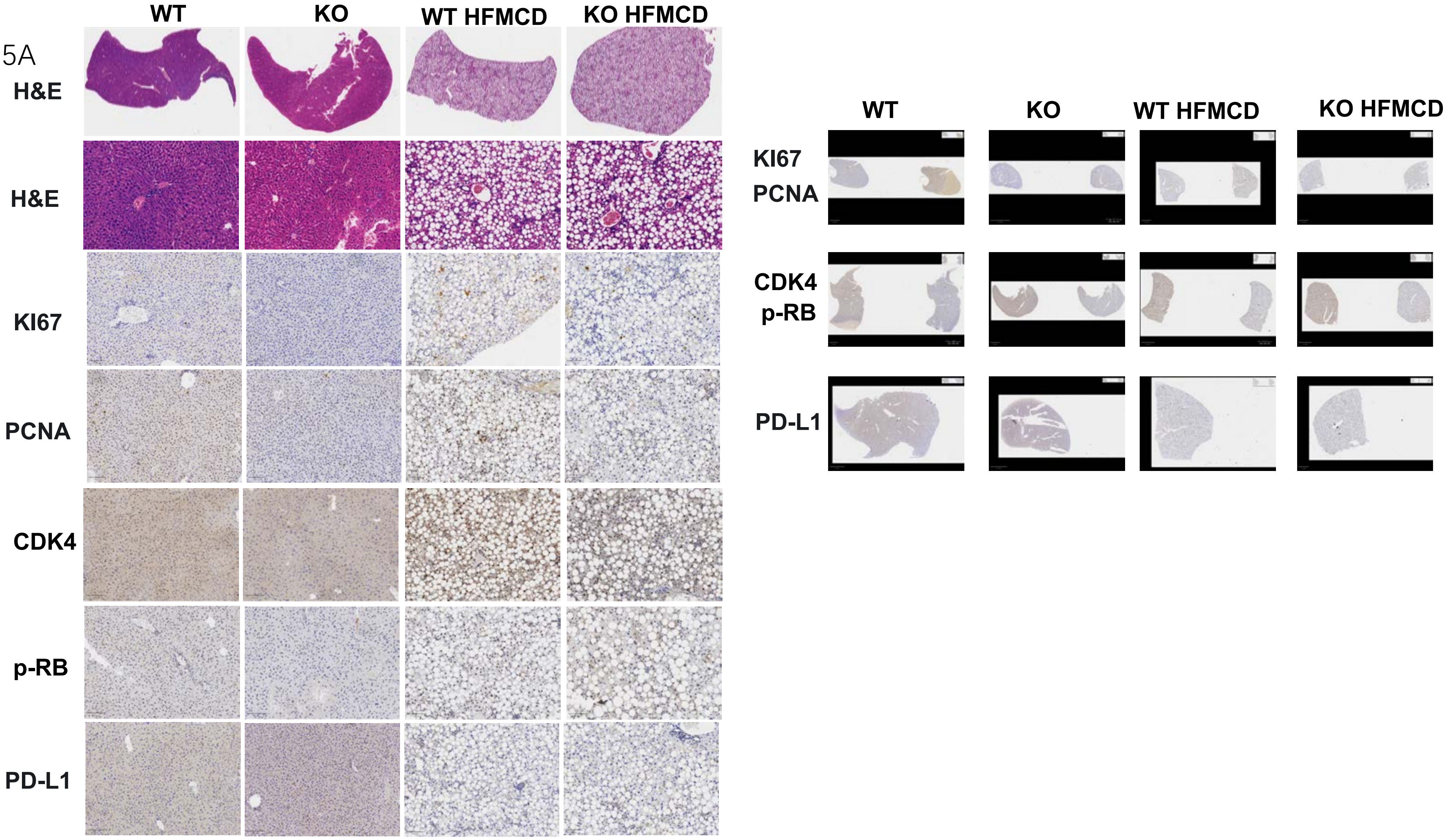
WT
KO
WT HFMCD
KO HFMCD
H&E
H&E
KI67
PCNA
CDK4
p-RB
PD-L1
WT
KO
WT HFMCD
KO HFMCD
KI67
PCNA
CDK4
p-RB
PD-L1

Fig. 5G

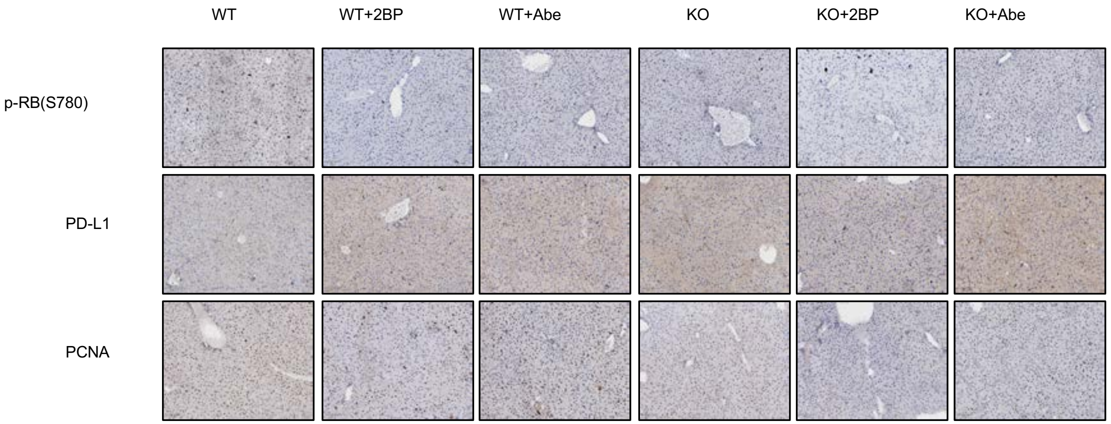
WT
WT+2BP
WT+Abe
KO
KO+2BP
KO+Abe
p-RB(S780)
PD-L1
PCNA

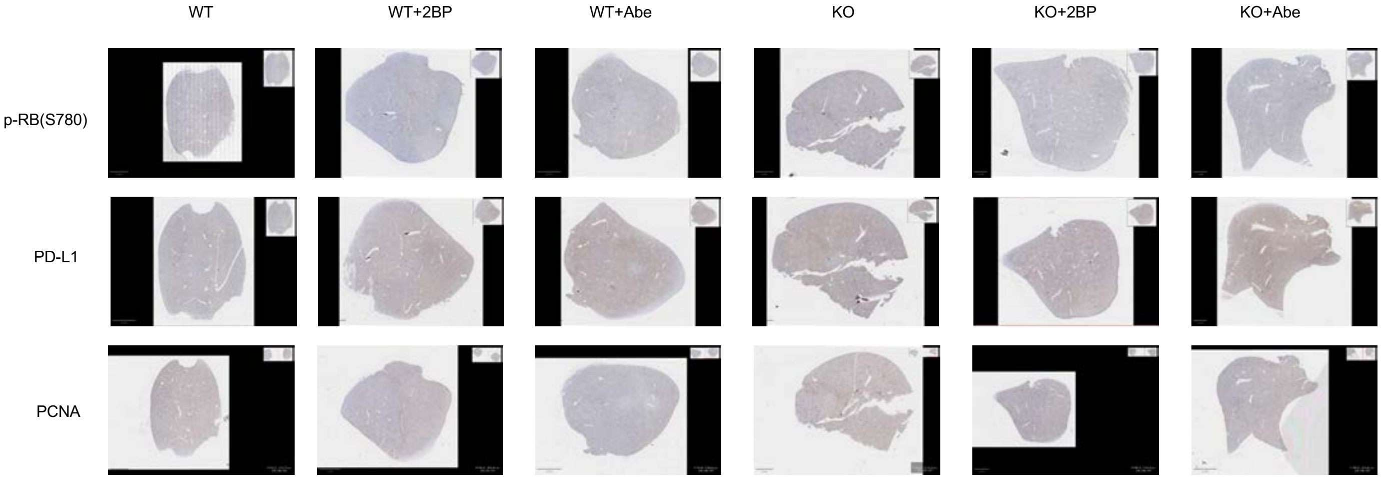
WT
WT+2BP
WT+Abe
KO
KO+2BP
KO+Abe
p-RB(S780)
PD-L1
PCNA

Fig. S1C

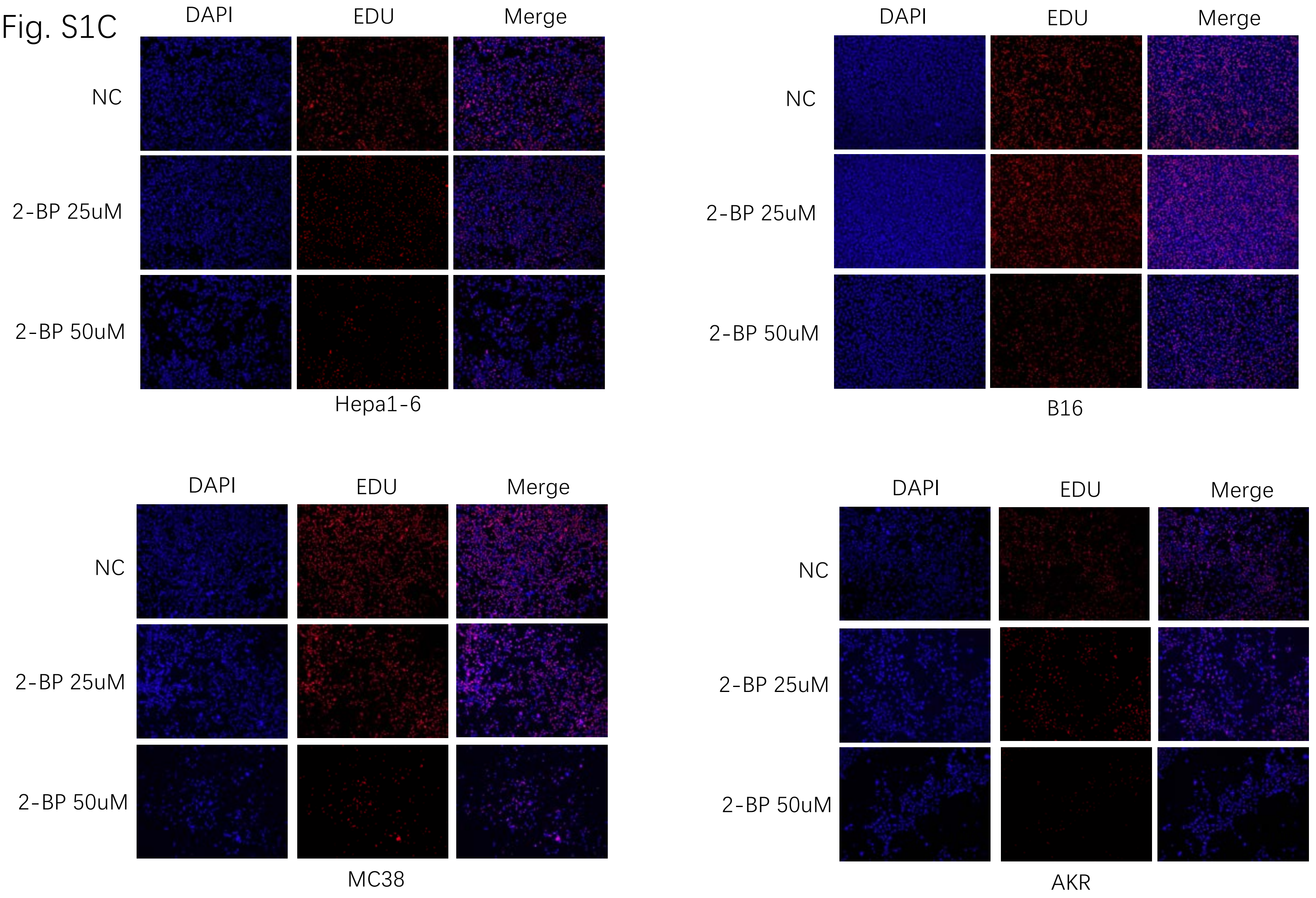
DAPI
EDU
Merge
NC
2-BP 25uM
2-BP 50uM
Hepa1-6
DAPI
EDU
Merge
NC
2-BP 25uM
2-BP 50uM
B16
DAPI
EDU
Merge
NC
2-BP 25uM
2-BP 50uM
MC38
DAPI
EDU
Merge
NC
2-BP 25uM
2-BP 50uM
AKR

Fig. S2G

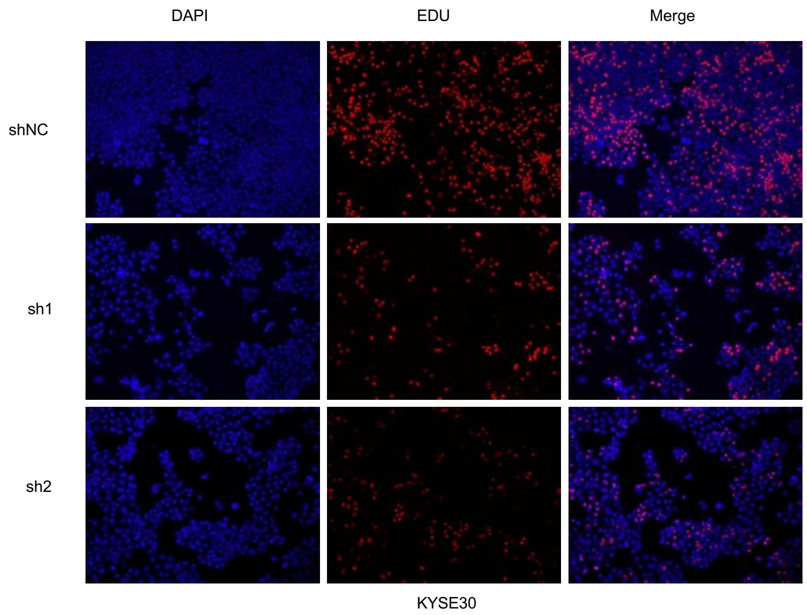

Fig. S2H

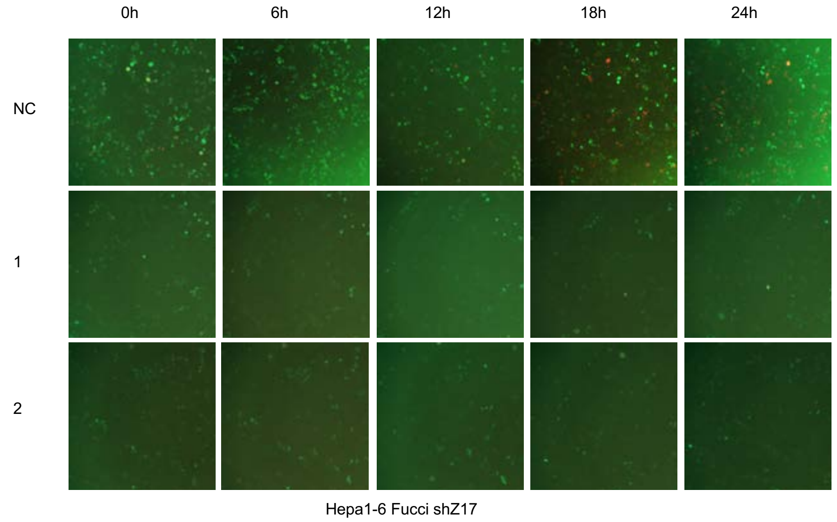

Fig. S4G

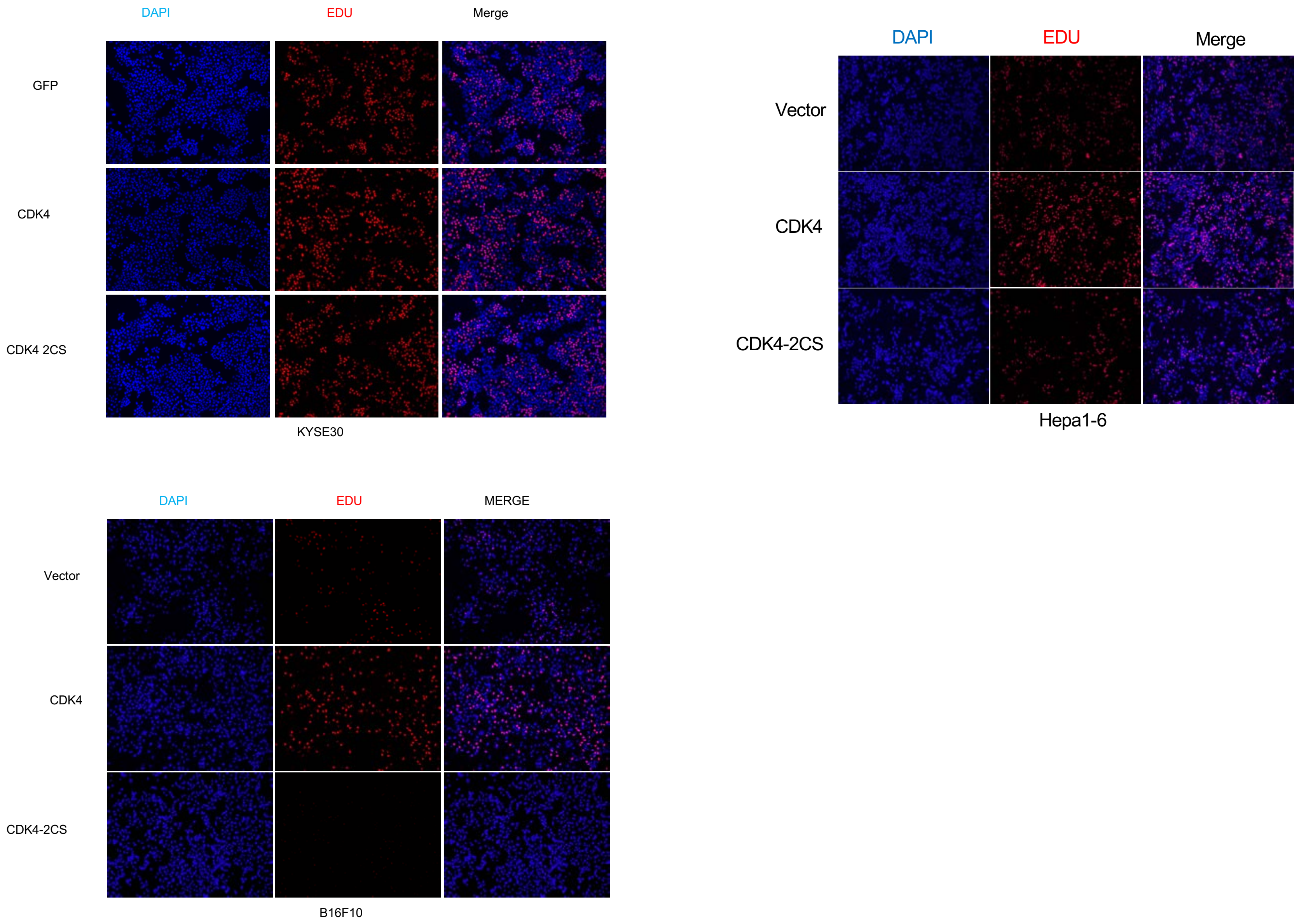

DAPI
EDU
Merge
GFP
CDK4
CDK4 2CS
KYSE30
DAPI
EDU
Merge
Vector
CDK4
CDK4-2CS
Hepa1-6
DAPI
EDU
MERGE
Vector
CDK4
CDK4-2CS
B16F10

Fig. S5A

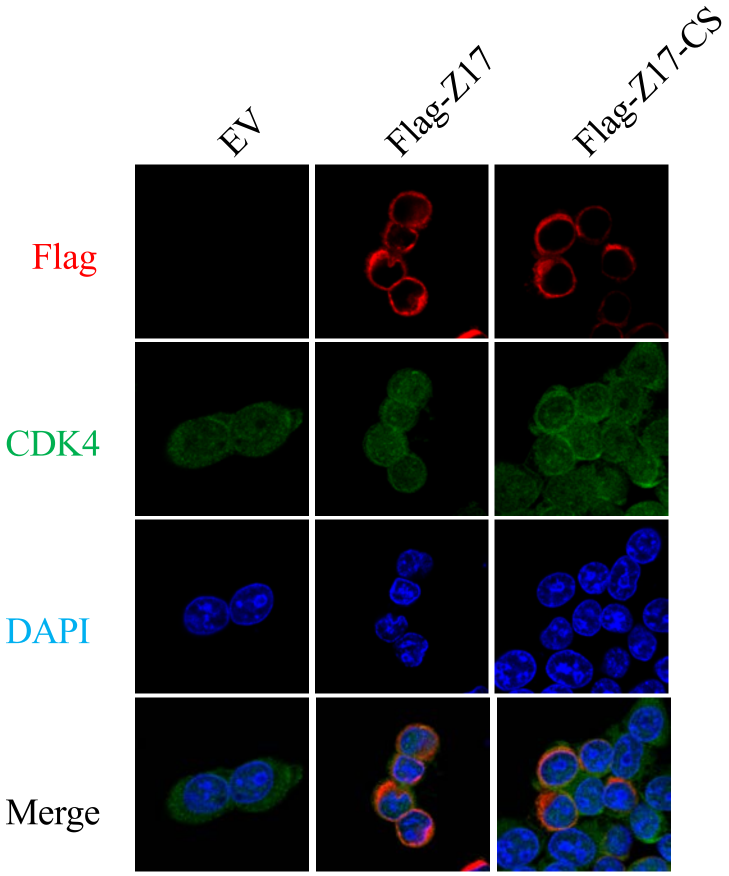

Fig. S5B

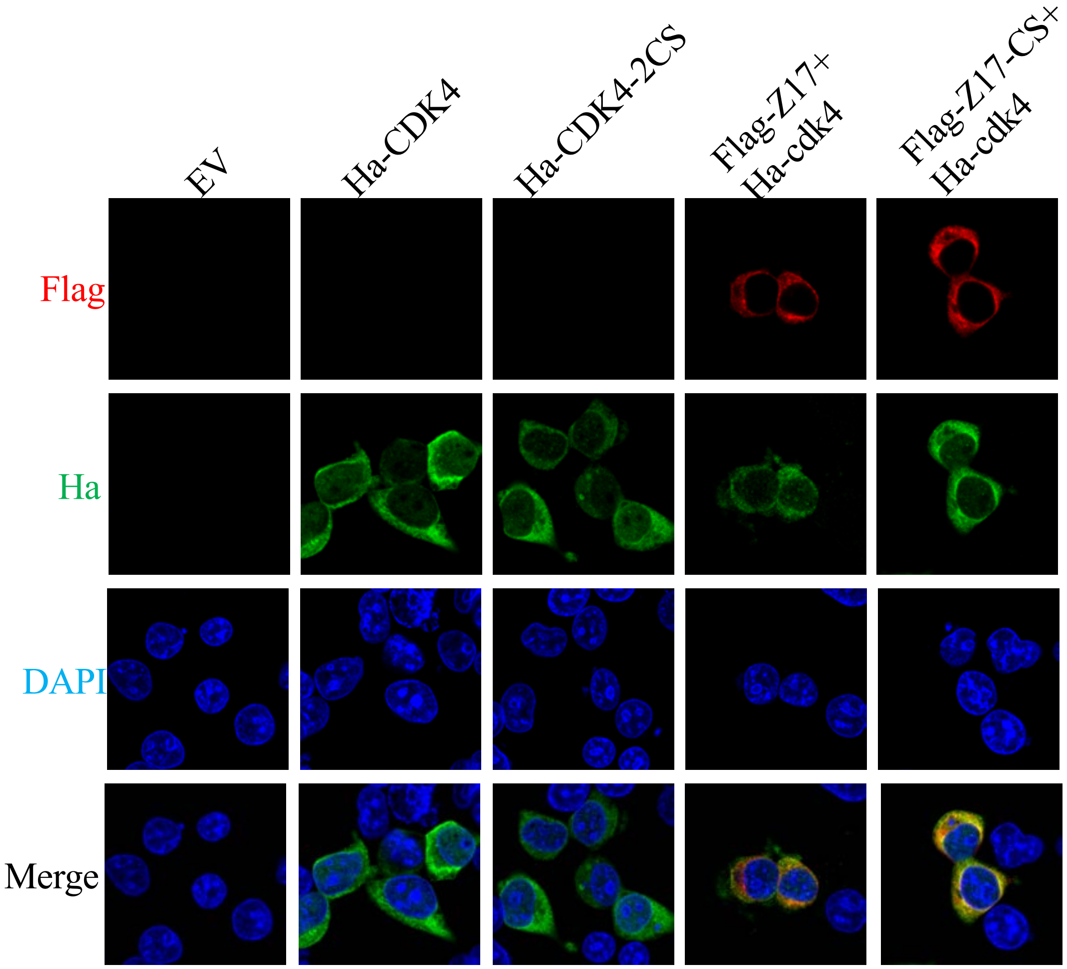

Fig. S5J

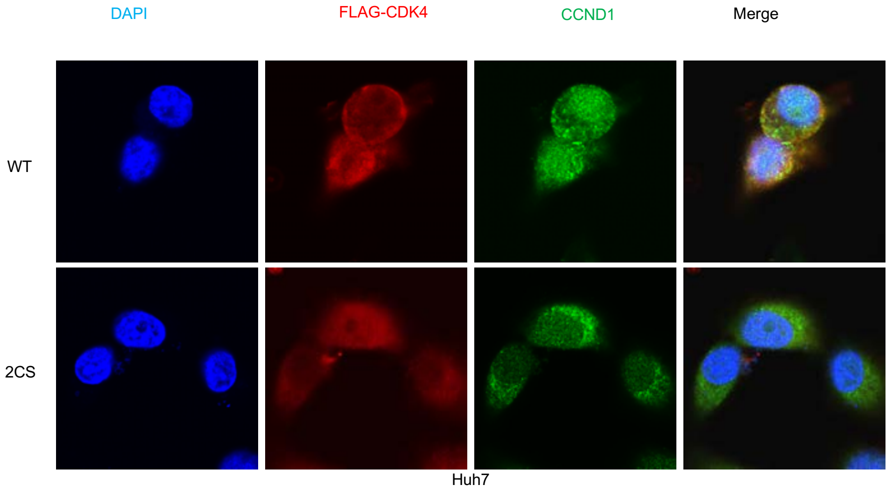

Fig. S7D

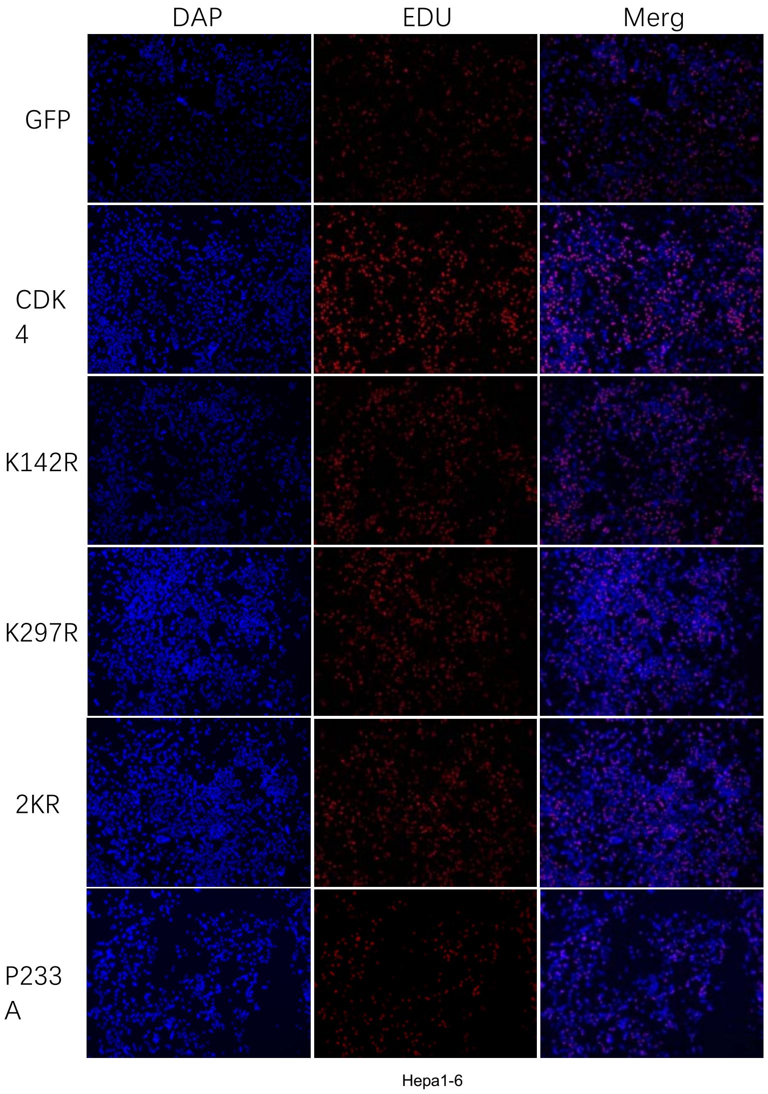
DAP
EDU
Merg
GFP
CDK
4
K142R
K297R
2KR
P233
A
Hepa1-6

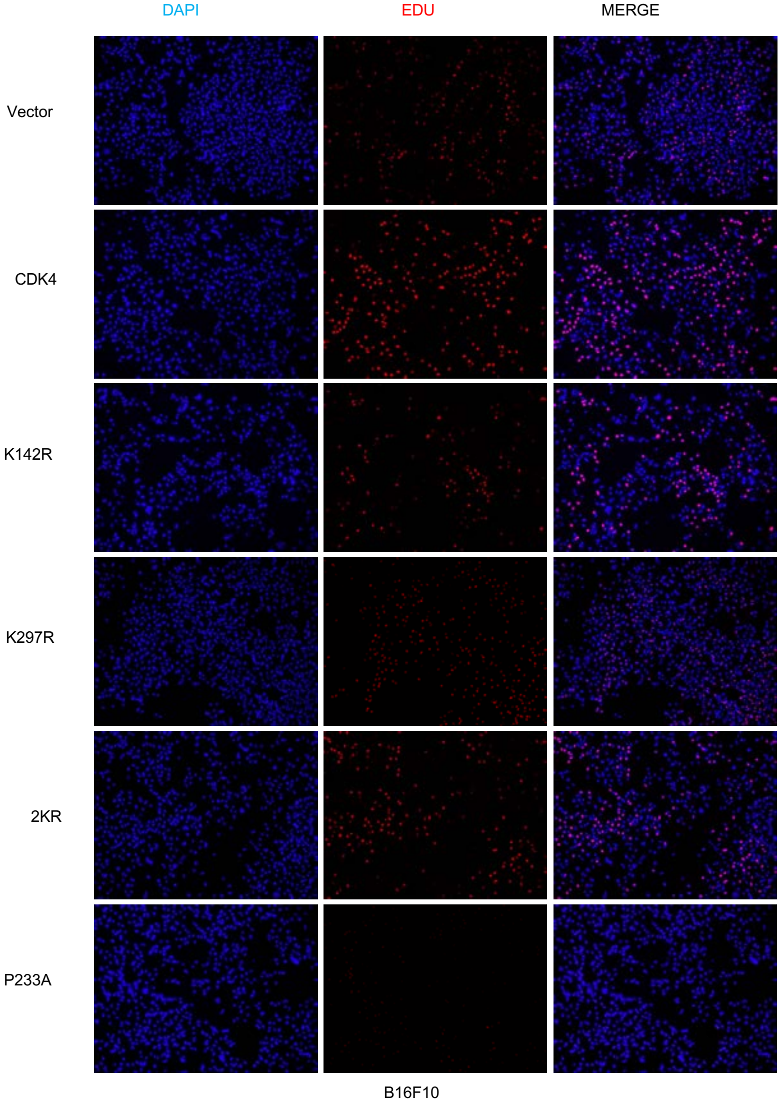
DAPI
EDU
MERGE
Vector
CDK4
K142R
K297R
2KR
P233A
B16F10

Fig. S7D

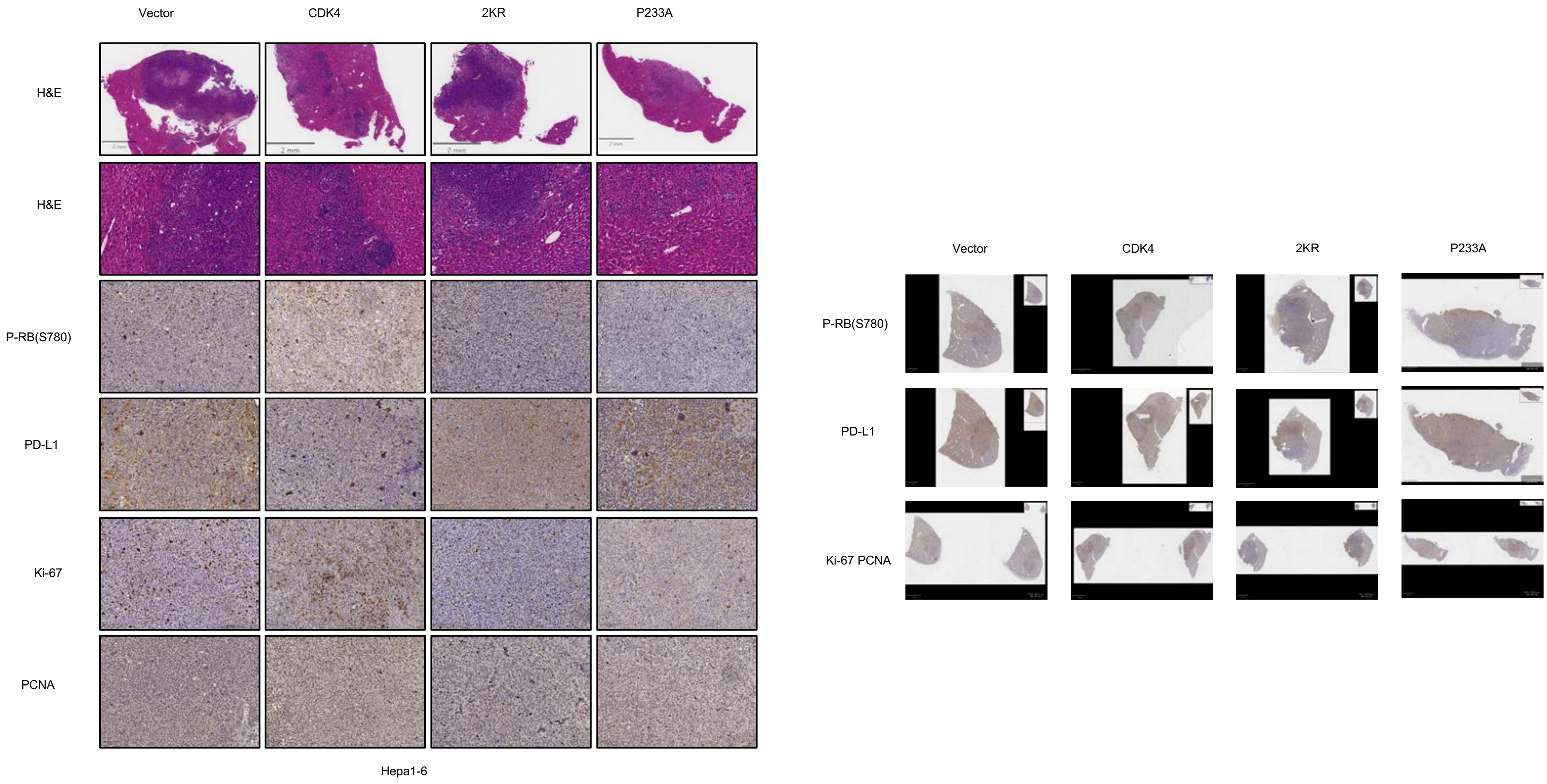

Vector
CDK4
2KR
P233A
H&E
H&E
P-RB(S780)
PD-L1
Ki-67
PCNA
2 mm
Hepa1-6
Vector
CDK4
2KR
P233A
P-RB(S780)
PD-L1
Ki-67 PCNA

Fig.S8D

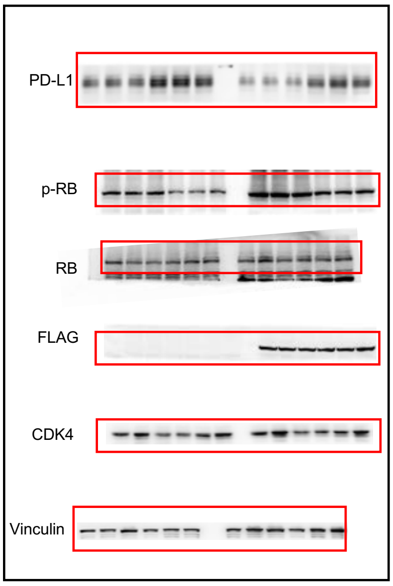

Fig.S8I

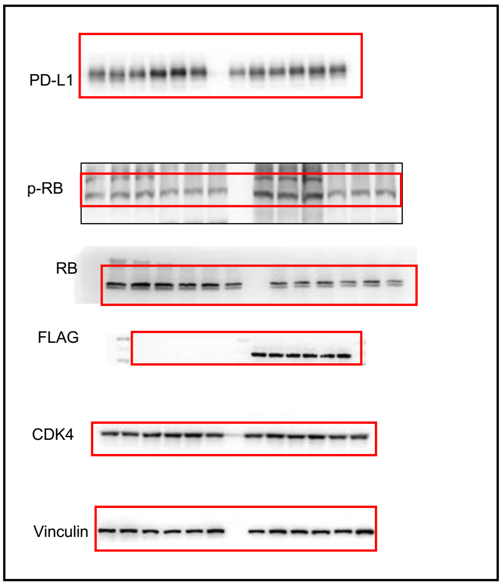

Supplement: Supplementary file 2 — Supporting File 2: advs75693‐sup‐0002‐Data.zip. [file ADVS-9999-e75693-s001.zip › Raw blots data.pdf]
